# An atlas of lamina-associated chromatin across twelve human cell types reveals an intermediate chromatin subtype

**DOI:** 10.1186/s13059-023-02849-5

**Published:** 2023-01-23

**Authors:** Parisha P. Shah, Kathleen C. Keough, Ketrin Gjoni, Garrett T. Santini, Richard J. Abdill, Nadeera M. Wickramasinghe, Carolyn E. Dundes, Ashley Karnay, Angela Chen, Rachel E. A. Salomon, Patrick J. Walsh, Son C. Nguyen, Sean Whalen, Eric F. Joyce, Kyle M. Loh, Nicole Dubois, Katherine S. Pollard, Rajan Jain

**Affiliations:** 1 Departments of Medicine and Cell and Developmental Biology, Penn CVI, Penn Epigenetics Institute, Perelman School of Medicine, University of Pennsylvania, Smilow TRC, 3400 Civic Center Blvd, Philadelphia, PA 19104 USA; 2 University of California, San Francisco, CA 94117 USA; 3 Gladstone Institute of Data Science and Biotechnology, 1650 Owens Street, San Francisco, CA 94158 USA; 4 Department of Cell, Developmental and Regenerative Biology, Icahn School of Medicine at Mount Sinai, New York, NY 10029 USA; 5 Department of Developmental Biology and Institute for Stem Cell Biology and Regenerative Medicine, Stanford University School of Medicine, Stanford, CA 94305 USA; 6 Department of Genetics, Penn Epigenetics Institute, Perelman School of Medicine, University of Pennsylvania, Philadelphia, PA 19104 USA; 7 Chan Zuckerberg Biohub, San Francisco, CA 94158 USA; 8 Smilow TRC, 3400 Civic Center Blvd, Philadelphia, PA 19104 USA

**Keywords:** Lamina-associated domains, Peripheral chromatin organization, 3D genome, Cellular differentiation

## Abstract

**Background:**

Association of chromatin with lamin proteins at the nuclear periphery has emerged as a potential mechanism to coordinate cell type-specific gene expression and maintain cellular identity via gene silencing. Unlike many histone modifications and chromatin-associated proteins, lamina-associated domains (LADs) are mapped genome-wide in relatively few genetically normal human cell types, which limits our understanding of the role peripheral chromatin plays in development and disease.

**Results:**

To address this gap, we map LAMIN B1 occupancy across twelve human cell types encompassing pluripotent stem cells, intermediate progenitors, and differentiated cells from all three germ layers. Integrative analyses of this atlas with gene expression and repressive histone modification maps reveal that lamina-associated chromatin in all twelve cell types is organized into at least two subtypes defined by differences in LAMIN B1 occupancy, gene expression, chromatin accessibility, transposable elements, replication timing, and radial positioning. Imaging of fluorescently labeled DNA in single cells validates these subtypes and shows radial positioning of LADs with higher LAMIN B1 occupancy and heterochromatic histone modifications primarily embedded within the lamina. In contrast, the second subtype of lamina-associated chromatin is relatively gene dense, accessible, dynamic across development, and positioned adjacent to the lamina. Most genes gain or lose LAMIN B1 occupancy consistent with cell types along developmental trajectories; however, we also identify examples where the enhancer, but not the gene body and promoter, changes LAD state.

**Conclusions:**

Altogether, this atlas represents the largest resource to date for peripheral chromatin organization studies and reveals an intermediate chromatin subtype.

**Supplementary Information:**

The online version contains supplementary material available at 10.1186/s13059-023-02849-5.

## Background

Spatial genome organization has emerged as a mechanism for coordinating gene expression towards the specification and maintenance of cellular identity [1, 2]. Approximately 30–40% of the genome is localized to the nuclear lamina, a filamentous network of LAMIN A/C, B1, and B2 proteins residing on the inner surface of the nuclear envelope [3, 4]. Genomic loci localized at the nuclear lamina, termed lamina-associated domains (LADs), occur in kilobase- to megabase-sized blocks [5, 6]. LADs are generally heterochromatic, and genes within LADs are frequently transcriptionally repressed [7, 8]. Mutations in nuclear lamins disrupt chromatin organization and cause disease, and absence of lamins contributes to chromatin inversion [2, 4, 9–14]. Cellular differentiation has been correlated with repositioning of a subset of genes away from the nuclear lamina and their subsequent expression and vice versa [15–17]. More recently, it has been demonstrated that loss of spatial positioning compromises cardiac development [18]. Thus, it is becoming apparent that spatial positioning of the genome is important for development and health.

Previous work indicates that a subset of LADs have varying characteristics, such as reduced lamin occupancy and increased gene density, suggesting that LADs may be heterogeneous across cell types. Comparison of datasets from various mouse and human cell types indicate that LADs can be segregated into cell type-variable (“facultative”) and stable (“constitutive”) domains [6, 19]. Integration of lamin association with core and linker histone occupancy and modifications also distinguished subtypes of LADs within a single murine cell type [20]. Subsequent work in single cells showed that contact frequencies of LADs with the nuclear lamina vary by locus, and single-cell studies suggest that individual genomic regions, in aggregate, have varying probabilities of becoming re-localized to or from the nuclear lamina during differentiation [19, 21, 22], leading to the model predicting different subtypes of LADs. Our understanding of LAD subtypes, however, is still far from complete due to the limited number of contexts that have been studied to date. Further study of lamina-associated chromatin across a greater diversity of cell types, including representation across developmental stages, and identification of key molecular characteristics will provide critical knowledge about how LADs are organized and clues into how spatial positioning may contribute to cellular identity.

To address these gaps, we developed an atlas of LAMIN B1 (LB1) binding across twelve human cell types from all three germ layers and embryonic stem cells (ESCs). Our data revealed two subtypes of lamina-associated chromatin in all examined cell types, one of which is similar to previously described LADs [7, 8] with low chromatin accessibility and gene density, and enrichment for heterochromatic histone modifications. The other subtype has intermediate LB1 enrichment, chromatin accessibility, dimethylation of lysine 9 on histone H3 (H3K9me2), gene density, and gene expression. The LAD subtype with highest LB1 enrichment is positioned within the nuclear lamina, while the intermediate LB1-enriched subtype is positioned both within and adjacent to the nuclear lamina. Unlike most previous approaches, we identified different subtypes of LB1-enriched chromatin regions within many individual, developmentally related human cell types, without the need to compare between cell types. Overall, this work provides the largest atlas of human lamina-associated chromatin maps to date—created from non-immortalized human cells, using cell types derived from common progenitors and representing multiple developmental trajectories—revealing important insights into how association with the lamina may participate in cell type specification and development and providing an important resource for future interrogations of peripheral chromatin.

## Results

### An LB1 ChIP-seq atlas in 12 human cell types from all three germ layers

**Fig. 1:**
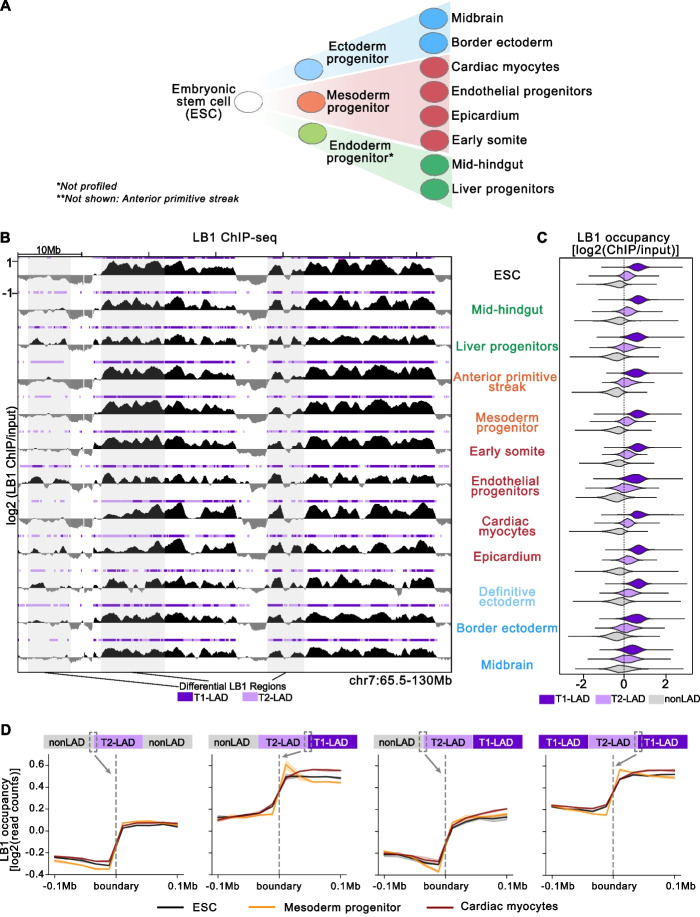
HMM identifies 3 states from LB1 ChIP-seq datasets across 12 human cell types. **A** Simplified overview of progressive lineage restriction indicating some of the cell types represented in the atlas, shown from greatest (left) to least (right) developmental potential. **B** Example track view of LB1 enrichment and associated LADs in all 12 cell types assayed (representative replicate track shown). LAD states defined by HMM per cell type shown above each LB1 track. T1-LADs in dark purple; T2-LADs in light purple. Regions highlighted in gray span differential LB1/LAD regions between cell types. *Y*-axis indicated on the first track is consistent across all tracks shown. **C** LB1 occupancy per LAD state in all 12 cell types. T1-LADs (dark purple) are the most enriched and nonLADs (gray) are the least enriched for LB1. **D** LB1 occupancy measured 100 kb up- and downstream of T2-LAD boundaries with nonLADs and T1-LADs shows sharp distinction of LB1 signal between these f﻿eatures

We generated an atlas of LB1 occupancy from human ESCs and eleven ESC-derived cell types from all three germ layers (endoderm, mesoderm and ectoderm) using established and published differentiation protocols representative of early developmental trajectories [23–27] (Fig. 1A; Additional file 1: Table 1). Expression of key genes measured by quantitative reverse transcription PCR (qRT-PCR; Additional file 2: Fig. S1A-F), fluorescence-activated cell sorting, and immunofluorescence for cell type-specific markers (Additional file 2: Fig. S1G-M) in various cell types supported our established differentiation strategies [23, 25, 26, 28, 29]. Cardiac cultures also displayed stereotypical contractile activity (Additional file 3: Video 1; Additional file 4: Video 2). We performed LB1 chromatin immunoprecipitation (ChIP) using an antibody that has been previously validated for ChIP in human cells [12, 30, 31]. We further verified the antibody in select cell types by performing immunoblotting following LB1 ChIP under standard conditions and with the addition of a LB1 peptide, which quenched LB1 immunoprecipitation (Additional file 2: Fig. S2A, B). We chose ChIP-seq rather than DamID to map LB1 across multiple stages of differentiation, because it reflects targeted protein binding at fixation (versus DNA adenine methylation that occurs over several hours) and circumvents differences in methylation efficiency or differentiation that may occur when using DAM-modified cells [22, 32, 33]. Each biological replicate was sequenced to greater than 40 million uniquely mapping reads, and the Pearson correlation between replicates was greater than 0.7 for 90% of cell types, indicating high reproducibility between replicates (Additional file 1: Table 1). Visual inspection of the atlas LB1 ChIP-seq data on a genome browser confirmed the presence of large, discrete LB1-enriched domains consistent with the presence of LADs in all cell types investigated (Fig. 1B).

### A three-state hidden Markov model identifies two subtypes of LB1-enriched domains

To aid our interpretation of variable LB1 occupancy across the genome, we implemented hidden Markov models (HMMs) in each cell type to identify regions of LB1 enrichment (e.g., LADs) from LB1 ChIP-seq datasets (see “Methods”). We chose an HMM approach over broad peak callers like Enriched Domain Detector [34] to circumvent potential bias from the necessary manual parameter tuning across such a large dataset. In addition, such broad peak callers often only identify binary LAD and nonLAD states, and previous studies suggest that LADs may exist in multiple states [20]. HMMs have previously been used to segment chromosomes into LAD versus nonLAD regions utilizing DamID-generated LB1 binding peaks and to segment chromatin states based on various epigenetic marks [6, 35, 36]. We compared model performance for two through five states to determine the number of distinct domain types that best represented the data (Additional file 2: Fig. S2C, D). Our goal was to fit the simplest model that most accurately described the data (see “Discussion”). The model performance was most improved in the fit from three versus two states, which suggested the existence of an intermediate state of lamina-associated chromatin in all 12 human cell types, consistent with previous observations of an intermediate LAD state in a single cell type studied [20]. We therefore proceeded with a three-state HMM to analyze regions of LB1 enrichment (and depletion) from each cell type. A separate model was fit to each cell type in order to account for differences in LB1 signal across biological contexts and batches, enabling us to identify LB1-enriched regions compared to adjacent LB1-depleted genomic regions.

We designated the HMM states as Type 1 LADs (T1-LADs), Type 2 LADs (T2-LADs), and nonLADs in descending order of LB1 occupancy (Fig. 1B, C; Additional file 5: Table 2). T1-LADs are slightly larger [median across cell types: 290 kilobases (kb)] than T2-LADs (median: 280 kb) overall, though domain sizes vary for both subtypes (Additional file 2: Fig. S2E). T1-LADs and T2-LADs respectively cover 31.5 and 35.3% of the genome on average across cell types. ChIP-seq mapped LADs represent clusters of LB1-enriched regions interspersed with gaps (local regions of LB1 depletion) or regions with reductions in LB1 signal [34]. Therefore, to validate the HMM results, we first confirmed that almost all T1-LAD and a majority of T2-LAD regions are LB1-enriched across atlas cell types (log2 LB1/input > 0 per 20-kb bin; Additional file 2: Fig. S3A). In a complementary approach, nearly all (>99%) narrow LB1 peaks identified by epic2 (*q*<0.05) overlapped with HMM-identified T1-LAD and T2-LAD regions in both ESCs and CMs. Second, we confirmed that LB1 enrichment in T2-LADs was not an artifact of input normalization (Additional file 2: Fig. S3B). We observed significantly more non-normalized LB1 signal in T2-LADs compared to nonLADs. Third, we repeated the HMM in each biological replicate per cell type across the atlas and compared the base pair (bp) to bp overlap between the identified features in each replicate (Additional file 2: Fig. S3C). Nearly all cell types showed a high degree of concordance. Fourth, we confirmed the differential LB1 enrichment between T1-LADs and T2-LADs, and relative LB1 depletion in nonLADs, by LB1 ChIP-qPCR across 20 different T1-, T2-, and nonLAD regions (Additional file 2: Fig. S3D). Finally, we compared HMM-identified ChIP-seq LADs to existing DamID-identified LADs for additional validation. It has been previously shown [18, 31] that ChIP-seq-identified LADs have a high degree of overlap with DamID-identified LADs. We compared our ESC datasets to publicly available hESC DamID-LAD maps (4DN Data set Identifier: 4DNESNFNTUAO). As expected, we found a high degree of overlap in Dam control-normalized DamID to ChIP-seq track comparisons, including regions of low DamID enrichment in areas identified as T2-LADs (Additional file 2: Fig. S3E). A bp to bp overlap assessment showed that nearly all (99.6%) bases within DamID LADs overlapped HMM-identified LADs from ESCs profiled in this study (Additional file 2: Fig. S3F). Reciprocally, most T1-LAD bases overlap DamID LADs (98.1%) and nearly half of T2-LAD bases (47.4%) overlap DamID LADs. Accordingly, DamID signal (log2 DamID/Dam control) across HMM-identified LAD subtypes also showed lower LB1 enrichment in T2-LADs compared to T1-LADs, mirroring our observations in the LB1 ChIP-seq data (Additional file 2: Fig. S3G, compare to Fig. 1C). Similar to previous studies [6, 37], we also observed that T1-LADs have the lowest GC content, followed by T2-LADs, and then nonLADs for each cell type (Additional file 2: Fig. S3H). LB1 enrichment sharply changed across nonLAD:T2-LAD and T1-LAD:T2-LAD transitions, with marked changes in LB1 occupancy on either side of the boundary in select cell types used for further interrogation (Fig. 1D; see below). The sharp LB1 occupancy changes at boundaries indicate distinct transitions between LAD states. Notably, HMM transition parameters from each cell type-specific model demonstrated the stability of HMM-identified domains in each designated state (Additional file 2: Fig. S4). Thus, using LB1 ChIP-seq and cell type-specific three-state HMMs, we identified two subtypes of LADs in 12 human cell types.

### T1- and T2-LADs have distinct genomic features

**Fig. 2:**
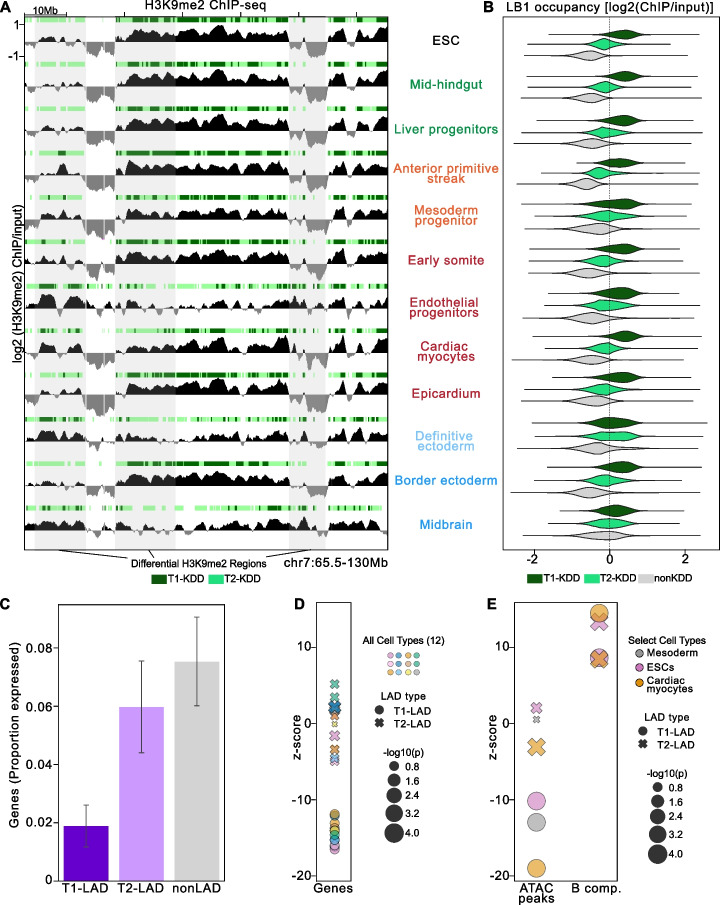
LADs correspond closely with KDDs. **A** Example track view of H3K9me2 enrichment and associated KDDs in all 12 cell types assayed. KDD states as defined by HMM per cell type shown above each H3K9me2 track. T1-KDDs in dark green; T2-KDDs in light green. Regions highlighted in gray span differential H3K9me2/KDD regions between cell types. *Y*-axis indicated on the first track is consistent across all tracks shown. **B** LB1 occupancy per KDD state in all 12 cell types. T1-KDDs (dark green) are the most enriched, and nonKDDs (gray) are the least enriched for LB1. **C** Mean proportion of genes expressed (TPM > 5) per LAD category for cell types with matched RNA-seq data (cardiac myocytes, early somites, mesoderm progenitors, mid-hindgut, definitive ectoderm, and endothelial progenitors). Error bars indicate standard deviation. **D** Enrichment (positive *z*-score) or depletion (negative *z*-score) of genes. Cell types in key are as follows: Row 1 (L-R): ESC, liver progenitors, epicardium, endothelial progenitors; Row 2 (L-R): mid-hindgut, border ectoderm, anterior primitive streak, midbrain; Row 3 (L-R): cardiac myocytes, definitive ectoderm, early somite, mesoderm progenitors. **E** Enrichment of ATAC-seq peaks [39], and B compartment (B comp.) from matched cell types [40] (see “Methods”)

We hypothesized that T1- and T2-LADs would differ in other epigenomic features, in addition to differential LB1 enrichment. We first assessed for co-occupancy of H3K9me2, a histone modification enriched in lamina-associated chromatin [12, 18, 21, 38]. We generated H3K9me2 ChIP-seq maps (Fig. 2A) using an antibody we previously extensively validated for specificity for H3K9me2 with modified histone peptide arrays [12, 18]. H3K9me2 ChIP-immunoblot (Additional file 2: Fig. S5A) and peptide immunoblot (Additional file 2: Fig. S5B) also indicated specificity for H3K9me2 as opposed to closely related modifications. We applied similar HMM fitting to the H3K9me2 data, again finding a 3-state model to be the simplest, best-fitting model that captures a large proportion of the variability in our ChIP-seq data (Additional file 2: Fig. S5C, D). These states were designated T1-, T2-, and nonKDDs (K9 dimethyl domains) based on decreasing median H3K9me2 enrichment (Additional file 2: Fig. S6A, Additional file 6: Table 3). H3K9me2 ChIP-qPCR confirmed differential H3K9m2 enrichment at different types of KDDs (Additional file 2: Fig. S6B), and bp to bp overlap assessments between individual replicate HMM results showed high concordance of features (Additional file 2: Fig. S6C). KDDs showed a similar size range as LADs, with T2-KDDs having an overall smaller median size than T1-KDDs (Additional file 2: Fig. S6D). Transition state parameter stability calculations supported a three-state model (Additional file 2: Fig. S7). Across cell types, T1- and T2-KDDs demonstrated a high degree of overlap with T1- and T2-LADs, respectively (Additional file 2: Fig. S8A, B). Accordingly, LB1 occupancy was most often highest in T1-KDDs, followed by T2-KDDs and nonKDDs (Fig. 2B). This analysis confirmed that greater chromatin association with LB1 coincides with increasing H3K9me2 occupancy across the 12 cell types.

We next investigated additional features of lamina-associated chromatin across cell types. Compared to T2- and nonLADs, T1-LADs had the lowest proportion of expressed genes based on RNA-seq generated for this study and from published sources from matched cell types (Fig. 2C, Additional file 2: Fig. S8C) [25, 41, 42]. T1-LADs also showed greater gene depletion compared to T2-LADs (Fig. 2D). While both subtypes of LADs are heterochromatic, as evidenced by enrichment for B compartment regions [43], T2-LADs were more enriched for ATAC-seq peaks [39] than T1-LADs (Fig. 2E). Thus, T2-LADs are intermediately enriched between T1-LADs and nonLADs for gene expression, gene density, and accessibility across multiple cell types (Fig. 2C–E).

**Fig. 3:**
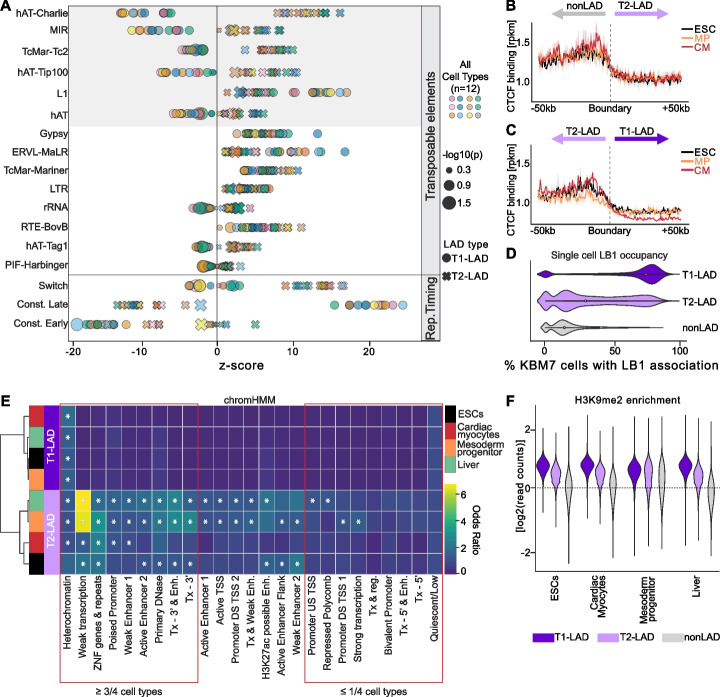
Genomic characterization of LAD subtypes indicates T1- and T2-LAD distinction. **A** Enrichment of transposable elements (top) and replication timing (RT) domains (bottom) in T1- and T2-LADs across the atlas. Transposable elements differentially enriched in T1- and T2-LADs highlighted in gray. Cell types in key are as follows: Row 1 (L-R): ESC, liver progenitors, epicardium, endothelial progenitors; Row 2 (L-R): mid-hindgut, border ectoderm, anterior primitive streak, midbrain; Row 3 (L-R): cardiac myocytes, definitive ectoderm, early somite, mesoderm progenitors. **B,C** CTCF binding at transitions from **B** nonLADs to T2-LADs (including mirrored T2-LAD to nonLAD boundaries) and **C** T2-LADs to T1-LADs (including mirrored T1-LAD to T2-LAD boundaries) shows CTCF enrichment at T2-LAD boundaries. **D** Percentage of single cells with LB1 occupancy per LAD locus in mesoderm progenitor cells shows greater overlap with T1-LADs. **E** chromHMM feature enrichments in LADs from four cell types, as indicated. Asterisks (*) indicate adjusted *p*-value < 0.01. **F** H3K9me2 occupancy per LAD state in selected cell types, as indicated (full set in Additional file 2: Fig. S6A). T1-LADs (dark purple) are the most enriched and nonLADs (gray) are least enriched for H3K9me2

Motivated by these results, we sought to better understand distinguishing characteristics between T1- and T2-LADs. We first determined that specific types of transposable elements show divergent patterns of enrichment in T1- versus T2-LADs, though L1 and ERVL-MaLR elements are consistently enriched in both LAD subtypes (Fig. 3A, shaded upper box versus non-shaded box). Next, we identified that T1- and T2-LADs were depleted for constitutively early replicating domains compared to nonLADs [44]; however, only T1-LADs were enriched for constitutive late replication timing domains, while T2-LADs were enriched for switch replication timing domains (Fig. 3A, lower box). In addition, we assessed binding sites of the chromatin insulator CTCF from comparable cell types [40] and observed that CTCF occupancy peaked proximal to transitions from states of lower to higher LB1 (i.e., from nonLAD to T2-LAD or T2-LAD to T1-LAD) and decreased sharply at the boundary (Fig. 3B, C), consistent with previous results [5, 45–47]. Additionally, T1-LADs had greater overlap with all LB1-associated regions defined by single-cell DamID in a comparable cell type [19, 22] compared to T2-LADs, suggesting that at least a subset of T2-LADs may have a more variable or intermediate association with the lamina in a population of cells (Fig. 3D).

In order to integrate the findings, we turned to chromHMM, a platform that can annotate different states based on various epigenetic and related characteristics [36]. T1- and T2-LADs were significantly enriched for the chromHMM heterochromatin state [36] in all cell types, except for T2-LADs in ESCs, which did not meet statistical significance. Overall, T2-LADs harbored a more diverse group of states than T1-LADs. Most notably, T2-LADs were consistently depleted of bivalent promoters, repressed polycomb chromatin, strong transcription regions, and related states (at least 3 of 4 cell types). Conversely, T2-LADs were significantly enriched for weak transcription, zinc finger genes, and repeats in all four cell types, and significantly enriched for multiple enhancer states in at least three of four cell types (Fig. 3E). Consistent with the chromHMM output and the close alignment of LB1 and H3K9me2, we found H3K9me2 most often showed the highest enrichment in T1-LADs, followed by T2-LADs and then nonLADs (Fig. 3F, Additional file 2: Fig. S8B).

Finally, as mentioned above, comparative analysis of LADs from different cell types have identified a subset of LADs that are different between them and are therefore likely cell type-specific. We compared our ESC T1- and T2-LADs to “facultative” cell type-specific LADs (fLADs) and “constitutive” cell type-invariant LADs (cLADs) defined in 9 immortalized human cell lines [19, 22]; ESCs were the only shared cell type between the atlas and this dataset. The majority of ESC T1-LADs were classified as cLADs, but some T1-LADs in ESCs were classified as fLADs in the DamID 9-cell categorization (Additional file 2: Fig. S8D). T2-LADs were overwhelmingly categorized as fLADs. Notably, fLADs were originally identified by differential LAD enrichment between cell types. In contrast, T2-LADs are defined *within* a cell type, suggesting that these two LAD subtypes may be related, but are not necessarily the same. These findings suggest that T1- and T2-LADs are distinct entities, where T1-LADs are high LB1 enrichment/contact frequency domains and T2-LADs are intermediate peripheral domains with moderate LB1 contact frequency. To further characterize this difference, we considered whether differential LB1 enrichment between T1-LADs and T2-LADs may reflect fewer or shorter stretches of direct LB1-DNA contacts in T2-LADs compared to T1-LADs. We assessed for “gaps” in LB1 enrichment in ESCs and cardiac myocytes (Additional file 2: Fig. S8E). The proportion of LB1-enriched (log2 LB1/input > 0) windows in T2-LADs decreased as window size increased, indicative of gaps in LB1 enrichment, providing additional support to indicate that T2-LADs are an intermediate peripheral domain with moderate LB1 contact frequency relative to T1-LADs.

### T2-LADs are spatially positioned between T1-LADs and nonLADs

**Fig. 4:**
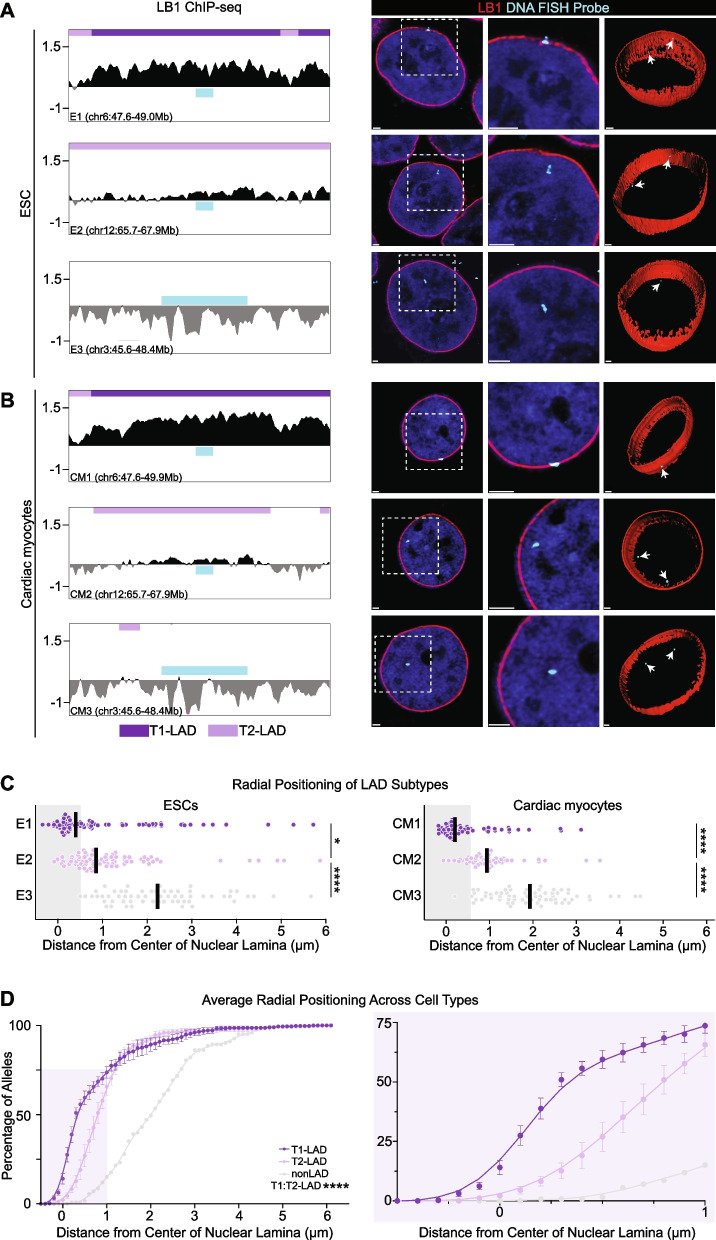
T1-LAD, T2-LAD and nonLAD regions are distinctly radially positioned within the nucleus. **A, B** (Left) T1-LAD, T2-LAD, and nonLAD FISH probe locations in LB1 tracks in **A** ESCs and **B** cardiac myocytes. Probe indicated by blue box. Each probe in **A** and **B** assigned a unique identifier per cell type (E1-E3 and CM1-CM3; see also Additional file 2: Fig. S9A, F). (Right) Representative images of FISH foci (individual slices from Z-stack) from each tested probe region with 3D renderings of all slices to the right for ESCs **A** and fluorescence-activated cell sorted cardiac myocytes **B**. Box in the left image indicates the approximate area of the high magnification image in the middle. **C** Quantification (see “Methods”) of the distance of each FISH focus to the center of the nuclear lamina for the probes (noted by unique identifier) shown in **A** and **B** (see Additional file 2: Fig. S9A, B, F-H) relative to the middle of the nuclear lamina (LB1). Gray box indicates the depth of H3K9me2-marked heterochromatin at the nuclear periphery (see “Methods” and Additional file 2: Fig. S9D). **D** Cumulative frequency distribution of all probes imaged in study. Data in the light purple box on the left highlighted on the right. Error bars represent 1 SEM. Statistical significance of the differences in localization between T1-, T2-, and nonLAD foci in **C** calculated by a Kruskal-Wallis test with post hoc Dunn test for multiple comparisons. Statistical significance of differences in distribution between T1- and T2-LAD foci in **D** was calculated by a Kolmorgov-Smirnov test. *p<0.05, ****p<0.0001. Scale bars = 1μm

The above analyses raised the possibility that T1- and T2-LADs could be differentially localized within the nucleus. We predicted T1-LADs would be spatially positioned at the nuclear lamina, given that this LAD subtype has the highest LB1 occupancy, and we predicted nonLADs would rarely be positioned within the layer of peripheral chromatin. It was not clear whether the intermediate LB1 occupancy of T2-LADs (Additional file 2: Fig. S8E) was a reflection of T2-LADs being heterogeneously positioned relative to the lamina across cells (as suggested by single-cell DamID and related studies [19, 22]), or of T2-LADs being peripherally positioned compared to nonLADs, but consistently positioned farther away from the nuclear lamina compared to T1-LADs. The latter possibility would be consistent with a recent integrated analysis of DamID and TSA-seq data from K562 cells, which suggested that loci consistently fall into specific locations within the nucleus [48]. To distinguish between the two possibilities, we performed immunofluorescence of LB1 coupled with oligo-based fluorescence in situ hybridization (IF-FISH) [49] to visualize the nuclear lamina and multiple T1-LAD, T2-LAD, and nonLAD regions (Fig. 4).

We first selected three candidate loci classified as T1-LAD, T2-LAD, and nonLAD in both ESCs (E1, E2, E3, respectively) and cardiac myocytes (CM1, CM2, CM3, respectively; Fig. 4A–C). We designed FISH probes to LADs in the 20–70th percentile range for LB1 strength to maximize interpretability of the data (Additional file 2: Fig. S9A, B; see “Methods” for additional details of FISH probe design). We first performed IF-FISH and measured the shortest 3D Euclidean distance of the FISH foci to the nuclear lamina in ESCs (see “Methods,” Fig. 4A). T1-LAD foci were often embedded within the nuclear lamina in ESCs, and nonLAD foci were rarely positioned at the nuclear lamina [Fig. 4A, C, *n*=50 and *n*=34 ESC nuclei for the T1-LAD (E1) and nonLAD (E3) probes, respectively]. Interestingly, the majority of foci for the T2-LAD probed region (E2) were positioned just adjacent to the nuclear lamina (Fig. 4A, C, *n*=48 nuclei). IF-FISH to additional T1-LAD and T2-LAD loci (E4 and E5, respectively) provided additional support for the observation of intermediate T2-LAD positioning (Additional file 2: Fig. S9B, C; *n*=30 and *n*=49 ESC nuclei for E4 and E5, respectively). DamID profiles from ESCs correlated well with LB1 ChIP-seq from ESCs across and surrounding the regions to which probes were designed (Additional file 2: Fig. S9B). We also confirmed the enrichment of H3K9me2-marked chromatin at the nuclear periphery in ESCs by IF (Additional file 2: Fig. S9D). The approximate depth of H3K9me2-marked peripheral chromatin was consistent with previous studies in murine cells [18]. We found more T1-LAD foci within this layer of peripheral heterochromatin compared to T2-LAD foci. In contrast, nonLAD foci were rarely in this layer of peripheral chromatin (Fig. 4C; Additional file 2: Fig. S9C, D; depth in gray box based on the maximum H3K9me2 depth across 5 random measurements from *n*=10 independent nuclei—see “Methods”).

To extend our findings beyond one cell type, we repeated the FISH experiments and analyses with fluorescence-activated cell sorted cardiac myocytes (Fig. 4B, Additional file 2: Fig. S9E). Strikingly, the IF-FISH results were nearly identical between cardiac myocytes and ESCs (Fig. 4B, C; *n*=35, *n*=30, and *n*=36 nuclei for the T1-, T2-, and nonLAD probes, respectively; Additional file 2: Fig. S9F). The majority of T2-LAD probed regions were specifically positioned just adjacent to the nuclear lamina (Fig. 4C). IF-FISH to additional T1-LAD and T2-LAD loci (CM4 and CM5) also showed an intermediate positioning phenotype for T2-LADs (Additional file 2: Fig. S9F-H; *n*=31 and *n*=41 nuclei for CM4 and CM5, respectively). Moreover, consistent with the ESC imaging, we observed more T1-LAD foci within the layer of peripheral chromatin marked by H3K9me2 (Additional file 2: Fig. S9D) compared to T2-LAD foci, but rarely observed nonLAD foci in this layer (Fig. 4C; Additional file 2: Fig. S9H, gray box). Given the concordant results across cell types, we then averaged the measured Euclidean distances of all T1-, T2-, and nonLAD probes in both cell types and observed intermediate positioning of T2-LADs between T1- and nonLADs (Fig. 4D), independent of probe or cell type. The imaging data, taken collectively with the molecular characteristic assessments above, indicate T1-LADs and T2-LADs are distinct features that can be defined within and across cell types and show distinct radial positioning, with T2-LADs occupying an intermediate peripheral position between T1-LADs and nonLADs relative to the lamina.

### Most T1- and T2-LADs vary across cell types

**Fig. 5:**
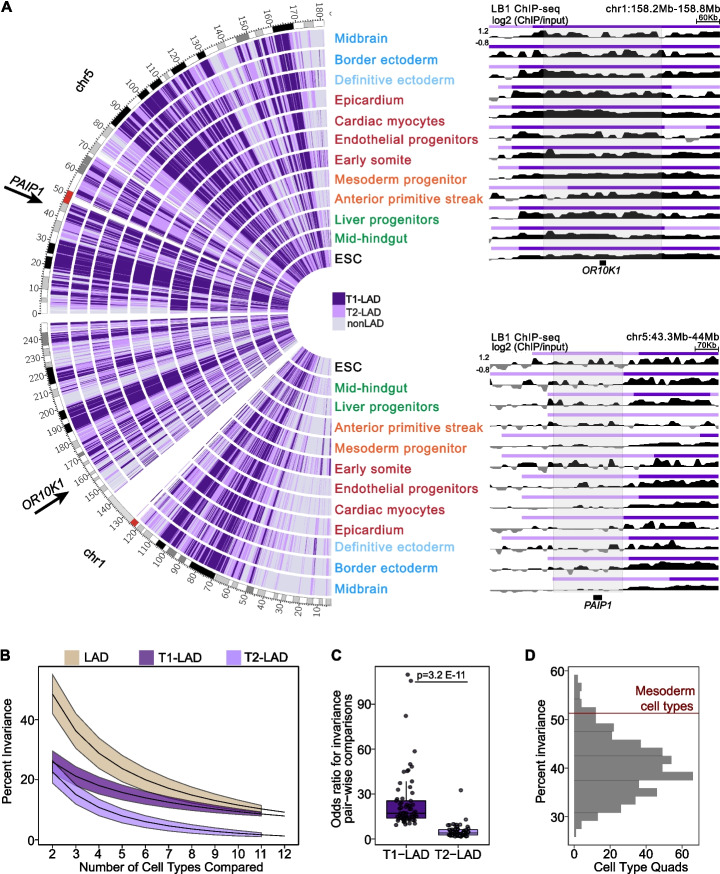
A subset of T1- and T2-LADs are invariant across cell types. **A** (Left) LAD assignments across chromosomes 1 and 5 compared across the atlas. T1-LADs in dark purple, T2-LADs in light purple, nonLADs in gray. Arrows indicate example genes located in invariant T1- and T2-LADs. (Right, top) Genome browser LB1 tracks of the *OR10K1* locus, located in an invariant T1-LAD. (Right, bottom) Genome browser LB1 tracks of the *PAIP1* locus, located in an invariant T2-LAD. GO categories associated with these genes are shown in Additional file 7: Table 4. Relative LB1 enrichment (*Y-*axis) is indicated on the first track and is consistent across all tracks shown on the right. **B** Percent of invariant LADs across random comparisons from 2 to 12 cell types shows decreased LAD invariance as cell type numbers increase, with T1-LADs being more invariant than T2-LADs, when stratified by LAD subtype. Lines represent means and colored ribbons represent standard deviation. Beige line represents T1- and T2-LADs combined. **C** Cell type pair comparisons of T1-LAD and T2-LAD variation show significantly higher invariance for T1-LADs across all possible comparisons compared to T2-LADs. *T*-test was used to calculate significance. **D** Comparison of genome-wide invariance of HMM calls in all combinations of 4 cell types, with the non-progenitor-related mesoderm cell types marked by a red line at the 96th percentile of the distribution

Previous studies have shown that many LADs are shared between cell types [6, 37]; however, these studies are often limited to comparisons between two or three cell types along a single differentiation trajectory or across cells that are mostly terminally differentiated/immortalized. The atlas represents many developmentally diverse cell types, and we therefore hypothesized that invariant LADs, genomic regions which are conserved as the same LAD subtype across cell types, would be the exception rather than the rule in our atlas. Indeed, we identified only 7.9 and 1.3% of the assigned human genome (portion of the genome assigned to a state in the HMM model, ~90% of the total genome) encompassed in invariant T1- and T2-LADs across the atlas, respectively (representative chromosomes shown in Fig. 5A, left). We calculated the percentage of the invariance across different combinations of 2–12 cell types for T1- and T2-LADs (individually and combined, Fig. 5B) and observed a reduction in genome invariance with increasing numbers of cell types, with the largest reduction occurring between the two to three cell type comparisons (mean of 48% invariance across 2 random cell types versus 36% invariance across 3 random cell types). When stratified by LAD subtype, T1-LADs showed greater invariance than T2-LADs both overall and within a smaller range across the number of cell types compared (Fig. 5B). When comparing cell type pairs, the invariance of T1- and T2- LADs is similar (26 and 23%, respectively). As the number of cell types increases, the invariance is increasingly driven by T1-LADs. With 12 cell types, the vast majority of invariant regions are T1-LADs. Given this, we tested the expectation that T1-LADs are more stable in any cell type pairwise comparison compared to T2-LADs and found significantly higher odds of invariance for T1-LADs compared to T2-LADs (Fig. 5C; Additional file 2: Fig. S10A—see “Methods”). Thus, even though T2-LADs are defined based on LB1 signal within a single cell type, we conclude they are more likely than T1-LADs to vary across cell types.

Given the association between a subset of LADs and cellular identity [12, 17, 18], we next examined whether the variety of developmental states in the atlas is linked to the limited amount of genomic space encompassed by invariable LADs. It is possible that closely related cell types (along a generally similar lineage trajectory) have less genome-wide invariance (across LAD and nonLAD categorizations) than cell types with greater developmental differences. Alternatively, LAD organization may be linked to identity in differentiated cell types but not follow a path correlating with identity during lineage restriction. The atlas dataset provided an opportunity to directly test these possibilities. We compared the amount of invariance between LADs and nonLADs from the four more developmentally related differentiated mesoderm cell types (germ layer with the most cell types in the atlas) to a randomized background from the same number of cell types across atlas datasets (Fig. 5D). Genome-wide invariance in the mesoderm-derived cell types was at the 96th percentile of the total distribution of LAD invariance from the randomized background control, suggesting LAD/nonLAD organization is more invariant between related cell types than across more unrelated datasets. Additional data will be important for further future comparisons. Thus, we partially attribute the relatively low percent of the genome occupied by invariant LADs across the atlas to both the number and developmental diversity of the datasets being compared.

We further assessed the genes located within invariant T1- and T2-LADs. One example of a gene located in an invariant T1-LAD is *OR10K1*, an olfactory receptor gene (Fig. 5A, top right). Odorant receptor genes are highly repressed except in olfactory sensory neurons (not represented in the atlas), where multiple enhancers facilitate the expression of a single odorant receptor allele in only a few cells [50], thus the identification of this type of gene in an invariant T1-LAD followed our expectations. Genes located in invariant T1-LADs across all cell types were enriched for gene ontology (GO) biological process terms that included detection of stimulus and sensory perception (Additional file 7: Table 4). Another gene, *PAIP1*, located in an invariant T2-LAD, is a coactivator of the regulation of translation initiation (Fig. 5A, bottom right). Genes located in invariant T2-LADs were enriched for GO terms that included a broad collection of terms that did not demonstrate cell type specificity (Additional file 7: Table 4). Collectively, these data show that (1) only a minority of the regions identified as T1-LADs or T2-LADs are conserved in their LAD subtype across *all* of the cell types assayed, (2) LADs across developmentally related cell types seem to be more invariant than across random cell types, and (3) genes located within invariant LAD regions may be involved in universal functions or functions specific to a cell type absent from the atlas.

### Integrating LAD states and gene expression across different cell types

**Fig. 6:**
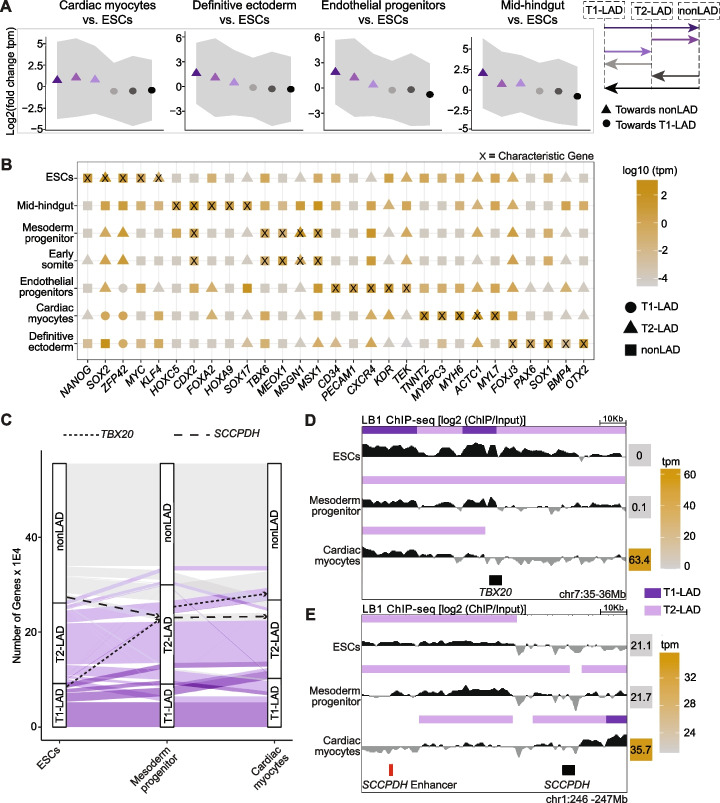
Gene expression changes broadly correspond with LAD states across developmental trajectories. **A** Fold change in gene expression of differentiated cell types relative to ESCs for each LAD category. Change indicated by arrows in the panel on the right. Points are mean and gray ribbons indicate standard deviation. ANOVA test is significant for all cell types. **B** LAD assignments and expression levels of characteristic genes for each cell type show that many canonically expressed genes are nonLADs in their respective cell types and located within LADs in alternative cell types. **C** Gene LAD assignment changes across cells from the mesoderm lineage. T1-LADs in dark purple, T2-LADs in light purple, nonLADs in gray. Genes rarely move from T1-LADs to nonLADs or vice versa, with T2-LADs showing the greatest gene occupancy gains and losses. **D,E** Track views of LB1 and LAD calls surrounding *TBX20* and *SCCPDH* and the *SCCPDH* enhancer show gene LAD occupancy changes (*TBX20*) and enhancer-LAD occupancy changes (*SCCPDH* enhancer) along a cell trajectory in the mesoderm lineage (ESCs to mesoderm progenitors to cardiac myocytes)

Because we assayed 12 cell types and found relatively few invariant LADs across all the cell types, our atlas provides an opportunity to study LAD transitions and their association with gene regulation. In line with other studies showing cell type-specific gene relocalization from LADs [12, 17, 18], we found that changes in gene expression between cell types generally corresponded to the expected change in LAD state (Fig. 6A). In several pairwise comparisons of ESCs to multiple cell types, for which we either generated matched expression data or used publically available data (see “Methods”), we found genes that moved into a LAD subtype with increased LB1 occupancy were generally downregulated, while genes which moved to a subtype with decreased LB1 occupancy were generally upregulated (Fig. 6A). In accord, we found genes that are canonical markers for various cell types are generally localized to nonLADs and highly expressed in the given cell type, although some reside in T2-LADs but are still highly expressed (Fig. 6B). Combined with the observation that these loci are not often found in T1-LADs, this analysis suggests T2-LADs may be an important transition for gene activation and expression compared to T1-LADs (Fig. 6B).

We assayed genes and their association in LADs in cell types at various levels of lineage restriction of the mesoderm lineage, including mesoderm progenitors and more differentiated mesoderm-derived cell types, such as cardiac myocytes. We observed few genes that move from nonLAD to T1-LAD (or vice versa); instead, gene association changes mostly occur between T1- and T2-LADs or between T2- and nonLADs (Fig. 6C; Additional file 2: Fig. S10B, C), providing additional support for a model in which T2-LADs are an intermediate LAD subtype that may impact spatial positioning of biologically relevant loci during lineage restriction and gene activation. For example, *TBX20*, a gene integral to cardiac development [51, 52], moves from a T1-LAD in ESCs to a T2-LAD in mesoderm progenitors and to a nonLAD in cardiac myocytes where it becomes highly expressed, suggesting the involvement of lamina-mediated genome organization in transcriptional changes during development (Fig. 6D).

Some genes did not follow the expected shift in expression upon changing LAD type. For example, *SCCPDH*, which encodes an enzyme that exhibits oxidoreductase activity [53], shows increasing LB1 occupancy across ESCs, mesoderm progenitors, and cardiac myocytes and an increase in gene expression in cardiac myocytes (Fig. 6E). It is possible that while the gene moved to a more repressive chromatin environment, its regulatory elements did not, which possibly facilitated the increased expression. We observed that a cardiac myocyte enhancer of *SCCPDH*, identified using the Activity-by-Contact model in a matched cell type [54], follows the opposite trend of its targeting gene, with decreased LB1 occupancy across the developmental trajectory, providing a potential explanation for the unexpected shift in expression of *SCCPDH* (Fig. 6E). Collectively, these results suggest that lamina-mediated organization of a gene, and potentially of its regulatory elements, may contribute to cell type-specific gene expression and could provide an additional layer of transcriptional regulation. Moreover, we observed that organizational shifts in gene location appear to occur through T2-LADs and may occur in cell types earlier in the developmental trajectory where expression is not yet changed.

## Discussion

**Fig. 7:**
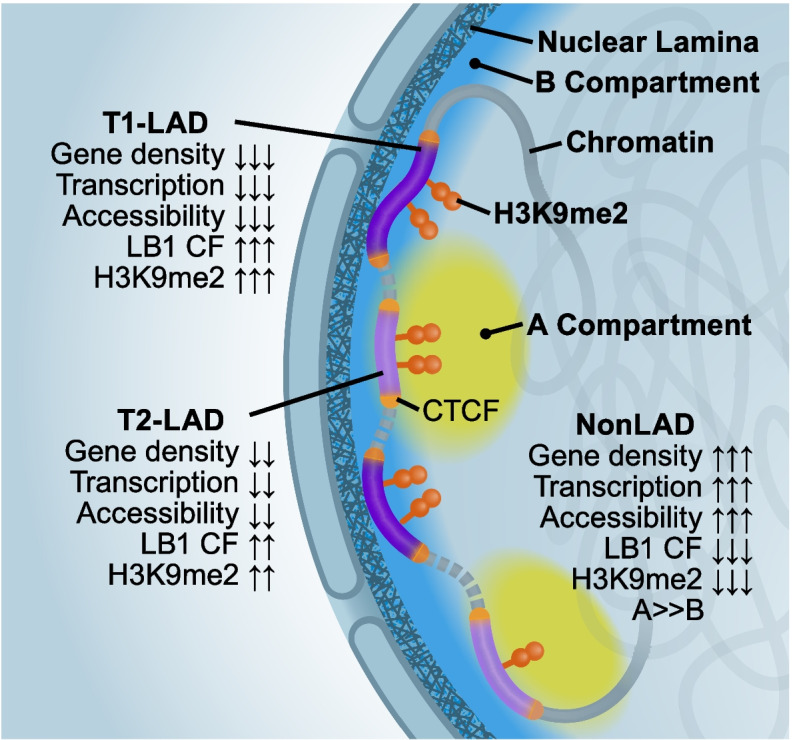
Model of T1- and T2-LADs. Data from the atlas shows T1-LADs have greater LB1 contact frequency (CF) and H3K9me2 enrichment, lower transcription, lower overall accessibility, lower gene density, and greater localization with the layer of peripheral LB1 compared to T2-LADs. This suggests that T2-LADs are a peripheral chromatin subtype localized to an intermediate layer of chromatin at the nuclear periphery that borders nonLADs

In this resource, we present genome-wide LB1 and H3K9me2 occupancy data across 12 non-immortalized human cell types, derived from common progenitors and representing multiple developmental trajectories. The breadth of the datasets allows for LAD definitions within cell types and comparisons across multiple cell types, providing additional clarity on the complex nature of lamina-associated chromatin. Work in *Drosophila* and murine cells identified subtypes of heterochromatin and/or LADs that share many characteristics with the LAD subtypes described in this work [20, 35, 55, 56], and constitutive and facultative LADs have been identified on the basis of comparing LADs and nonLADs between multiple datasets [6, 17–19, 37, 55, 57]. Our collective assessments suggest a model where distinct lamina-associated chromatin subtypes function and are maintained separately (Fig. 7).

Across all cell types analyzed in this work, we identified two different subtypes of LADs: T1- and T2-LADs. One of the most striking differences between these LAD subtypes is replication timing, where we observed an enrichment for late-replicating domains in T1-LADs, compared to a depletion in T2-LADs. Other analyses showed T1-LADs are the most inaccessible subtype, with the highest level of LB1 and H3K9me2 occupancy, strongest depletion of genes and accessible chromatin, and greatest enrichment of constitutively late-replicating chromatin. In contrast, T2-LADs show intermediate levels of LB1 and H3K9me2 binding, less gene depletion and more chromatin accessibility, and are enriched for replication timing switch domains relative to T1-LADs. As our data demonstrate, these differences between T1- and T2-LADs are likely reflections of cell-to-cell heterogeneity and the amount of the domain which is in direct contact with the lamina, raising the possibility of a more dynamic and transition-like role for the intermediate T2-LADs versus T1-LADs. Importantly, all of our genomic and imaging analyses demonstrate T1- and, more importantly, T2-LADs are enriched for LB1 and are not equivalent to nonLADs. Collectively, our results indicate that there is an intermediate peripheral chromatin subtype between T1-LADs and nonLADs.

### LAD subtypes may be established and maintained by distinct mechanisms

Our FISH results in T2-LADs suggest that one possible mechanism driving the greater accessibility of T2- versus T1-LADs is consistent intermediate positioning of T2-LAD regions between the nuclear lamina chromatin and nonLAD chromatin, rather than being embedded within the lamina like T1-LADs. Future experiments assessing the frequency of T1- versus T2-LAD association with the nuclear lamina, coupled with single-cell assessments of transcriptional activity, such as single-cell RNA-FISH or combined single-cell DamID and transcription assays [22], will greatly inform our understanding of the functional behavior that drives T1- and T2-LAD distinction, including the possibility that T1- and T2-LADs are enriched for different specific nuclear structures [48]. It is possible that there is further segregation of T2-LAD subtypes for different nuclear structures, such as repressive interior compartments. To this end, we also explored HMMs with more states, but we did not identify any biological correlates of additional LAD subtypes as striking as we observed for T2-LADs; concordance with DamID-based LADs also decreased. With additional data in the future, it may be possible to further subdivide LADs into biologically meaningful subtypes or to develop models that associate LB1 signal with function without relying on discrete subtypes.

It is important to note that we identified LAD subtypes across a diverse series of cell types, but independent assessments of key LAD subtype features show strong consistency across cell types. Future studies aimed at understanding general mechanisms required for establishing or maintaining LADs may reveal whether such mechanisms are distinct for T1- versus T2-LADs and if such differences drive localization to the lamina. Given the differences in transposable element enrichment, it will be exciting to determine if transposable elements contribute to differentiating T1- from T2-LADs. In *C. elegans*, molecular readers of H3K9 methylated chromatin are necessary to maintain localization of chromatin at the nuclear lamina [58], and manipulation of H3K9me2 results in abnormal lamina-associated chromatin organization [59]. The differing levels of H3K9me2 occupancy we observed in T1-LADs compared to T2-LADs may play an important role in the establishment of these domains. Additional studies will be essential to understand how H3K9me2 contributes both to the complexity of chromatin subtypes at the nuclear periphery and how this histone modification may be required for normal positioning and/or repositioning of lamina-associated chromatin during development.

Our studies also indicated CTCF enrichment at the borders of T1- and T2-LADs, consistent with previous studies. Recent studies indicate that the boundary element CTCF and WAPL, a protein which facilitates unloading of cohesin, are likely to be important in the positioning of LADs with specific characteristics, including LAD size and LB1 density [47, 60]. We found these characteristics differ between T1- and T2-LADs. In addition, since gene density is enriched in T2-LADs, normal positioning of a subset of T2-LADs flanked by CTCF and cohesin might be particularly sensitive to cohesin positioning factors, though additional experiments using degron-based approaches in hiPSC-derived cell types will be required to directly test this prediction.

It is also interesting to consider that T1- and T2-LADs are associated with different replication timing domains, which have previously been associated with large-scale chromatin organization [61]. These data collectively suggest that large-scale chromatin compartments are likely more complex than a binary classification. Specifically, replication timing transition regions (genomic regions between early- and late-replicating domains), demarcating permissive versus repressive chromatin, exist between topologically associated domains and show lamina association [62]. Here, we observed that T2-LADs share many of these transitional features, including higher gene density, higher basal transcription, and lower LB1 enrichment compared to T1-LADs. This suggests T2-LADs could be separated from T1-LADs by higher-order chromatin organization and play an active role in priming genes for transcription and enabling dynamic regulation of gene expression. Given the relatively higher transcription in T2-LADs, another intriguing model is that a threshold level of transcription may be required to maintain heterochromatin in T2-LADs, compared to the particularly gene-depleted and highly silenced T1-LADs [63, 64].

### LADs are linked to cell type-specific transcription and differ between cell types

Maintenance of chromatin structure via association with the nuclear periphery contributes to gene regulation and is amongst the many factors contributing to cell identity and organogenesis [6, 17, 18, 37]. Gene expression signatures are correlated to changes in LAD occupancy, providing additional support for models in which LADs are critical regulators of cellular fate. Generally, canonical genes of particular developmental cell types showed an expected nonLAD occupancy and high transcriptional signature. Across a developmental trajectory, genes that changed LAD state moved from nonLAD to T2-LAD or T2-LAD to T1-LAD, while few directly moved from nonLAD to T1-LAD (or vice versa), suggesting that the intermediate T2-LADs may be an important “priming” ground. Supporting that idea, poised promoters from chromHMM were enriched in T2-LADs. Additionally, we found examples of enhancers that gain and lose LAD occupancy in a cell type- and germline-specific manner, suggesting a role for LADs in cell type specification mediated by movement of regulatory elements towards and from the nuclear lamina.

Cell type specification and maintenance requires a complex stoichiometry of expression of many genes; perhaps the proportion of a cell population in which a locus is lamina-associated is yet another mechanism used to modulate levels of gene expression. As additional data are overlaid onto the foundational data of our atlas through future experiments, it will be exciting to identify a subset of LADs that may be linked to developmentally relevant LAD changes. For example, recent data suggest LAMIN A/C filaments can act as transcriptional repressors [65], and it is unclear if reduced lamin contact frequency of a particular locus at the nuclear periphery renders it susceptible to transcriptional activation and then relocalization, or vice versa. Our study has revealed several interesting candidates that can be used in future studies to reveal biological implications of T1- versus T2-LAD organization on cellular differentiation, perhaps in a hybrid single-cell DNA- and RNA-FISH approach with or without super resolution microscopy.

The data in this atlas represents the largest compendium of LB1 and H3K9me2 ChIP-seq across non-cancer-derived human cell types to date. ChIP-seq provided several advantages essential for this work, including avoiding genetic manipulation of ESCs to insert the Dam-fusion protein, which could impact differentiation; circumventing challenges related to the different timescales for sufficient methylation to map LADs by DamID [22, 32, 33] in cells with varied cell cycle dynamics; and allowing for assessment of H3K9me2, since a DamID fusion of a histone H3 gene would not distinguish the post-translational modification. We note that many seminal LAD studies have utilized DamID [5, 66, 67], which has distinct advantages [32], particularly with regard to mapping chromatin-binding events in single cells. Such studies will be critical to determine the probability of T1- and T2-LAD lamina occupancy within a single cell, increasing our understanding of how distinct LAD subtypes behave and are established or maintained. Atlas cell types are derived from two genetic backgrounds that were highly replicable between each other and published reference datasets, but DamID in single cells could be used in the future to define potential small-scale LB1 differences in polymorphic regions, which occur below the resolution of ChIP-seq. Ultimately, single-cell DamID data could also be coupled with the atlas data to execute targeted studies of LAD signatures and organization. For example, atlas data can be used to designate regions for super-resolution imaging, which may provide the necessary resolution to assess chromatin accessibility differences between subtypes at the nuclear periphery [68, 69], which we observe by IF-FISH as differentially localized. This could be coupled with transcriptional assessments like RNA-FISH, thereby linking population-based peripheral chromatin organization to single cells in order to understand how organization contributes to transcription or vice versa and how this impacts normal development.

## Conclusions

This work reveals critical details about peripheral chromatin organization, specifically revealing consistent segregation of peripheral chromatin within and across multiple human cell types and a cell type-specific set of overlapping but distinct lamina-associated chromatin domains. These findings lay the groundwork for future studies aimed at defining the driving cause behind the difference between T1- and T2-LADs. What is the physiologic consequence of having different subtypes of lamina-associated chromatin within a cell type? How do overall peripheral chromatin organization and LAD/KDD subtypes impact genomic and cell type stability? Is this LAD distinction conserved in other species? The data resource described herein is a foundation upon which other data can be integrated to address these and other questions in the future. These investigations promise to expand our understanding of the role genome organization plays in establishing and maintaining cellular diversity, with broad potential impacts in health and disease.

## Methods

### Generation and maintenance of cell types

#### Human pluripotent stem cell maintenance

H9 human pluripotent stem cells (WiCell) were maintained in E8 media and passaged every 4 days onto matrigel-coated plates (Roche). ESCs, cardiac myocytes, epicardium, and endothelium were H9 ESC-derived. The remainder of the cells were H7 ESC-derived. H7 pluripotent stem cells (WiCell) were maintained in feeder-free conditions using mTeSR1 media (StemCell Technologies) + 1% penicillin/streptomycin (Thermo Fisher), and fresh media was added daily. Cells were cultured on tissue culture plastics coated with Geltrex basement matrix (Thermo Fisher; which was diluted 1:100 in DMEM/F12 media [Thermo Fisher] before being used to coat culture plastics). Prior to reaching confluence, H7 ESCs were dissociated using either Accutase (Thermo Fisher) or Versene (Thermo Fisher), and then were passaged onto new plates. Both H7 (WA07) and H9 (WA09) are included in the NIH Human Embryonic Stem Cell Registry and their identity has been authenticated. The genomic identity and mycoplasma status of H7 ESCs were not assessed. H9 ESCs were tested quarterly for mycoplasma contamination.

For all H7-derived cells, differentiation was conducted in serum-free media, either Chemically Defined Medium 2 (CDM2) or Chemically Defined Medium 3 (CDM3). The composition of CDM2 basal medium [25, 26] is 50% IMDM + GlutaMAX (Thermo Fisher, 31980-097) + 50% F12 + GlutaMAX (Thermo Fisher, 31765-092) + 1 mg/mL polyvinyl alcohol (Sigma, P8136-250G) + 1% v/v chemically defined lipid concentrate (Thermo Fisher, 11905-031) + 450 μM 1-thioglycerol (Sigma, M6145-100ML) + 0.7 μg/mL recombinant human insulin (Sigma, 11376497001) + 15 μg/mL human transferrin (Sigma, 10652202001) + 1% v/v penicillin/streptomycin (Thermo Fisher, 15070-063). Polyvinyl alcohol was brought into suspension by gentle warming and magnetic stirring, and the media was sterilely filtered (through a 0.22-μm filter) prior to use.

The composition of CDM3 basal medium [23] is 45% IMDM + GlutaMAX (Thermo Fisher, 31980-097) + 45% F12 + GlutaMAX (Thermo Fisher, 31765-092) + 10% KnockOut serum replacement (Thermo Fisher, 10828028) + 1 mg/mL polyvinyl alcohol (Sigma, P8136-250G) + 1% v/v chemically defined lipid concentrate (Thermo Fisher, 11905-031) + 1% v/v penicillin/streptomycin (Thermo Fisher, 15070-063). Polyvinyl alcohol was brought into suspension by gentle warming and magnetic stirring, and the media was sterilely filtered (through a 0.22-μm filter) prior to use.

#### Cardiac myocyte differentiation

On day 0 (start of differentiation) H9 human pluripotent stem cells were treated with 1mg/ml Collagenase B (Roche) for 1 h, or until cells dissociated from plates, to generate embryoid bodies. Cells were collected and centrifuged at 300 rcf for 3 min and resuspended as small clusters of 50–100 cells by gentle pipetting in differentiation media containing RPMI (Gibco), 2 mM/L L-glutamine (Invitrogen), 4×10^4^ monothioglycerol (MTG, Sigma-Aldrich), 50 μg/ml ascorbic acid (Sigma-Aldrich). Differentiation media was supplemented with 2ng/ml BMP4 and 3 μmol Thiazovivin (Millipore). Embryoid bodies were cultured in 6-cm dishes (USA Scientific) at 37°C in 5% CO_2_, 5% O_2_, and 90% N_2_. On day 1, the media was changed to differentiation media supplemented with 30 ng/ml BMP4 (R&D Systems) and 30 ng/ml Activin A (R&D Systems), 5ng/ml bFGF (R&D Systems), and 1 μM Thiazovivin (Milipore). On day 3, embryoid bodies were harvested and washed once with DMEM (Gibco). Media was changed to differentiation media supplemented with 5 ng/ml VEGF (R&D Systems) and 5 μmol/L XAV (Stemgent). On day 5, media was changed to differentiation media supplemented with 5 ng/ml VEGF (R&D Systems). After day 8, media was changed every 3–4 days to differentiation media without supplements until approximately day 30.

#### Cardiac myocyte dissociation

Embryoid bodies were incubated overnight with 0.6mg/ml Collagenase Type II (Worthington) at 37°C. Dissociated cells were harvested and washed with Wash media (DMEM, 0.1% BSA) + 1 mg/ml DNase (VWR) twice and centrifuged at 300 rcf for 3 min. Cells were resuspended in differentiation media supplemented with 1 μM Thiazovivin (Millipore) and filtered.

#### Epicardium differentiation

The H9 human pluripotent stem cell cardiac myocyte protocol was followed up to day 3. On day 3, embryoid bodies were dissociated with TrypLE Express (Gibco). Dissociated cells were washed with Wash media (DMEM, 0.1% BSA) + 1mg/ml DNase (VWR) twice and centrifuged at 300 rcf for 3 min. Cells were resuspended in differentiation media supplemented with 1ng/ml BMP4 (R&D Systems) and filtered and counted using a hemocytometer. Cells were plated onto a matrigel-coated 96-well plate at 80,000 cells per well. On day 5, the media was changed to differentiation media supplemented with 5ng/ml VEGF (R&D Systems) and 1nM all-trans retinoic acid (Sigma-Aldrich). After day 5, media was changed every 2 days with the same day 5 differentiation media composition. On day 11, the media was changed to differentiation media supplemented with 5ng/ml VEGF (R&D Systems) and cells were fed with the same differentiation media every 2 days until day 15. On day 15, cells were dissociated with 1mg/ml Collagenase B (Roche) for 1 h, washed with Wash media (DMEM, 0.1% BSA) + 1mg/ml DNase (VWR), and centrifuged at 300 rcf for 3 min. Cells were further dissociated with 3ml TrypLE Express, washed with Wash media (DMEM, 0.1% BSA) + 1mg/ml DNase (VWR), and centrifuged at 300 rcf for 3 min. Cells were resuspended in differentiation media supplemented with 1 μM Thiazovivin (Millipore) and filtered and counted using a hemocytometer. Cells were plated in a matrigel-coated 6-well plate at 100,000 cells per well. On day 16, media was changed to differentiation media supplemented with 5ng/ml VEGF (R&D Systems) and cells were fed every 2 days until they reached confluence (approximately day 22). WT1 (WILMS-TUMOR 1) expression is indicative of successful epicardial differentiation [70].

#### Endothelial cell differentiation

The H9 human pluripotent stem cell cardiac myocyte protocol was followed up to day 5. At day 5, embryonic bodies were dissociated with TrypLE Express (Gibco). Dissociated cells were washed with Wash media (DMEM, 0.1% BSA) + 1mg/ml DNase (VWR) twice and centrifuged at 300 rcf for 3 min. Cells were resuspended in differentiation media supplemented with 100ng/ml VEGF (R&D Systems) and 50ng/ml bFGF (R&D Systems) and filtered and counted using a hemocytometer. Cells were plated onto a matrigel-coated 96-well plate at 80,000 cells per well. Media was changed every 2 days using differentiation media supplemented with 100ng/ml VEGF (R&D Systems) and 50ng/ml bFGF (R&D Systems). Cells were harvested and sorted on days 14–15.

#### Fluorescence-activated cell sorting (FACS)

Dissociated H9 human pluripotent stem cell-derived cells were resuspended in differentiation media containing diluted antibodies (dilutions listed below) for 30 min on ice. Cells were washed with differentiation media and resuspended in differentiation media + DAPI (1.35μg/ml, Biolegend) for FACS (BD FACSAria). Human pluripotent stem cell-derived cardiac myocytes used for IF-FISH were sorted by gating for SIRPA+ (PE-Cy7 anti-human CD172a/b, Biolegend, 1:200) and CD90- (APC anti-human CD90 (Thy1) Antibody, Biolegend, 1:200) cells. PSC-derived endothelial cells were sorted by gating for CD31+ (PE anti-human CD31, Biolegend, 1:200) cells.

#### Ectodermal differentiation

The day prior to beginning differentiation, H7 ESCs were dissociated with Accutase (Thermo Fisher) for 10 min at 37°C. Accutase was neutralized through the addition of excess DMEM/F12 media, and then ESCs were pelleted via centrifugation and the supernatant was aspirated. Pelleted ESCs were resuspended in mTeSR1 + 1% penicillin/streptomycin + 1 μM of the ROCK inhibitor Thiazovivin (Tocris) (henceforth referred to “cell-plating media”) and plated onto Geltrex-coated tissue culture plastics at a density of 4 × 10^5^ cells/cm^2^ (i.e., 2.1 × 10^6^ cells per 10-cm dish). Twenty-four hours after seeding, the cell-plating media was aspirated, and cells were briefly washed with DMEM/F12 to remove all traces of cell-plating media.

For definitive ectoderm induction, H7 ESCs were differentiated through the modification of a previously described method [71], in CDM2 basal media, for 24 h.

For border ectoderm induction, H7 ESCs were differentiated into *OTX2*+ definitive ectoderm in 24 h (as described above), and then definitive ectoderm was briefly washed (with DMEM/F12) and then further differentiated into *PAX3+* border ectoderm progenitors through the modification of a previously described method [42], in CDM2 basal media, for 24 h. Differentiation media was aspirated and added fresh every 24 h.

For midbrain induction, H7 ESCs were differentiated into definitive ectoderm in 24 h (as described above), and then definitive ectoderm was briefly washed (with DMEM/F12) and was further differentiated into neural progenitors through the modification of a previously described method [71], in CDM2 basal media, for 24 h. Neural progenitors were briefly washed (with DMEM/F12) and were then further differentiated into midbrain progenitors expressing *PAX2, PAX5, EN1,* and *EN2* through a modification of a previously described method [72], in CDM2 media, for 48 h. Differentiation media was aspirated and added fresh every 24 h.

#### Endodermal differentiation

The day prior to beginning differentiation, H7 ESCs were dissociated with Accutase (Thermo Fisher) at 37°C. Accutase was neutralized through the addition of excess DMEM/F12 media, and then ESCs were pelleted via centrifugation and the supernatant was aspirated. Pelleted ESCs were resuspended in cell-plating media and plated onto Geltrex-coated tissue culture plastics at a 1:8–1:16 cell seeding ratio. Twenty-four hours after seeding, the cell-plating media was aspirated, and cells were briefly washed with DMEM/F12 to remove all traces of cell-plating media.

ESCs were then differentiated into anteriormost primitive streak (not profiled) through the addition of CDM2 basal medium supplemented with Activin A (100 ng/mL; R&D Systems), CHIR99021 (3 μM; Tocris), FGF2 (20 ng/mL; R&D Systems), and PI-103 (50 nM; Tocris), which was added for 24 h. Day 1 anteriormost primitive streak cells were briefly washed (with DMEM/F12) and then differentiated into day 2 definitive endoderm through the addition of CDM2 basal medium supplemented with Activin A (100 ng/mL; R&D Systems), LDN-193189 (250 nM; Tocris), and PI-103 (50 nM; Tocris), which was added for 24 h. Methods for anteriormost primitive streak and definitive endoderm formation have been described previously [23, 25, 27].

For liver differentiation, day 2 definitive endoderm cells were briefly washed (with DMEM/F12) and further differentiated into day 3 posterior foregut through the addition of CDM3 base media supplemented with FGF2 (20 ng/mL; R&D Systems), BMP4 (30 ng/mL; R&D Systems), TTNPB (75 nM; Tocris), and A8301 (1 μM; Tocris). Day 3 posterior foregut cells were briefly washed (with DMEM/F12), and then further differentiated on days 4–6 with CDM3 base media supplemented with Activin A (10 ng/mL; R&D Systems), BMP4 (30 ng/mL; R&D Systems), and Forskolin (1 μM; Tocris) to generate liver bud progenitors expressing *HNF4A* and *TBX3*. Methods for liver bud progenitor formation have been described previously [23, 27, 73].

For mid-hindgut differentiation, day 2 definitive endoderm cells were briefly washed (with DMEM/F12) and further differentiated into day 6 mid-hindgut progenitors expressing *FOXA2*, *CDX2*, and *HOXA9* through the addition of CDM2 base media supplemented with FGF2 (100 ng/mL), BMP4 (10 ng/mL), and CHIR99021 (3 μM) for 4 days. Methods for mid-hindgut progenitor formation have been described previously [23, 25].

#### Mesodermal differentiation

The day prior to beginning differentiation, H7 ESCs were dissociated with Accutase (Thermo Fisher) at 37°C. Accutase was neutralized through the addition of excess DMEM/F12 media, and then ESCs were pelleted via centrifugation and the supernatant was aspirated. Pelleted ESCs were resuspended in cell-plating media and plated onto Geltrex-coated tissue culture plastics at a 1:8–1:16 cell seeding ratio. Twenty-four hours after seeding, the cell-plating media was aspirated, and cells were briefly washed with DMEM/F12 to remove all traces of cell-plating media.

ESCs were then sequentially differentiated into anterior primitive streak, paraxial mesoderm progenitors (“mesoderm progenitors” hereafter and in main text; enriched in *TBX6*, *CDX2*, and *MSGN1* expression), and early somites (enriched in *MEOX1* expression) as described previously [26]. Briefly, ESCs were differentiated into anterior primitive streak through the addition of CDM2 basal medium supplemented with Activin A (30 ng/mL; R&D Systems), CHIR99021 (4 μM; Tocris), FGF2 (20 ng/mL; R&D Systems), and PIK90 (100 nM; Calbiochem), which was added for 24 h, thus generating day 1 anterior primitive streak [26].

For mesoderm induction, ESCs were differentiated into anterior primitive streak in 24 h (as described above), and then anterior primitive streak was briefly washed (with DMEM/F12) and then treated with CDM2 basal media supplemented with A8301 (1 μM; Tocris), LDN193189 (250 nM; Tocris), CHIR99021 (3 μM, Tocris), and FGF2 (20 ng/mL; R&D Systems), which was added for 24 h, thus generating mesoderm progenitors [26].

For early somite induction, ESCs were differentiated into anterior primitive streak and then further differentiated into mesoderm (as described above). Mesoderm was briefly washed (with DMEM/F12) and then treated with CDM2 basal media supplemented with CDM2 base media supplemented with A8301 (1 μM; Tocris), LDN193189 (250 nM; Tocris), XAV939 (1 μM; Tocris), and PD0325901 (500 nM; Tocris) for 24 h, thus generating early somite progenitors [26].

### IF-FISH, imaging, and quantification

#### IF and IF-FISH

ESCs and cardiac myocytes were grown and/or differentiated in culture, sorted (see above), and plated for FISH by direct growth on coverslips. Cells were fixed with 4% paraformaldehyde (PFA) for 10 min at room temperature (RT) and permeabilized with 0.5% Triton X-100 for 10 min at RT. Permeabilized cells were then blocked in 1% BSA in PBS-T (8mM Na2HPO4, 150mM NaCl, 2mM KH2PO4, 3mM KCl, 0.05% Tween 20, pH 7.4) and incubated with primary and secondary antibodies for 1 h each at RT with 3 PBS-T washes for 5 min each in between antibody incubations. Primary antibodies used were anti-Lamin B1 (1:1000, Abcam #ab16048) and anti-H3K9me2 (1:1000, Active Motif #39239). Secondary antibodies used were anti-rabbit AlexaFluor 488 (1:1000, Invitrogen #21206) and anti-rabbit AlexaFluor 568 (1:1000, Invitrogen #10042).

Following IF, cells were post-fixed with 2% PFA for 10 min at RT and permeabilized with 0.7% Triton X-100 for 10 min at RT. Cells were incubated in 2× SSC-T (3.0M NaCl, 0.3M Sodium Citrate, 0.1% Tween 20) for 5 min at RT, followed by washes in 2× SSC-T with 50% formamide for 5 min at RT, 2.5 min at 92°C, and 20 min at 60°C. Cells were hybridized with a Cy2, Cy3, or Cy5 directly labeled DNA probe diluted in a hybridization mix containing 50% formamide, 1× dextran sulfate sodium salt (Fisher Scientific #BP1585) with PVSA (poly(vinylsulfonic acid, sodium salt) solution) (Sigma #278424), 10μg RNAseA, 10mM dNTPs, and 2-5pmol probe for 30 min at 80°C, then overnight (minimum 16 h) at 37°C. Probes were designed in target chromosomal regions (see Additional file 8: Table 5) using a tiled oligo-based approach with 80-mer probes spaced at 4 probes/kb in designated chromosomal regions. Cells were washed with 2× SSC-T at 60°C for 15 min followed by washes in 2× SSC-T and 2× SSC for 10 min each at RT. Cells were counterstained with DAPI solution (Sigma #D9542) diluted in 2× SSC for 5 min at RT. Cells were mounted on coverglass with SlowFade Gold antifade mounting reagent (Invitrogen #S36936) prior to image acquisition. The nonLAD probe was not dye-conjugated; for this probe, IF-FISH was performed as above with the following modifications. Primary hybridization mix contained 5μg RNAseA, 5mM dNTPs, and a probe concentration of 50pmol. Following primary probe hybridization, cells were washed with 2× SSC=T at 60°C for 15 min, followed by 2× SSC-T and 2× SSC for 10 min. A second hybridization mix was added containing 10% formamide, 1× dextran sulfate salt with PVSA, and 10pmol of a secondary probe conjugated to AlexaFluor 647. Cells were hybridized for 2 h at RT, then washed with 2× SSC-T at 60°C for 10 min, then 2× SSC-T, and 2× SSC for 5 min each. Samples were then counterstained with DAPI and mounted, as above.

#### Imaging

Confocal 3D images were taken using 120nm step Z-stacks, with an approximate range of 10–70 Z-planes per cell. Obtained images were deconvoluted using Leica Lightning Deconvolution Software. Representative images in Fig. 4 represent a single focal plane with brightness and contrast adjusted equivalently across samples in ImageJ. 3D reconstructions were performed using IMARIS v.8.1.2 software (Bitplane AG, Switzerland). Nuclear lamina and H3K9me2 surfaces were created using Surfaces tool with automatic settings based on the fluorescent signals from the anti-LB1 and anti-H3K9me2 antibodies. DNA FISH foci were generated using the Spots tool with a 500–1000-nm diameter, created at the intensity mass center of the fluorescent probe signal. Distance from the center of the FISH focus to the inner and outer edge of the nuclear lamina surface was quantified using the Measurement Points tool. Thickness of the H3K9me2-marked peripheral heterochromatin layer was calculated as the distance between the inner and outer edge of the H3K9me2 surface quantified using the Measurement Points tool with 5 random measurements per cell, from 10 independent cells per cell type (total of 50 measurements per cell type). Depth of H3K9me2 is indicated as the maximum value of those 50 measurements. In cases when the signal from a FISH focus was embedded into the nuclear lamina layer, the measurement returned negative distances. Statistical significance of the differences in localization between T1-, T2-, and nonLAD foci (Fig. 4C) was calculated by a Kruskal-Wallis test with post hoc Dunn test for multiple comparisons. Statistical significance of differences in distribution between T1-, T2-, and nonLADs was calculated by a Kolmorgov-Smirnov test (Fig. 4D). Statistical significance of localization between additional T1- and T2-LAD foci for each cell type was calculated by a Mann-Whitney test.

### ChIP, ChIP-qPCR, and ChIP-seq library preparation

#### ChIP

Undifferentiated ESCs and all differentiated cell types were crosslinked in culture by addition of methanol-free formaldehyde (Thermo Fisher, final 1% v/v) and incubated at room temperature for 10 min with gentle rotation. Crosslinking was quenched by addition of glycine (final 125mM) and incubated at room temperature for 5 min with gentle rotation. Media was discarded and replaced with PBS; cells were scraped and transferred to conical tubes and pelleted by centrifugation (250×*g*, 5 min at room temperature). Resulting pellets were flash frozen on dry ice and stored at −80°C. For ChIP, 30μL protein G magnetic beads (per ChIP sample; Dynal) were washed 3 times in blocking buffer (0.5% BSA in PBS); beads were resuspended in 250μL blocking buffer and 2μg antibody (Lamin B1: ab16048 [Abcam]; H3K9me2: ab1220 [Abcam]) and rotated at 4°C for at least 6 h. Crude nuclei were isolated from frozen crosslinked cells as follows: cell pellet (from 10cm plate) was resuspended in 10mL cold Lysis Buffer 1 (50mM HEPES-KOH pH7.5, 140mM NaCl, 1mM EDTA, 10% Glycerol, 0.5% NP-40, 0.25% Triton X-100, and protease inhibitors) and rotated at 4°C for 10 min, followed by centrifugation (250×*g*, 5 min at room temperature). Supernatant was discarded and the pellet was resuspended in 10mL cold Lysis Buffer 2 (10mM Tris-HCl pH 8.0, 200mM NaCl, 1mM EDTA, 0.5mM EGTA, and protease inhibitors) and rotated at room temperature for 10 min, followed by centrifugation (250×*g*, 5 min at room temperature). Supernatant was discarded and nuclei were resuspended/lysed in 1mL cold Lysis Buffer 3 (10mM Tris-HCl, pH 8.0, 100mM NaCl, 1mM EDTA, 0.5mM EGTA, 0.1% Na-Deoxycholate, and protease inhibitors) and transferred to pre-chilled 1-mL Covaris AFA tubes (Covaris). Samples were sonicated using a Covaris S220 sonicator (high cell chromatin shearing for 15 min; Covaris). Lysates were transferred to tubes and Triton X-100 was added (final 1%) followed by centrifugation (top speed, 10 min at 4°C in microcentrifuge). Supernatant was transferred to a new tube; protein concentration was measured by Bradford assay. Antibody-conjugated beads were washed 3 times in blocking buffer, resuspended in 50μL blocking buffer and added to 500μg input protein for overnight incubation with rotation at 4°C. Fifty micrograms lysate was aliquoted and stored at −20°C for input. On day 2, beads were washed 5 times in 1mL RIPA buffer (50mM HEPES-KOH pH 7.5, 500mM LiCl, 1mM EDTA, 1% NP-40, 0.7% Na-Deoxycholate) with 2-min incubation at room temperature with rotation for each wash. Beads were washed in 1mL final wash buffer (1×TE, 50mM NaCl) for 2 min with rotation at room temperature before final resuspension in 210μL elution buffer (50mM Tris-HCl pH 8.0, 10mM EDTA, 1% SDS). To elute, beads were incubated with agitation at 65°C for 30 min. 200μL eluate was removed to a fresh tube, and all samples (ChIP and reserved inputs) were reverse-crosslinked overnight at 65°C with agitation for a minimum of 12 h, but not more than 18 h. Two hundred microliters 1×TE was added to reverse-crosslinked DNA to dilute SDS, and samples were treated with RNaseA (final 0.2mg/mL RNase; 37°C for 2 h) and Proteinase K (final 0.2mg/mL Proteinase K; 55°C for 2 h) before phenol:chloroform extraction and resuspension in 10mM Tris-HCl pH 8.0. ChIP and input DNA was quantified by Qubit (Thermo Fisher).

#### ChIP-qPCR

Post quantification, ChIP DNA from ESCs was diluted 1:5 and used for qPCR assessment across 20 independent T1-LAD, T2-LAD, nonLAD and T1-KDD, T2-KDD, and nonKDD regions (primer sequences in Additional file 9: Table 6). qPCR was performed in 10μL reactions in 384-well format with 2μL 1:5 diluted template, 2× Power SyBr mastermix (Thermo Fisher), and 0.1μM each forward and reverse primer. qPCR reactions were run for 40 cycles using standard conditions [3 min at 95°C; 40× (15 s at 95°C; 1 min at 60°C)] on a QuantStudio 5 or QuantStudio 7 qPCR machine (Applied Biosystems). For qPCR assessments, average enrichment (average Ct ChIP/average Ct input) were quantified per primer set.

#### Library preparation

ChIP-seq libraries were prepared using the NEBNext Ultra II DNA library prep kit (NEB). Samples were indexed for multiplex sequencing. Library quality was analyzed by BioAnalyzer (Agilent Genomics) and quantified using qPCR (Kapa Biosystems or NEB). Libraries were pooled for multiplex sequencing, re-quantified, and sequenced on the Illumina NextSeq500 platform (vII; 75 bp single-end sequencing; Illumina).

#### RNA isolation and RNA-seq library preparation

Cells were scraped from tissue culture plates with 1**×**PBS and centrifuged at 1500*g* for 5 min at room temperature. After discarding supernatant, cell pellets were flash frozen in dry ice and stored at −80°C until processing. RNA was isolated using QIAGEN RNeasy total RNA extraction kit (QIAGEN). RNA quality was analyzed by BioAnalyzer; samples with RIN scores >8 were chosen for further processing. RNA libraries were prepared using the NEBNext Ultra II DNA Library Prep kit (NEB) with the NEBNext Poly(A) mRNA Magnetic Isolation Module (NEB) to enrich for poly-A-tailed RNA molecules. RNA-seq library quality was analyzed by BioAnalyzer (Agilent Genomics) and quantified using qPCR (Kapa Biosystems). Libraries were pooled for multiplex sequencing, re-quantified, and sequenced on the Illumina NextSeq500 platform (vII; 75 bp single-end sequencing; Illumina).

### ChIP-seq/RNA-seq processing and computational analyses

#### ChIP-sequencing data processing for LAMIN B1 and H3K9me2

Adapters were trimmed using Trimmomatic [v0.39] [74]. Sequencing reads were aligned to human reference hg38 using BWA-MEM [v0.7.17] [75]. Aligned reads were converted to BAM and sorted using Samtools [v0.1.19] [76], with quality filter (“-F”) set to 1804. Duplicates were removed using Picard [v2.18.7] MarkDuplicates. Sequencing reads from the ENCODE blacklist were removed using BEDTools [v2.29.0] [76, 77]. Two biological replicates were analyzed for each cell type. Track views represent one biological replicate dataset. The data for cell types based on combined replicates adhere to ENCODE3 standards (Additional file 1: Table 1) [78].

#### Identification of LADs and KDDs

LB1 and H3K9me2 ChIP-seq signals were calculated and converted into BedGraph files using deepTools bamCompare [v3.3.2] [79] with 20-kb bins, using the signal extraction scaling method [80] for sample scaling, followed by quantile normalization between cell types to decrease the impact of batch effects. The bin size of 20kb was chosen based on assessment of the literature and motivation to describe LADs in as fine of resolution as possible [19, 55, 57]. HMMs were implemented for each cell type using pomegranate [v0.11.1] [81, 82]. Each HMM was initialized using a normal distribution and *k*-means with a uniform transition matrix and trained using the Baum-Welch algorithm. Each cell type-specific model was then applied to predict LAD or KDD state genome-wide per 20-kb bin, using the median value from both replicates for each bin, for each cell type individually, filtering regions in the ENCODE blacklist from consideration. For the LAD predictions, states were labeled as T1-LAD, T2-LAD, or nonLAD based on median LB1 signal for the bins with that state label, with the highest median LB1 signal being assigned T1-LAD, second highest T2-LAD, and lowest nonLAD. The same strategy was employed to assign T1-, T2-, and nonKDDs. For validation, the HMM was repeated using single biological replicates and replicability was measured using BEDTools “intersect” command.

#### ChIP-seq analyses

LB1 occupancy at LAD boundaries was computed using the computeMatrix --referencePoint and plotProfile tools from deepTools [v3.3.2] [79]. Binned read counts for Additional file 2: Fig. S3B and Fig. S8E were generated using the deepTools “bamCoverage” tool with a minimum mapping score of 10. To determine which bins were “enriched” for LB1, the number of LB1 reads in each bin was scaled so that the total LB1 reads matched the total input reads; any bin for which the LB1 count was higher than the input count was classified as enriched. To label each bin with its LAD classification, the genome was first broken down into 10-kilobase regions that were labeled with their respective LAD classifications. Then, each bin’s starting coordinate was rounded down to the nearest 10,000; this number was used to look up the classification of that region and also assigned to the bin. In cases where neighboring bins were automatically merged by bamCompare because they had the same score, these bins were split so each was an equal size. Bins in blacklisted regions were not classified by the HMM and were excluded from these analyses. For LAD validation by overlap of narrow regions of LB1 enrichment, LB1 peaks in ESCs and CMs were called using epic2 (version 0.0.16) with paired replicate ChIP and Input bam files, and parameters “-fs 200 -bin 600 -g 4 -fdr 0.05.”

#### RNA-sequencing analysis

Transcriptome data were quantified using Kallisto [v0.44.0] quant with fragment length determined by BioAnalyzer, standard deviation of 10, and 30 bootstraps, assigning reads using the Ensembl [v96] genome annotation [83]. TPM values were quantile-normalized between cell types. Differentially expressed transcripts (*q*≤0.01) between cell types were identified using Sleuth [0.30.0] [84].

#### ATAC-seq analysis

ATAC peaks from H9-derived cells [39] were downloaded as BED files from GEO and lifted over from hg19 to hg38, taking the intersection of two replicates for each cell type.

#### CTCF analysis

Bigwig files were downloaded from GEO (see Data Access section). CrossMap was used to lift over bigwigs from hg19 to hg38.

#### Hi-C analysis

Hi-C data for CMs and ESCs were downloaded as Cooler files from the 4D Nucleome Data Portal [40]. A and B compartments were called using cooltools [v0.3.0] [85].

#### Enrichment analyses

Odds ratios were calculated based on two by two tables of counts of 20-kb genomic bins for category (T1-LAD, T2-LAD, or KDD) overlap and domain of interest (replicating timing domain, gene, transposable element, etc.) overlap. *P*-values were calculated by Fisher’s exact test.

#### Comparison with single cell DamID

Single-cell DamID data from 172 KBM-7 cells from clone 5-5 was downloaded from the Gene Expression Omnibus (GSE68260) in hg19 and lifted over using pybedtools to hg38, removing regions that did not lift over. These data were intersected with T1- and T2-LADs using pybedtools [86].

#### Comparison with DamID-seq

Human DamID-seq data from hESC cells was downloaded from the 4D Nucleome Data Portal (Data set Identifier: 4DNESNFNTUAO) [87, 88]. To evaluate LB1 enrichment, the Dam-only data (Data set Identifier: 4DNFIUYAKRND) and LB1 sequencing (Data set Identifier: 4DNFIJDN1FW4) from the same biological sample were used to create a bedgraph file using the deepTools “bamCompare” function with 10-kb bins. Each bin was then annotated with its LAD classification using a custom Python script available with the analysis code for this manuscript (see below). Overlaps in LAD calls between ChIP-seq and DamID-seq datasets were calculated using the BEDTools “intersect” command.

#### Gene ontology enrichment analyses

Enriched gene ontology terms for genes located in invariants T1- or T2-LADs was done using the HumanBase Modules tool against a background set of genes that fall in T1- or T2-LADs in at least one cell type.

#### AT content analysis

AT genomic content was calculated for either invariant T1-LADs or the whole genome using BEDTools nuc. For the latter, coordinates for invariant T1-LADs across all 12 cell types (20-kb bins) were used in a merged bed file. AT content was calculated for each invariant T1-LAD. For the whole genome, a bed file with chromosomal coordinates was used, and the median AT content was calculated across chromosomes.

#### Supporting analyses

Gene annotations used throughout are from Ensembl v96. The reference genome used was human hg38, downloaded from the UCSC Genome Browser. Constitutive late, constitutive early, and switch domains were obtained from [44]. They defined replication timing domains by their consistency across multiple cell types; thus, the same domains are used in each cell type in our analysis. Transposable elements from RepeatMasker were downloaded from the UCSC Genome Browser. Plotting, statistical analyses, and supporting analyses were conducted in Python [v3.6] with packages Jupyter, matplotlib [89], seaborn [90], upsetplot [90], scikit-learn [91], numpy [92], pybedtools [77, 86], Circos [93], and deepTools [v3.3.2] [79] and in R [v4.1.0] [94] with packages ggplot2 [95], dplyr [96], tidyverse [97], VennDiagram [98], and ggalluvial [99].

#### Violin and box plots

Boxes represent standard median (center dot or line) and interquartile range (25th to 75th percentile). Whiskers denote 1.5× interquartile range.

## Supplementary Information

**Additional file 1: Table 1.** Sequencing statistics for LAD atlas datasets. Total number of unique reads sequenced per ChIP condition (LB1, H3K9me2 or Input) per replicate in all atlas cell types.

**Additional file 2: Fig. S1.** Validation of ESC-derived cell types. Differentiation validation by qPCR of key pluripotency genes, schematics of cell specification, cell sorting. **Fig. S2.** LB1 antibody ChIP and specificity validation; 3-state HMM validations. LB1 ChIP-immunoblot, AIC/BIC of LAD states, LAD sizes. **Fig. S3.** Validation of LADs identified by a 3-state HMM. Validation of LB1 enrichment in LADs, GC content in LADs. **Fig. S4.** LAD transition state parameters. HMM transition parameters from each cell type-specific model (log2-transformed for visualization purposes) for LADs. **Fig. S5.** H3K9me2 antibody ChIP and specificity validation; 3-state KDD validations. H3K9me2 ChIP-immunoblot and dot blot, AIC/BIC of KDD states. **Fig. S6.** H3K9me2 HMM validation. Validation of H3K9me2 enrichment in KDDs, KDD sizes. **Fig. S7.** KDD transition state parameters. HMM transition parameters from each cell type-specific model (log2-transformed for visualization purposes) for KDDs. **Fig. S8.** KDDs and LADs are highly overlapping; T2-LADs overlap vLADs. LAD/KDD overlap in track view, comparison of ESC ChIP-seq LADs to DamID LADs from 9 immortalized cell lines. **Fig. S9.** IF-FISH probes, H3K9me2 IF and cell sorting for IF-FISH. Median LB1 signal per FISH probe for ESCs and CMs, quantification of H3K9me2 enrichment at the nuclear periphery, track view of probes, additional FISH data. **Fig. S10.** Genes change LAD assignments between cell types. AT enrichment in invariant LADs, a subset of gene changes across developmentally linked cell types.

**Additional file 3: Video 1.** Cardiac cells beating in culture.

**Additional file 4: Video 2.** Cardiac cells beating in culture.

**Additional file 5: Table 2.** HMM-identified LADs in the LAD atlas. T1- and T2-LADs for each cell type in the atlas in .bed format. Cell types separated by worksheets.

**Additional file 6: Table 3.** HMM-identified KDDs in the LAD atlas. T1- and T2-KDDs for each cell type in the atlas in .bed format. Cell types separated by worksheets.

**Additional file 7: Table 4.** GO categories for invariant T1- and T2-LADs. Enriched gene ontology terms for genes located in invariant T1- or T2-LADs.

**Additional file 8: Table 5.** FISH probes locations. Chromosomal regions used to design FISH probes provided.

**Additional file 9: Table 6.** qPCR primer sequences used for LB1 and H3K9me2 HMM validation. Sequences provided for the 20 T1-, T2- and nonLAD regions assessed for LB1 enrichment by ChIP-qPCR.

**Additional file 10.** Review history.

## Data Availability

*Data generated in this study*: RNA-seq data from ventricular CMs, ESCs and endothelial cells, ChIP-seq data for LB1 and H3K9me2 from all cell types, LAD and KDD calls are available through the Gene Expression Omnibus (GSE155244) [100]. *Code Availability*: Supporting code for this paper is available via Github [101] and via Zenodo [102]. *RNA-seq*: Expression data from day 3 early somite and mesoderm progenitor cells were downloaded from SRP073808 [41, 103]. Data from mid-hindgut were downloaded from SRP033267 [25, 104]. Data from neural ectoderm were downloaded from SRP113027 [42, 105]. *ATAC-seq*: Data from ESCs, CMs and day 2 mesoderm were downloaded from GSE85330 [39, 106]. *CTCF ChIP-seq*: Data from ESCs were downloaded from GSM3263064 [107] and GSM3263065 [108], from day 2 mesoderm from GSM3263066 [109] and GSM3263067 [110] and from day 80 ventricular CMs from GSM3263074 [40, 111]. *Hi-C*: Data from ESCs were downloaded from GSM3263064 [112] and GSM3263065 [108] and from day 80 ventricular CMs from GSM3263074 [40, 111]. *Transcription factor binding sites*: Transcription factor clustered binding sites from the ENCODE project were downloaded from the UCSC Genome Browser [78, 113]. *chromHMM states*: chromHMM states in reference genome hg38 from the 25-state model were downloaded for cell types E008, E095, E066, and E013 as the closest cell types matching ESCs, cardiac myocytes, liver cells, and mesoderm progenitor cells from Chromatin state model based on imputed data (25 state, 12 marks, 127 epigenomes) [114]. *4DN Consortium DamID Tier1 hESC Data*: DamID LB1 enrichment and associated LADs from the van Steensel lab were downloaded from the 4DN Data Portal (Data set Identifier: 4DNESNFNTUAO), https://data.4dnucleome.org/experiment-sets/4DNESNFNTUAO/ [115].
