# Supplementary material for: An atlas of lamina-associated chromatin across twelve human cell types reveals an intermediate chromatin subtype
Source: Genome Biol. 2023 Jan 23;24:16. doi: 10.1186/s13059-023-02849-5 (PMC9869549; doi:10.1186/s13059-023-02849-5)
Supplement: Supplementary file 2 — Additional file 2: Fig. S1. Validation of ESC-derived cell types. Differentiation validation by qPCR of key pluripotency genes, schematics of cell specification, cell sorting. Fig. S2. LB1 antibody ChIP and specificity validation; 3-state HMM validations. LB1 ChIP-immunoblot, AIC/BIC of LAD states, LAD sizes. Fig. S3. Validation of LADs identified by a 3-state HMM. Validation of LB1 enrichment in LADs, GC content in LADs. Fig. S4. LAD transition state parameters. HMM transition parameters from each cell type-specific model (log2-transformed for visualization purposes) for LADs. Fig. S5. H3K9me2 antibody ChIP and specificity validation; 3-state KDD validations. H3K9me2 ChIP-immunoblot and dot blot, AIC/BIC of KDD states. Fig. S6. H3K9me2 HMM validation. Validation of H3K9me2 enrichment in KDDs, KDD sizes. Fig. S7. KDD transition state parameters. HMM transition parameters from each cell type-specific model (log2-transformed for visualization purposes) for KDDs. Fig. S8. KDDs and LADs are highly overlapping; T2-LADs overlap vLADs. LAD/KDD overlap in track view, comparison of ESC ChIP-seq LADs to DamID LADs from 9 immortalized cell lines. Fig. S9. IF-FISH probes, H3K9me2 IF and cell sorting for IF-FISH. Median LB1 signal per FISH probe for ESCs and CMs, quantification of H3K9me2 enrichment at the nuclear periphery, track view of probes, additional FISH data. Fig. S10. Genes change LAD assignments between cell types. AT enrichment in invariant LADs, a subset of gene changes across developmentally linked cell types. [file 13059_2023_2849_MOESM2_ESM.pdf]

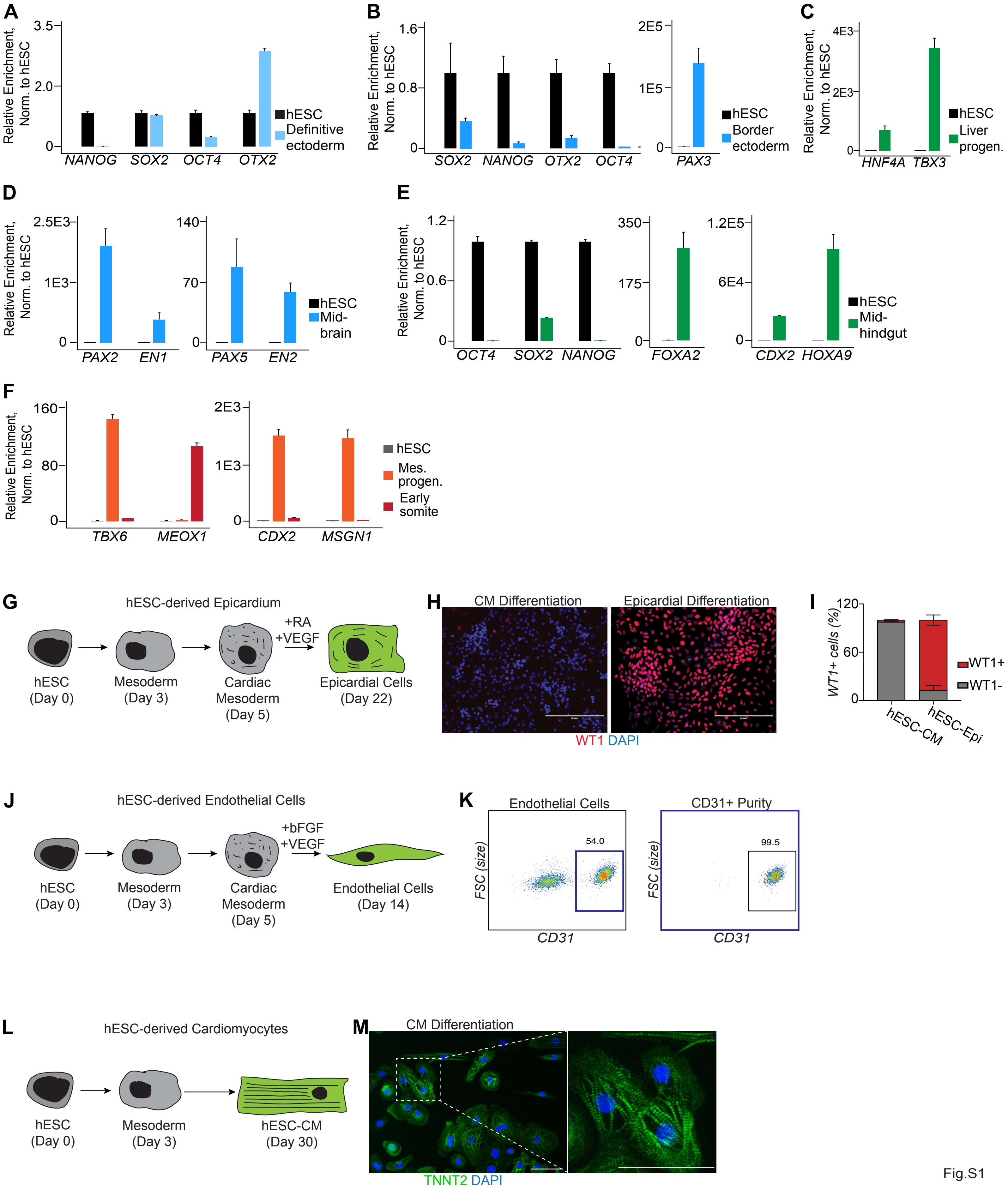

A
Relative Enrichment, Norm. to hESC
3.5
2.0
0
NANOG SOX2 OCT4 OTX2
hESC
Definitive ectoderm
B
Relative Enrichment, Norm. to hESC
1.6
0.8
0
SOX2 NANOG OTX2 OCT4
2E5
1E5
0
PAX3
hESC
Border ectoderm
C
Relative Enrichment, Norm. to hESC
4E3
2E3
0
HNF4A TBX3
hESC
Liver progen.
D
Relative Enrichment, Norm. to hESC
2.5E3
1E3
0
PAX2 EN1
140
70
0
PAX5 EN2
hESC
Mid-brain
E
Relative Enrichment, Norm. to hESC
1.2
0.6
0
OCT4 SOX2 NANOG
350
175
0
FOXA2
1.2E5
6E4
0
CDX2 HOXA9
hESC
Mid-hindgut
F
Relative Enrichment, Norm. to hESC
160
80
0
TBX6 MEOX1
2E3
1E3
0
CDX2 MSGN1
hESC
Mes. progen.
Early somite
G
hESC-derived Epicardium
+RA
+VEGF
hESC (Day 0)
Mesoderm (Day 3)
Cardiac Mesoderm (Day 5)
Epicardial Cells (Day 22)
H
CM Differentiation
Epicardial Differentiation
WT1 DAPI
I
WT1+ cells (%)
120
60
0
WT1+
WT1-
hESC-CM
hESC-Epi
J
hESC-derived Endothelial Cells
+bFGF
+VEGF
hESC (Day 0)
Mesoderm (Day 3)
Cardiac Mesoderm (Day 5)
Endothelial Cells (Day 14)
K
Endothelial Cells
54.0
FSC (size)
CD31
CD31+ Purity
99.5
FSC (size)
CD31
L
hESC-derived Cardiomyocytes
hESC (Day 0)
Mesoderm (Day 3)
hESC-CM (Day 30)
M
CM Differentiation
TNNT2 DAPI

Fig.S1

Fig. S2

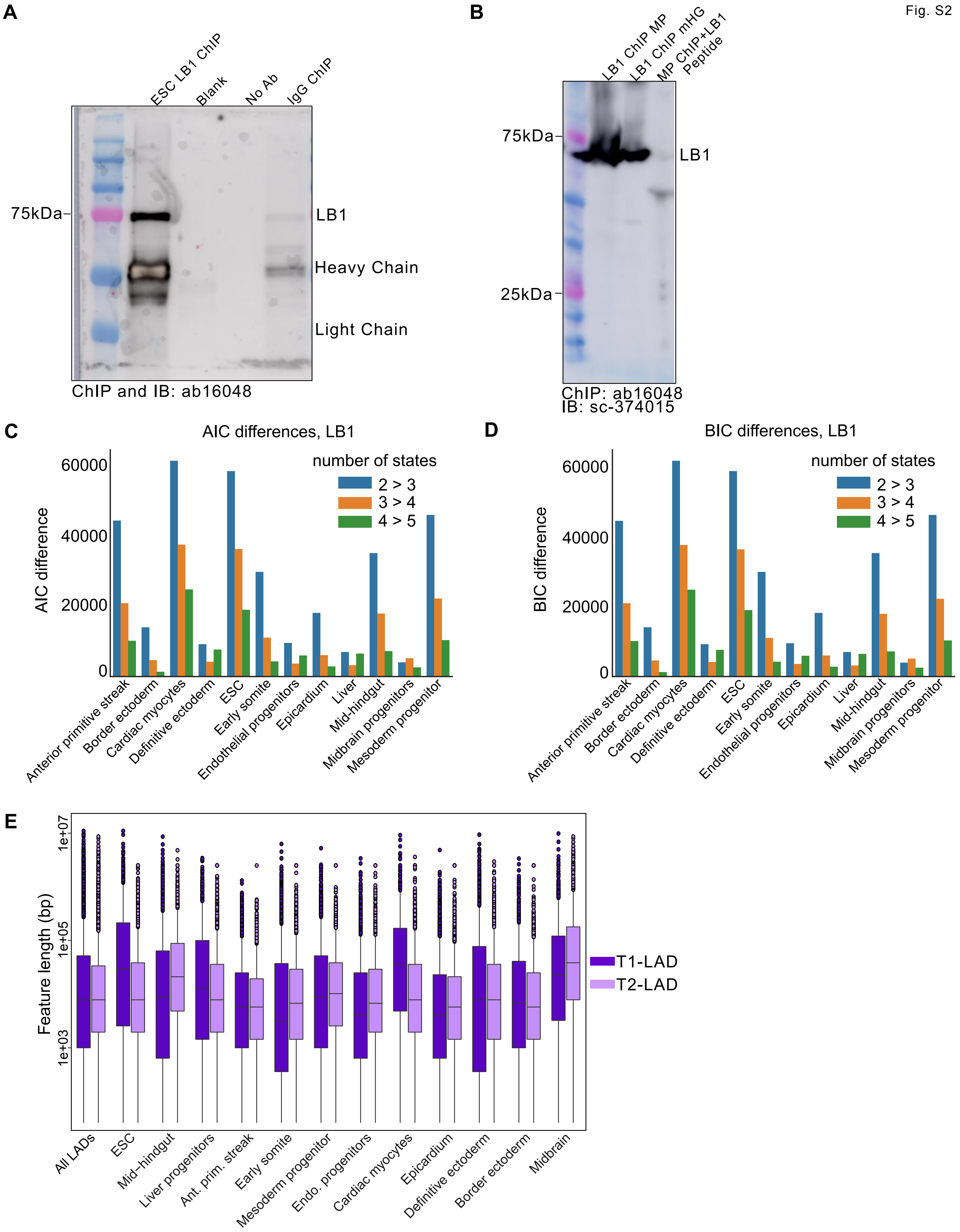
A
ESC LB1 ChIP
Blank
No Ab
IgG ChIP
75kDa
LB1
Heavy Chain
Light Chain
ChIP and IB: ab16048
B
LB1 ChIP MP
LB1 ChIP mHG
MP ChIP+LB1 Peptide
75kDa
25kDa
LB1
ChIP: ab16048
IB: sc-374015
C
AIC differences, LB1
AIC difference
60000
40000
20000
0
number of states
2 > 3
3 > 4
4 > 5
Anterior primitive streak
Border ectoderm
Cardiac myocytes
Definitive ectoderm
ESC
Early somite
Endothelial progenitors
Epicardium
Liver
Mid-hindgut
Midbrain progenitors
Mesoderm progenitor
D
BIC differences, LB1
BIC difference
60000
40000
20000
0
number of states
2 > 3
3 > 4
4 > 5
Anterior primitive streak
Border ectoderm
Cardiac myocytes
Definitive ectoderm
ESC
Early somite
Endothelial progenitors
Epicardium
Liver
Mid-hindgut
Midbrain progenitors
Mesoderm progenitor
E
Feature length (bp)
1e+07
1e+05
1e+03
T1-LAD
T2-LAD
All LADs
ESC
Mid-hindgut
Liver progenitors
Ant. prim. streak
Early somite
Mesoderm progenitor
Endo. progenitors
Cardiac myocytes
Epicardium
Definitive ectoderm
Border ectoderm
Midbrain

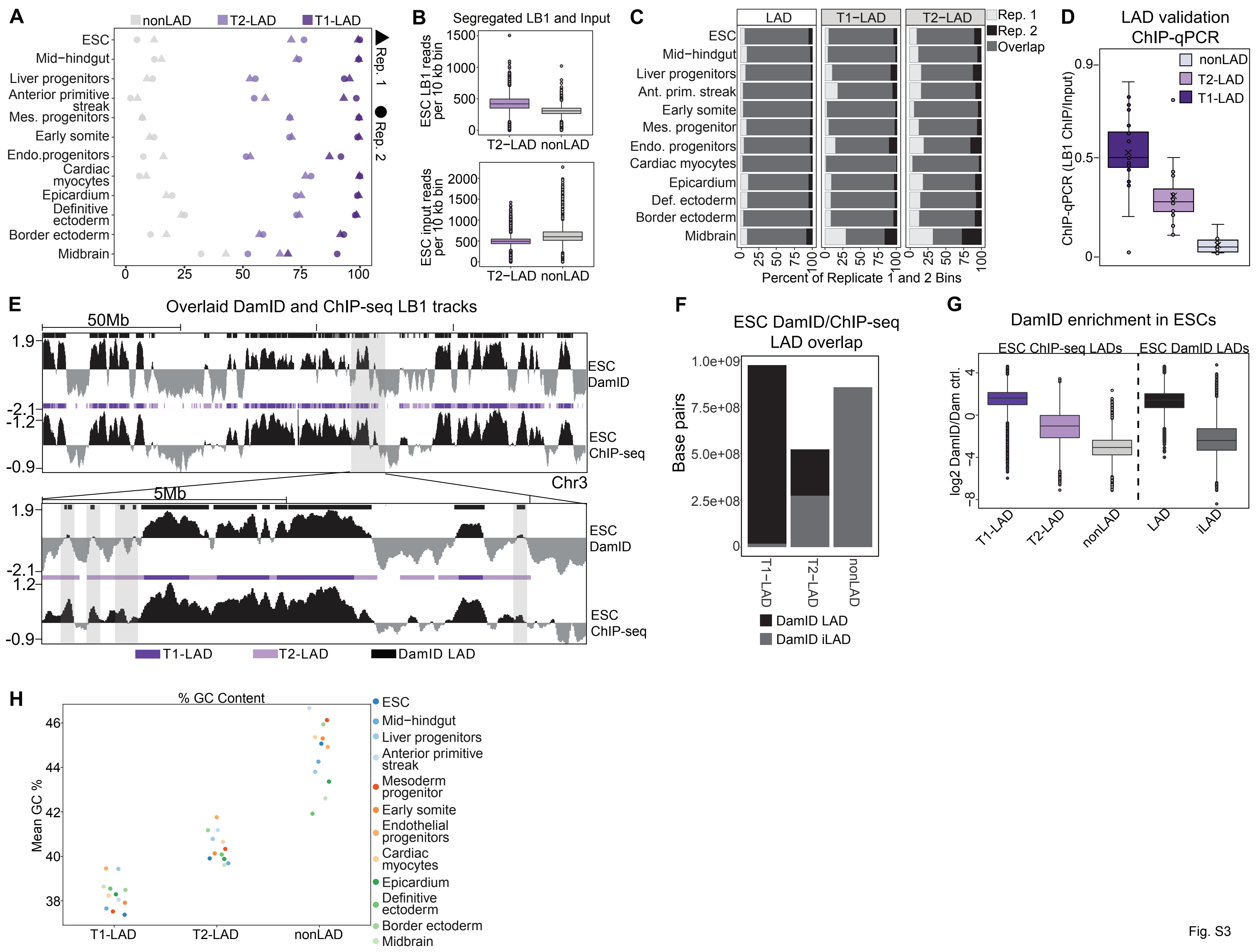

A
nonLAD
T2-LAD
T1-LAD
Rep. 1
Rep. 2
ESC
Mid−hindgut
Liver progenitors
Anterior primitive streak
Mes. progenitors
Early somite
Endo.progenitors
Cardiac myocytes
Epicardium
Definitive ectoderm
Border ectoderm
Midbrain
B
Segregated LB1 and Input
ESC LB1 reads per 10 kb bin
ESC input reads per 10 kb bin
T2−LAD nonLAD
C
LAD
T1-LAD
T2-LAD
Rep. 1
Rep. 2
Overlap
ESC
Mid−hindgut
Liver progenitors
Ant. prim. streak
Early somite
Mes. progenitor
Endo. progenitors
Cardiac myocytes
Epicardium
Def. ectoderm
Border ectoderm
Midbrain
Percent of Replicate 1 and 2 Bins
D
LAD validation ChIP-qPCR
ChIP-qPCR (LB1 ChIP/Input)
nonLAD
T2-LAD
T1-LAD
E
Overlaid DamID and ChIP-seq LB1 tracks
50Mb
5Mb
ESC DamID
ESC ChIP-seq
Chr3
T1-LAD
T2-LAD
DamID LAD
F
ESC DamID/ChIP-seq LAD overlap
Base pairs
T1−LAD
T2−LAD
nonLAD
DamID LAD
DamID iLAD
G
DamID enrichment in ESCs
ESC ChIP-seq LADs
ESC DamID LADs
log2 DamID/Dam ctrl.
T1-LAD
T2-LAD
nonLAD
LAD
iLAD
H
% GC Content
Mean GC %
T1-LAD
T2-LAD
nonLAD
ESC
Mid−hindgut
Liver progenitors
Anterior primitive streak
Mesoderm progenitor
Early somite
Endothelial progenitors
Cardiac myocytes
Epicardium
Definitive ectoderm
Border ectoderm
Midbrain

Fig. S3

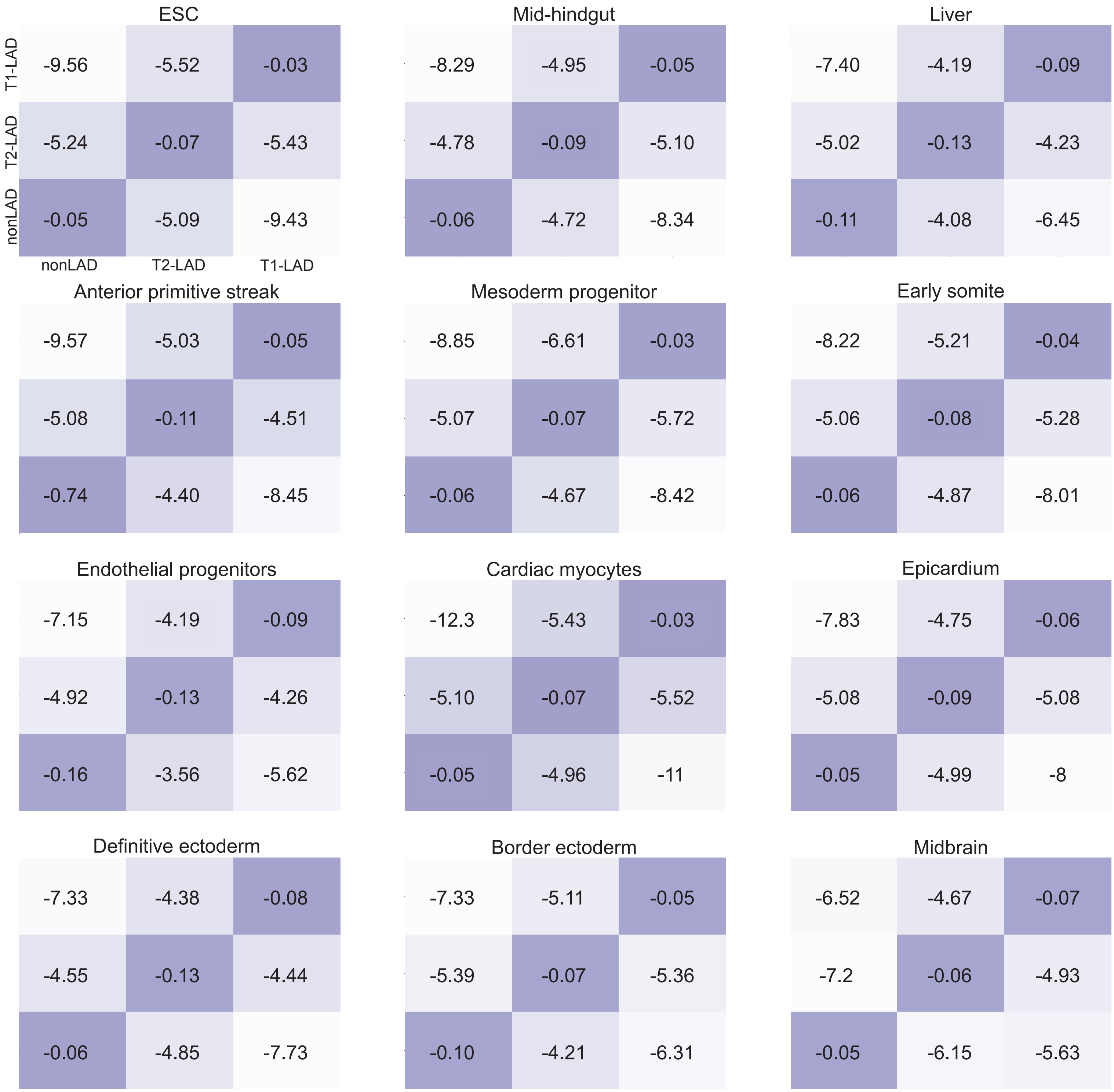
ESC
T1-LAD
-9.56 -5.52 -0.03
T2-LAD
-5.24 -0.07 -5.43
nonLAD
-0.05 -5.09 -9.43
nonLAD
T2-LAD
T1-LAD
Mid-hindgut
-8.29 -4.95 -0.05
-4.78 -0.09 -5.10
-0.06 -4.72 -8.34
Liver
-7.40 -4.19 -0.09
-5.02 -0.13 -4.23
-0.11 -4.08 -6.45
Anterior primitive streak
-9.57 -5.03 -0.05
-5.08 -0.11 -4.51
-0.74 -4.40 -8.45
Mesoderm progenitor
-8.85 -6.61 -0.03
-5.07 -0.07 -5.72
-0.06 -4.67 -8.42
Early somite
-8.22 -5.21 -0.04
-5.06 -0.08 -5.28
-0.06 -4.87 -8.01
Endothelial progenitors
-7.15 -4.19 -0.09
-4.92 -0.13 -4.26
-0.16 -3.56 -5.62
Cardiac myocytes
-12.3 -5.43 -0.03
-5.10 -0.07 -5.52
-0.05 -4.96 -11
Epicardium
-7.83 -4.75 -0.06
-5.08 -0.09 -5.08
-0.05 -4.99 -8
Definitive ectoderm
-7.33 -4.38 -0.08
-4.55 -0.13 -4.44
-0.06 -4.85 -7.73
Border ectoderm
-7.33 -5.11 -0.05
-5.39 -0.07 -5.36
-0.10 -4.21 -6.31
Midbrain
-6.52 -4.67 -0.07
-7.2 -0.06 -4.93
-0.05 -6.15 -5.63

Fig. S4

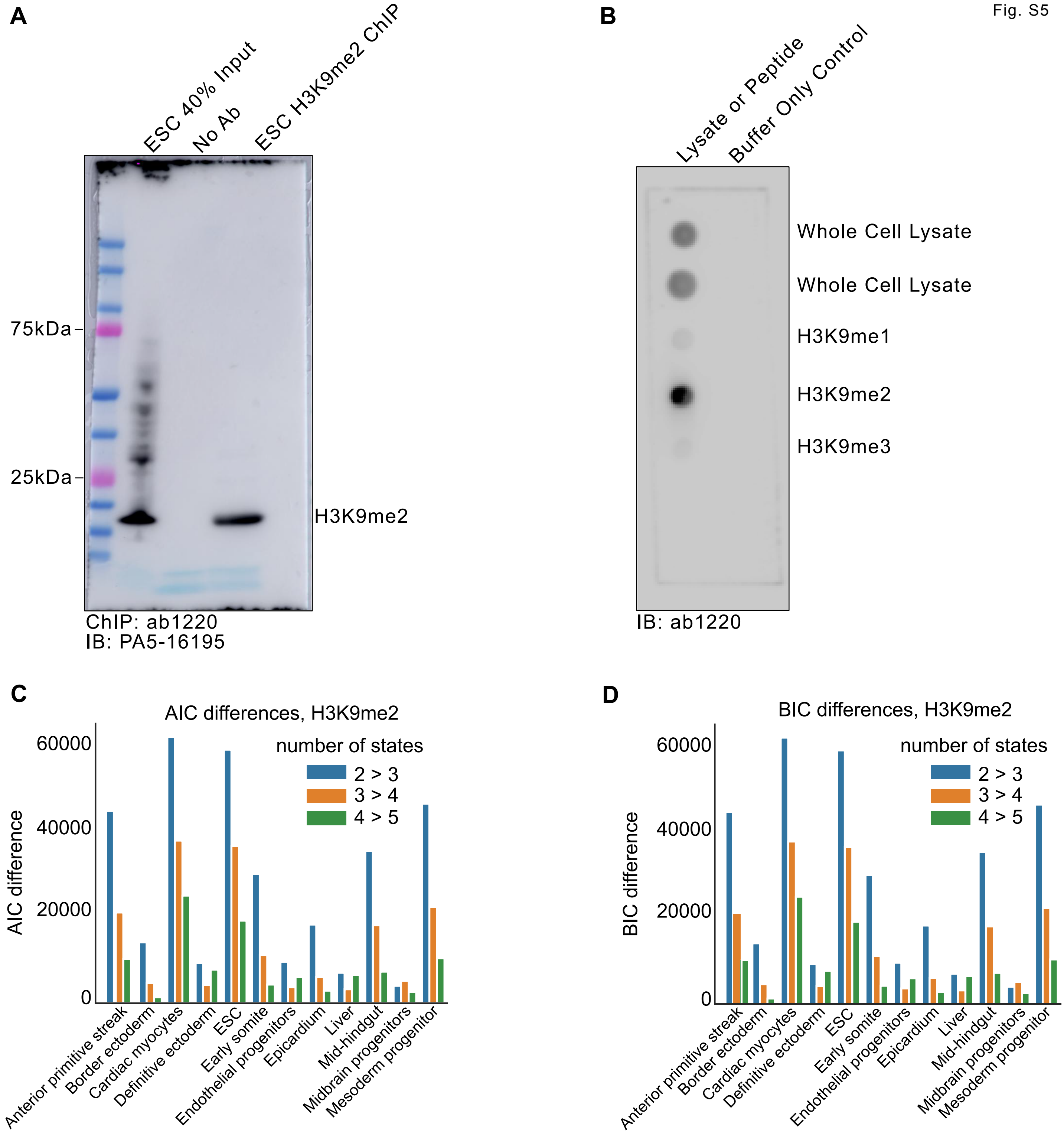
A
ESC 40% Input
No Ab
ESC H3K9me2 ChIP
75kDa
25kDa
H3K9me2
ChIP: ab1220
IB: PA5-16195
B
Lysate or Peptide
Buffer Only Control
Whole Cell Lysate
Whole Cell Lysate
H3K9me1
H3K9me2
H3K9me3
IB: ab1220
C
AIC differences, H3K9me2
AIC difference
60000
40000
20000
0
number of states
2 > 3
3 > 4
4 > 5
Anterior primitive streak
Border ectoderm
Cardiac myocytes
Definitive ectoderm
ESC
Early somite
Endothelial progenitors
Epicardium
Liver
Mid-hindgut
Midbrain progenitors
Mesoderm progenitor
D
BIC differences, H3K9me2
BIC difference
60000
40000
20000
0
number of states
2 > 3
3 > 4
4 > 5
Anterior primitive streak
Border ectoderm
Cardiac myocytes
Definitive ectoderm
ESC
Early somite
Endothelial progenitors
Epicardium
Liver
Mid-hindgut
Midbrain progenitors
Mesoderm progenitor

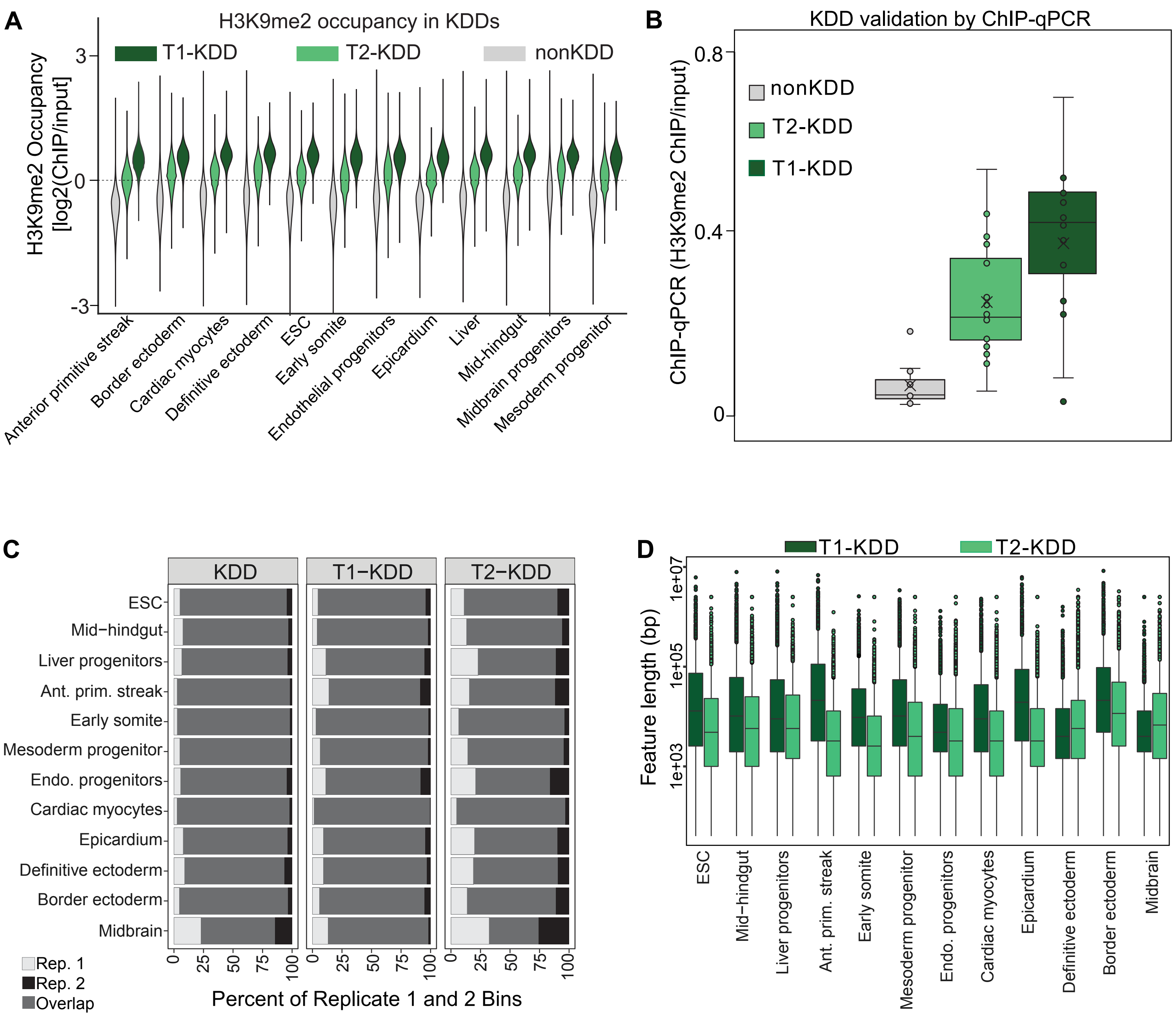
A
H3K9me2 occupancy in KDDs
T1-KDD
T2-KDD
nonKDD
H3K9me2 Occupancy [log2(ChIP/input]
3
0
-3
Anterior primitive streak
Border ectoderm
Cardiac myocytes
Definitive ectoderm
ESC
Early somite
Endothelial progenitors
Epicardium
Liver
Mid-hindgut
Midbrain progenitors
Mesoderm progenitor
B
KDD validation by ChIP-qPCR
nonKDD
T2-KDD
T1-KDD
ChIP-qPCR (H3K9me2 ChIP/input)
0.8
0.4
0
C
KDD
T1−KDD
T2−KDD
ESC
Mid−hindgut
Liver progenitors
Ant. prim. streak
Early somite
Mesoderm progenitor
Endo. progenitors
Cardiac myocytes
Epicardium
Definitive ectoderm
Border ectoderm
Midbrain
0
25
50
75
100
Rep. 1
Rep. 2
Overlap
Percent of Replicate 1 and 2 Bins
D
T1-KDD
T2-KDD
Feature length (bp)
1e+07
1e+05
1e+03
ESC
Mid−hindgut
Liver progenitors
Ant. prim. streak
Early somite
Mesoderm progenitor
Endo. progenitors
Cardiac myocytes
Epicardium
Definitive ectoderm
Border ectoderm
Midbrain

Fig. S6

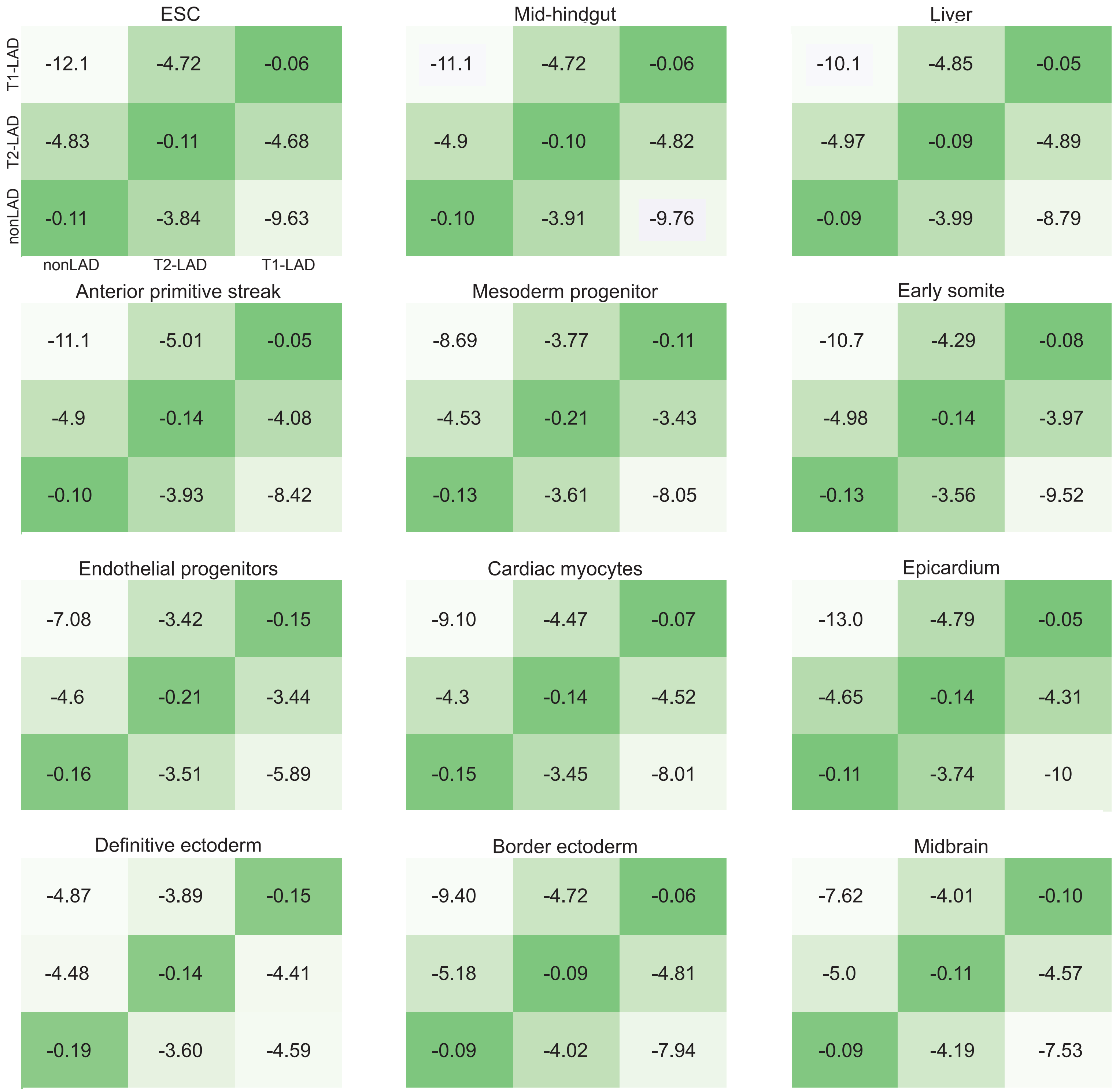
ESC
T1-LAD
T2-LAD
nonLAD
-12.1 -4.72 -0.06
-4.83 -0.11 -4.68
-0.11 -3.84 -9.63
nonLAD
T2-LAD
T1-LAD
Mid-hindgut
-11.1 -4.72 -0.06
-4.9 -0.10 -4.82
-0.10 -3.91 -9.76
Liver
-10.1 -4.85 -0.05
-4.97 -0.09 -4.89
-0.09 -3.99 -8.79
Anterior primitive streak
-11.1 -5.01 -0.05
-4.9 -0.14 -4.08
-0.10 -3.93 -8.42
Mesoderm progenitor
-8.69 -3.77 -0.11
-4.53 -0.21 -3.43
-0.13 -3.61 -8.05
Early somite
-10.7 -4.29 -0.08
-4.98 -0.14 -3.97
-0.13 -3.56 -9.52
Endothelial progenitors
-7.08 -3.42 -0.15
-4.6 -0.21 -3.44
-0.16 -3.51 -5.89
Cardiac myocytes
-9.10 -4.47 -0.07
-4.3 -0.14 -4.52
-0.15 -3.45 -8.01
Epicardium
-13.0 -4.79 -0.05
-4.65 -0.14 -4.31
-0.11 -3.74 -10
Definitive ectoderm
-4.87 -3.89 -0.15
-4.48 -0.14 -4.41
-0.19 -3.60 -4.59
Border ectoderm
-9.40 -4.72 -0.06
-5.18 -0.09 -4.81
-0.09 -4.02 -7.94
Midbrain
-7.62 -4.01 -0.10
-5.0 -0.11 -4.57
-0.09 -4.19 -7.53

Fig. S7

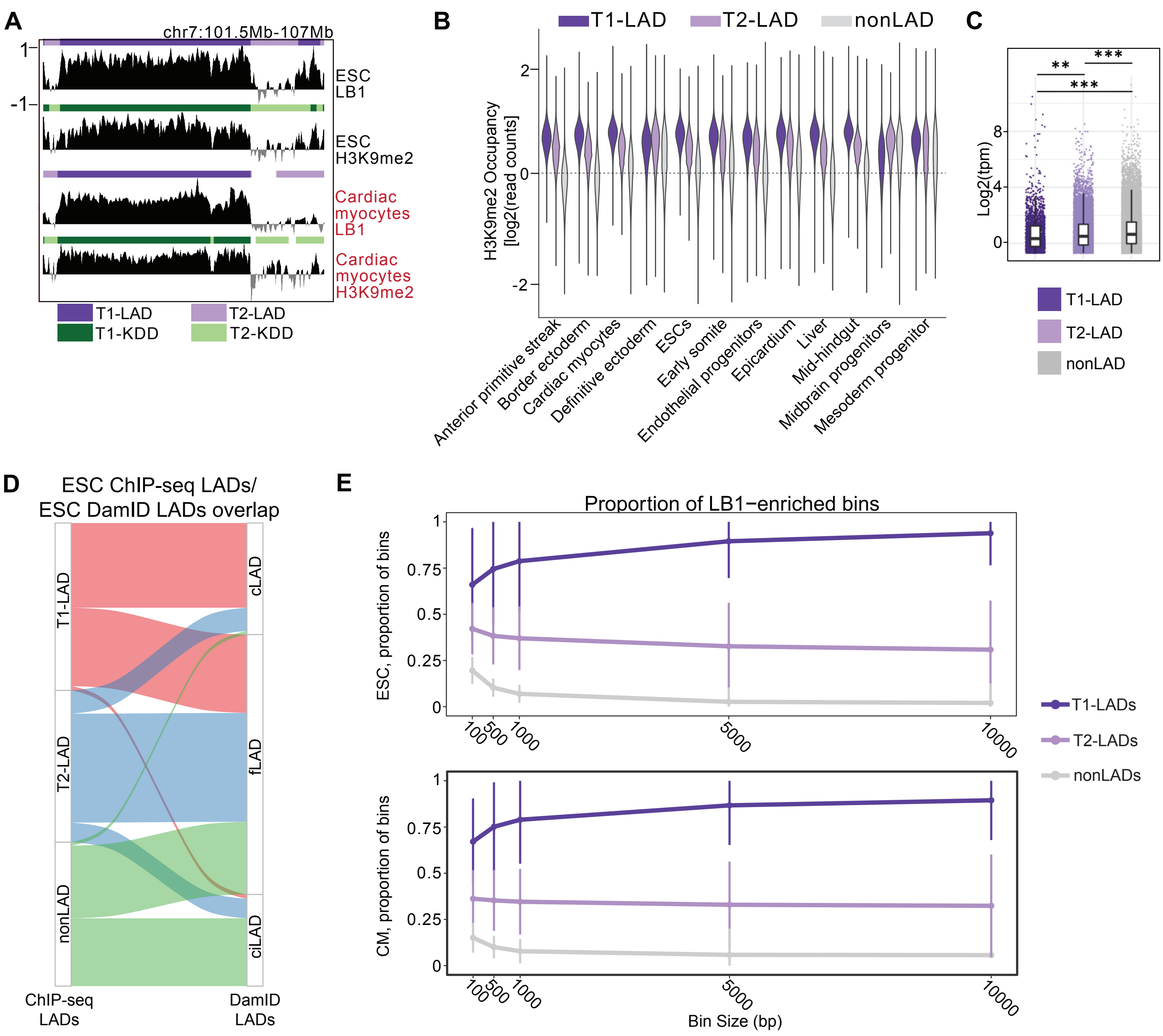
A
chr7:101.5Mb-107Mb
1
-1
ESC LB1
ESC H3K9me2
Cardiac myocytes LB1
Cardiac myocytes H3K9me2
T1-LAD
T2-LAD
T1-KDD
T2-KDD
B
T1-LAD
T2-LAD
nonLAD
H3K9me2 Occupancy [log2(read counts]
2
0
-2
Anterior primitive streak
Border ectoderm
Cardiac myocytes
Definitive ectoderm
ESCs
Early somite
Endothelial progenitors
Epicardium
Liver
Mid-hindgut
Midbrain progenitors
Mesoderm progenitor
C
**
***
***
Log2(tpm)
8
4
0
T1-LAD
T2-LAD
nonLAD
D
ESC ChIP-seq LADs/ ESC DamID LADs overlap
T1-LAD
T2-LAD
nonLAD
cLAD
fLAD
ciLAD
ChIP-seq LADs
DamID LADs
E
Proportion of LB1−enriched bins
ESC, proportion of bins
CM, proportion of bins
1
0.75
0.5
0.25
0
100
500
1000
5000
10000
Bin Size (bp)
T1-LADs
T2-LADs
nonLADs

Fig. S8

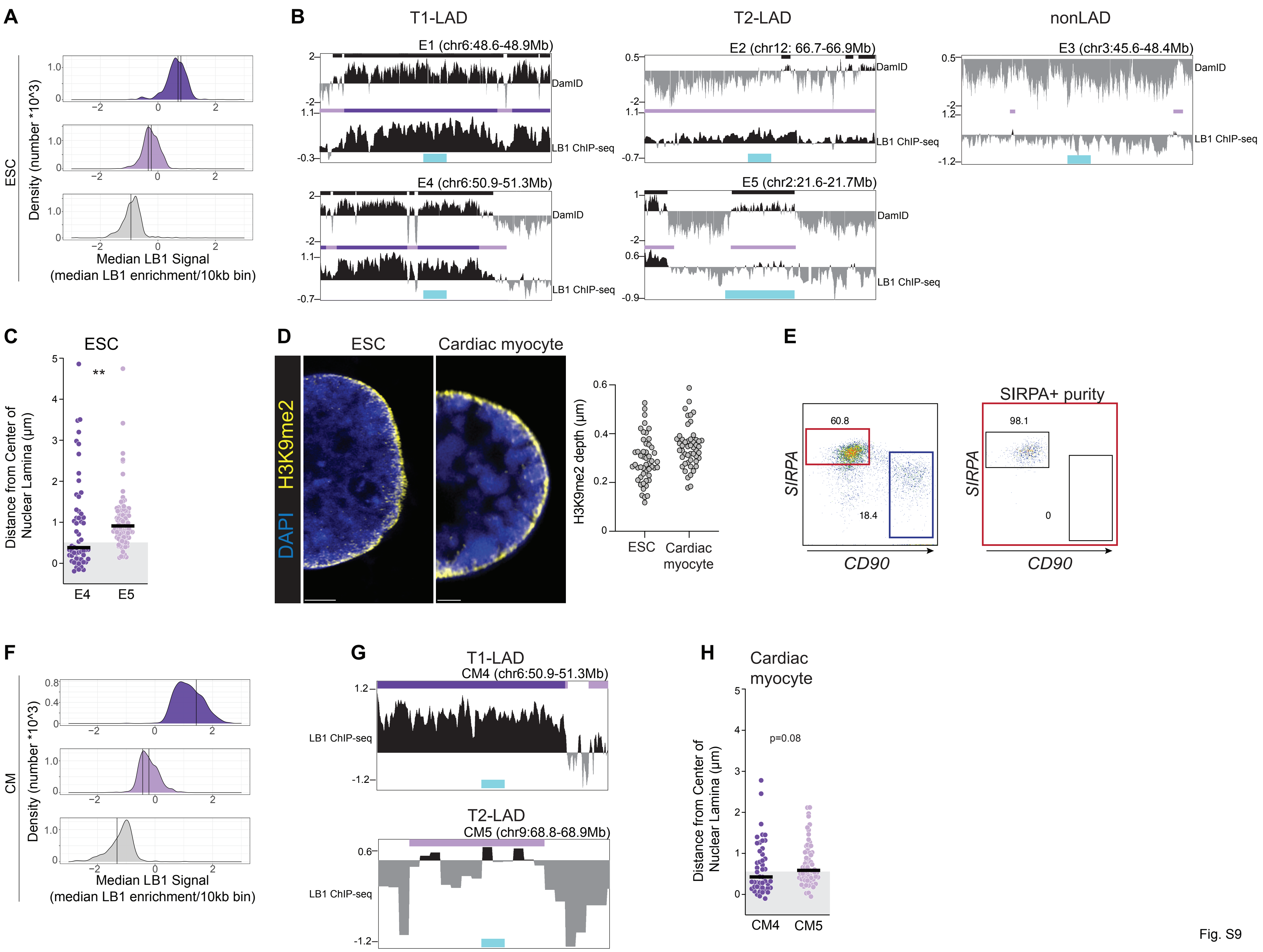
A
ESC
Density (number *10^3)
Median LB1 Signal
(median LB1 enrichment/10kb bin)
B
T1-LAD
T2-LAD
nonLAD
E1 (chr6:48.6-48.9Mb)
E2 (chr12: 66.7-66.9Mb)
E3 (chr3:45.6-48.4Mb)
E4 (chr6:50.9-51.3Mb)
E5 (chr2:21.6-21.7Mb)
DamID
LB1 ChIP-seq
C
ESC
Distance from Center of
Nuclear Lamina (μm)
E4
E5
D
ESC
Cardiac myocyte
DAPI
H3K9me2
H3K9me2 depth (μm)
Cardiac
myocyte
E
SIRPA+ purity
60.8
18.4
98.1
0
SIRPA
CD90
F
CM
G
T1-LAD
CM4 (chr6:50.9-51.3Mb)
T2-LAD
CM5 (chr9:68.8-68.9Mb)
H
Cardiac
myocyte
p=0.08
CM4
CM5

Fig. S9

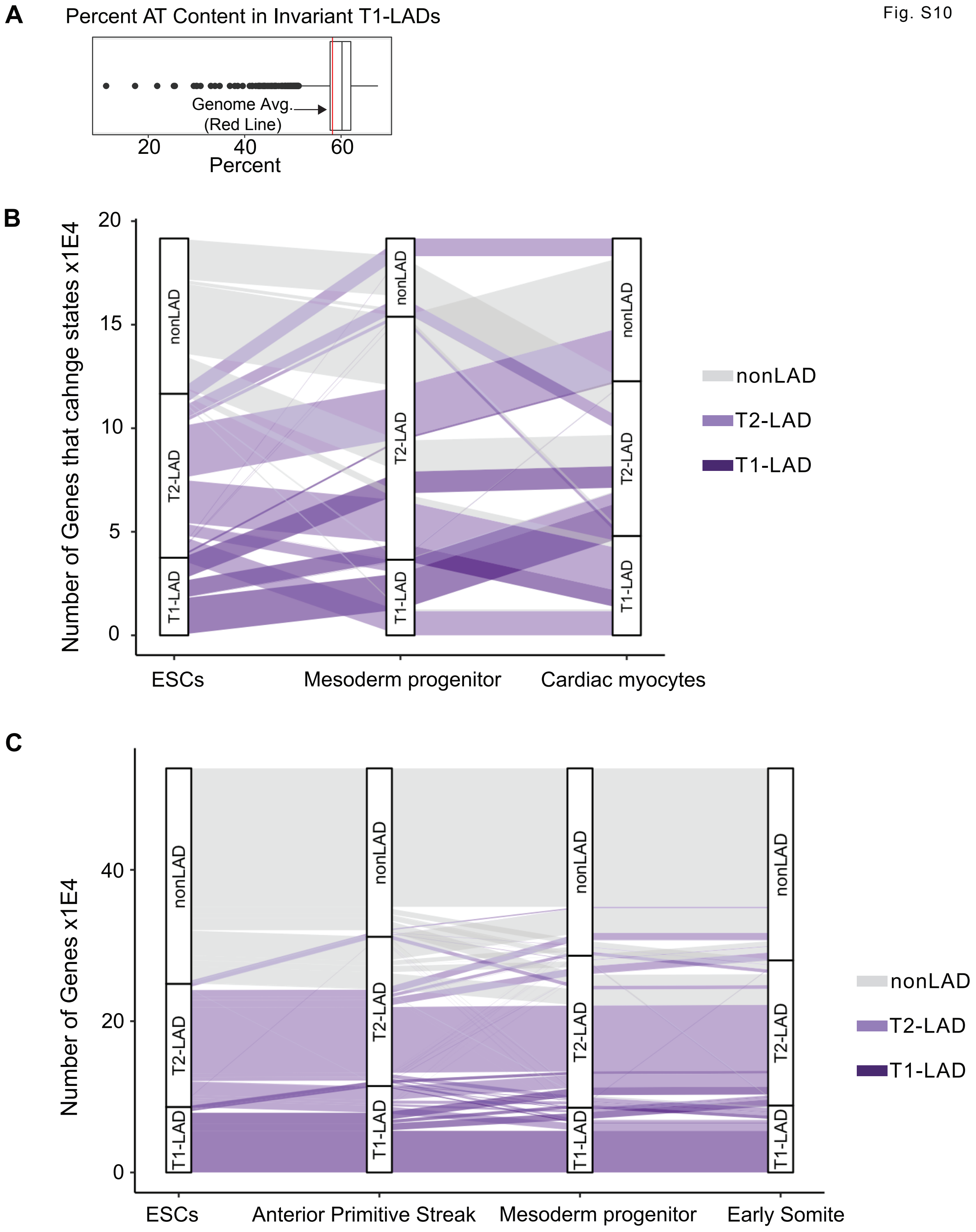
A
Percent AT Content in Invariant T1-LADs
Genome Avg.
(Red Line)
20
40
60
Percent
B
Number of Genes that cahnge states x1E4
0
5
10
15
20
nonLAD
T2-LAD
T1-LAD
ESCs
Mesoderm progenitor
Cardiac myocytes
nonLAD
T2-LAD
T1-LAD
C
Number of Genes x1E4
0
20
40
nonLAD
T2-LAD
T1-LAD
ESCs
Anterior Primitive Streak
Mesoderm progenitor
Early Somite
nonLAD
T2-LAD
T1-LAD

Additional file 2: Fig. S1: Validation of ESC-derived cell types. A-F. Genes as indicated. Panels in (A, C) (left), and (E) (left) show downregulation of pluripotency genes as cells differentiate into definitive ectoderm, border ectoderm, and mid-hindgut. All other panels show enrichment of lineage-specific genes, as indicated. For all assessments, relative enrichment is shown (normalized to ESCs). n=3 biological replicates. Error bars=SEM. G. Schematic of epicardial specification. H. WT1 and DAPI staining of cardiac cells (left) and epicardial cells (right). I. Quantification of WT1 positive and negative cells from (H). J. Schematic of endothelial specification. K. Endothelial cell sorting using FSC and CD31 markers. L. Schematic, cardiac differentiation. M. Cardiac cells show canonical TNNT2 expression.

Additional file 2: Fig. S2: LB1 antibody ChIP and specificity validation; 3-state HMM validations. A. LB1 ChIP-immunoblot of ESCs shows enrichment of LB1 in LB1 ChIP (lane 1) compared to no antibody and IgG controls (lanes 3 and 4, respectively). B. LB1 antibody specificity measured by ChIP immunoblot in mesoderm progenitors and mid-hindgut cell lysates with and without peptide competition shows LB1 enrichment in ChIP samples without peptide (lanes 1 and 2), but not in ChIP with LB1 peptide (lane 3). C. AIC differences between numbers of LAD states. D. BIC differences between numbers of LAD states. E. Size range of T1-LADs (dark purple) and T2-LADs (light purple) shown across the atlas datasets; average of all cell types shown on the left. Note that the y-axis is a logarithmic scale.

Additional file 2: Fig. S3: Validation of LADs identified by a 3-state HMM. A. Percent of T1-, T2-, and nonLAD 20kb bins with log2 ChIP/input > 0 shown for each replicate across atlas cell types. The majority of T1- and T2-, but not nonLAD bins, show LB1 enrichment. B. Segregated LB1 (top) and input (bottom) reads assigned to ESC T2-LADs and nonLADs. T2-LADs have significant LB1 enrichment compared to nonLADs (One-tailed Mann–Whitney U test: $U=6.337\times10^{9}$, $p<2.2\times10^{-16}$); nonLADs are not LB1 enriched relative to input. C. The percent of overlapping bp from replicate HMM results for LADs across the atlas. D. LB1 enrichment measured by ChIP-qPCR in three LAD states. T1-LAD and T2-LADs are differentially enriched for LB1. Each box represents ChIP/input average of three independent ChIP-qPCR assessments at 20 loci. E. (Top) DamID and LB1ChIP-seq tracks and annotated LADs across Chr. 3 show a high degree of concordance. (Bottom) Zoomed-in view of highlighted area shows low DamID signal and DamID-identified LADs over regions of low ChIP-seq LB1 signal in gray boxes. Shaded boxes highlight regions of low DamID signal that are identified as LAD regions in the DamID dataset. F. Overlap (bp to bp) between DamID-identified and HMM-identified ChIP-seq LADs shows nearly all DamID bp (99.6%) represented in T1- and T2-LADs. 98.1% of T1-LAD bp and 47.4% of T2-LAD bp are represented in DamID LADs compared to 0.1% of nonLAD bp. G. Quantification of Dam control-normalized DamID LB1 enrichment in ChIP-seq T1-LAD, T2-LAD, and nonLADs (left) and DamID LADs. F. GC content across HMM-LADs identified in atlas cell types.

Additional file 2: Fig. S4: LAD transition state parameters. HMM transition parameters from each cell type-specific model (log2-transformed for visualization purposes) for LADs.

Additional file 2: Fig. S5: H3K9me2 antibody ChIP and specificity validation; 3-state KDD validations. A. ESC H3K9me2 ChIP-immunoblot shows enrichment of H3K9me2 in H3K9me2 ChIP (lane 3) compared to input and no antibody controls (lanes 1 and 3, respectively). B. H3K9me2 antibody specificity measured by dot blot shows positive signal in total ESC cell lysate (lane 1, dots 1 and 2) and with H3K9me2 peptide (lane 1, dot 4), but not H3K9me1 or H3K9me3 (lane 1, dots 1 and 3, respectively). No antibody binding detected in buffer only controls (lane 2). C. AIC differences between numbers of KDD states. D. BIC differences between numbers of KDD states.

Additional file 2: Fig. S6: H3K9me2 HMM validation. A. H3K9me2 binding in KDDs for each cell type. B. H3K9me2 enrichment measured by ChIP-qPCR in three KDD states. C. The percent of overlapping bp from replicate HMM results for KDDs across the atlas. D. Sizes of T1-KDDs (dark green) and T2-KDDs (light green) shown across the atlas datasets.

Additional file 2: Fig. S7: KDD transition state parameters. HMM transition parameters from each cell type-specific model (log2-transformed for visualization purposes) for KDDs.

Additional file 2: Fig. S8: KDDs and LADs are highly overlapping; T2-LADs overlap vLADs. A. Example track of a genomic region with overlapping LADs and KDDs in indicated cell types. B. H3K9me2 occupancy in LADs for all atlas cell types shows greatest H3K9me2 in T1-LADs. C. Actual expression values shown for the expressed genes (>0.5 tpm) corresponding to the discretized data shown in Fig. 2C. D. Comparison of ESC ChIP-seq LADs to DamID LADs from 9 immortalized cell lines (from Kind et al., 2015) show greater overlap of T2-LADs with vLADs compared to cLADs. E. Proportion of T1-LAD, T2-LAD, and nonLAD bins enriched for LB1 (log2 ChIP/input > 0) across a range of bin sizes. Proportion of enriched T2-LAD bins decreases as size increases. Data points represent the percentage of enriched bins/all bins per LAD. The line indicates the mean percentage at the given bin size; error bars indicate standard deviation.

Additional file 2: Fig. S9: IF-FISH probes, H3K9me2 IF and cell sorting for IF-FISH. A. Median LB1 signal (percentile) of each FISH probe LAD for ESCs; probes designed to avoid strongest and weakest LADs. B. DamID and LB1 ChIP-seq tracks for each ESC FISH probe (probe indicated by blue box). (C) Quantification of T1- and T2-LAD (E4, E5) loci in ESCs ($p<0.01$, Mann-Whitney test). D. Representative image of H3K9me2 IF in ESCs and cardiac myocytes. Quantification of H3K9me2 signal to the nuclear periphery (5 random measurements in n=10 nuclei per cell type; see Methods). Scale bars = 1μM. E. Fluorescence activated cell sorting for cardiac myocyte populations via SIRPA. F. Median LB1 signal (percentile) of each FISH probe LAD for cardiac myocytes; probes designed to avoid strongest and weakest LADs. Note: Two probes for T1-LADs were designed on the 5' and 3' end of the same large LAD, and are represented by a single line. G. LB1 ChIP-seq tracks for additional T1-LAD and T2-LADs probed by IF-FISH in cardiac myocytes (probe indicated by blue box). H. Quantification of T1- and T2-LAD (CM4, CM5) loci in cardiac myocytes ($p<0.01$, Mann-Whitney test).

Additional file 2: Fig. S10: Genes change LAD assignments between cell types. A. Invariant T1-LADs have a median of 60% AT content, a slight increase over the genome (58% AT content, red line). B. Figure Panel 6C shown without the non-changing LADs to underscore those genes that are changing LAD designation/assigned states. C. Gene LAD assignment changes across cells from the mesoderm lineage, as indicated. Genes rarely move from T1-LADs to nonLADs, with T2-LADs showing the greatest gene occupancy gains and losses.
