# Supplementary material for: An atlas of lamina-associated chromatin across twelve human cell types reveals an intermediate chromatin subtype
Source: Genome Biol. 2023 Jan 23;24:16. doi: 10.1186/s13059-023-02849-5 (PMC9869549; doi:10.1186/s13059-023-02849-5)
Supplement: Supplementary file 10 — Additional file 10. Review history. [file 13059_2023_2849_MOESM10_ESM.docx]

Review History

**First round of review**

**Reviewer 1**

**Were you able to assess all statistics in the manuscript, including the appropriateness of statistical tests used?**

Yes, and I have assessed the statistics in my report.

**Comments to author:**

In this manuscript Keough and colleagues present a catalogue of LAD profiles in 12 human cell types of diverse origin. The authors characterize three states of genome-lamina associations based on 3-state HMM domain algorithm. The subcategories differ in their level of lamina enrichment from high to low. The lamina high regions resemble cLADs or HiLand-P regions, while the intermediate lamina regions represent fLADs or HiLand-B regions. The authors continue to show that the regions with intermediate lamina enrichments represent cell type-specific interactions and that these regions generally localize to a nuclear subdomain just beneath the H3K9me2-enriched chromatin at the periphery. Finally, the authors show that only 7% and 1% of the regions with high and intermediate lamin enrichments respectively are cell type-invariable. This work represents a valuable resourse for the community as no such extensive dataset has been published yet. However, most of the finding presented in this manuscript are not novel and have been observed in previous publications (see below).

Major comments

* My main concern with this manuscript is its novelty. All the concepts that are described in this work have been reported before. The observation of two different LAD states has been made several times by different groups (e.g. Meuleman et al., 2013, Kind et al., 2015 and Zheng et al., 2015). These regions have been shown to be associated with different genomic features very similar to what is reported in the current manuscript. The previously annotated fLADs or HiLand-B regions are regions of reduced lamin-contacts and display cell-type specificity (as also reported here).

* Although not published, a LAD-atlas of 9 different human cell lines does excist and is available. See Kind et al., 2015 "We used a collection of conventional microarray-based DamID maps of NL interactions in 9 human cell lines of diverse origin (CAdG and BvS, manuscript in preparation; see Extended Experimental Procedures)".

* Domain calling is very tricky and to get it right is a tedious process of trial and error. However, the domain calling presented in this manuscript, and visually exemplified in Fig.1b, does not appear to capture the "true" LAD-structure very well. The 3-state HMM appears to create a LAD-state that is 1) often not enriched over baseline, 2) in signal amplitude varies extensively between domains and 3) has different characteristics related to the genomic embedding of the domain (within a big LADs or in isolation (see also comment below for H3K9me2)). Also, the algorithm appears to behave differently for different cell types. E.g. for this genomic view, in the midbrain and border ectoderm samples this entire region is annotated as lamina associated (T1 or T2), while there are many regions that are at or below baseline. Annotating such regions as LADs is not correct and may compromise accurate data interpretation in my view. Possibly, the relatively limited dynamic range (-1 to 1 Log2?) of the data has impacted on the performance of the HMM?

* In line with the above comment, again in Fig. 2a the 3-state HMM does not perform very well on H3K9me2 data. E.g. the big domain at ~65Mb (no tick marks so this is a guess) in border ectoderm is annotated to constitute three T1 and T2 domains of all high H3K9me2 levels. The reason the 3-state model annotates this as 6 separate domains is because of its sensitivity for detecting transitions in the data. Yet, the annotated T2 domains in this region are much more enriched for H3K9me2 than e.g. any of the T1 regions in the midbrain sample. And the annotated T2 regions for this big domain have much higher H3K9me2 levels compared to the T2 domains further downstream of the chromosome. In my opinion, this is a solid single T1-type domain.

* In line with the above, and to obtain better insight into data quality, it would really help to have more quantitative and qualitative comparison between this data and previously generated profiles with DamID. Most resources on LADs are obtained with DamID. Therefore, for accurate interpretation of this atlas in light of previous findings, it is very important to have a direct comparison between the two methods. What is the percentage overlap between the combined ChIP (T1 and T2) LADs and DamID LADs in hESC? Judging from the domain calling in Fig. 1b this percentage is lower than 90% (Fig. S3D).

Minor comments

Fig.1b—it is not clear what is plotted on the y-axis. Log2 ChIP/input?

Fig. 1D-E—In Fig. 1D the baseline enrichment of T2 LADs is just above 0. Perhaps it is a matter of semantics, but I would not call genomic regions that just barely are enriched above baseline to interact with the lamina. Also, can the authors explain why the baseline for T2 LADs is different between Fig. 1D (~0.10) and Fig. 1E (~0.25)? This should be equal, especially considering the chosen intervals (500kb) and the average domain length of T2 LADs (280kb).

Fig. 2C—Please show the actual expression values here instead of the discretized (yes or no expression) data.

Fig. 2D—this figure is not very intuitive to interpret. Please reconsider data visualization. Perhaps, split the data by type and present them separately for all cell lines.

Fig. S3C—relative enrichment. What is the metric used here?

Fig. 3B-C—In these panels, the CTCF binding peak is located at least 10kb away from the boundary. This increase of CTCF binding therefore is not found at the boundary. Perhaps, reconsider rephrasing the text corresponding to this observation (lines 31-42). This result is also at odds with previous findings (Guellen et al., 2008). Do the authors think this is related to differences between ChIP and DamID, or perhaps to the type of domain-calling implemented in this manuscript (see my previous comments). Also, the difference in rpkm values between the highest and lowest peak is relatively small (~0.2-0.3 rpkm).

Line 45: the citation to Kind et al., 2013 should be Kind et al., 2015.

Fig. 4C—from the genomic profiles it is expected that the probe in the ESCs localizes closer to the periphery compared to the same probe in the cardiac myocytes. Yet, the quantification shows the opposite pattern. Can the authors comment on this?

Fig. 5—the 7% invariable lamina contacts is a surprisingly low number. This is arguable the most interesting finding of the manuscript. To exclude a data-technical explanation, could the authors determine the percentage of overlap between two or three closely related cell lines? E.g. belonging to the same tissue type? If this observation is truly biological, one would expect that the overlap in T1 LADs is much higher than 7%. If this observation has a strong technical component, then even between similar cell types technical variations will display low concordance. Similarly, it would be reassuring to see the concordance in domain calling between technical replicates of the same cell type.

Fig. 6A—this should be log2 fold chance I presume? Also, it took me a long time to interpret this figure and I am still not sure I fully grasp it. Please consider presenting this data in a more intuitive manner.

**Reviewer 2**

**Were you able to assess all statistics in the manuscript, including the appropriateness of statistical tests used?**

Yes, and I have assessed the statistics in my report.

**Comments to author:**

In this work, the authors have constructed a large resource of Lamina Associated Domain (LAD) data, collected using ChIP-seq across 12 cell types. This is a substantial increase over the previously available number of human cell types profiled.

In addition to Lamin B1 ChIP-seq data, the authors have generated H3K9me2 ChIP-seq data for the same cell types, to aid in the characterization of subtypes of Lamina Associated Domains (LADs).

This represents a valuable data resource for the chromatin biology and genome organization communities. I do however have several concerns with the clarity with which experiments and analyses are laid out, and a few more technical comments

1.Quality of generated data and concordance with previously published data On page 6, the authors report generally high Pearson correlations between same-biosample replicates. However, given the continuous nature of the data and the subsequent segmentation into three HMM states, correlation coefficients are not a sufficient metric of replication. Rather, have the authors attempted to call states using their HMM approach on individual replicates, and subsequently assess concordance between state calls?

Related, how exactly do the currently presented data compare to previously generated ChIP-seq and DamID maps of Lamin B1 in comparable human cells? Do LAD/non-LAD calls from previously published efforts align with those reported here? It is important to carefully assess and describe this, especially given how the current study uses a different assay type (ChIP-seq) than most previously published Lamin B1 data (DamID).

2. Number of distinct lamina-related states The authors report a choice of three HMM model states to capture qualitative differences in lamina-association. This choice is based on the AIC and BIC differences between models with increasing state numbers. The trends shown in Supp. Figure 3A&B are predictable/expected and do not by themselves justify the choice of 3 states: increasing the number of states will generally result in diminishing AIC/BIC differences. Rather than relying on these metrics, one would want to see a level of robustness of state calling, for instance by further investigating state call concordance between same-biosample replicates.

3. General characteristics of T1, T2 and non-LADs To what extent can the T1/T2/non-LAD designations be predicted by sequence composition e.g. %GC content, in particular for ESC vs. other cell types (cf. Meuleman et al. 2013, which showed that in particular ESC-LAD calls largely follow %GC content).

The authors mention (Page 7, line 45) that T1 and T2 domain sizes vary greatly, yet this is not particularly reflected in the reported summary statistics. Perhaps a box or violin plot would better convey these perceived differences, or lead to the conclusion that this statement is too strong.

4. Differences between T1 and T2 LADs The authors convincingly show that T1 and T2 LADs differ in terms of various characteristics, including level of Lamin B1 association, gene density and expression, etcetera. However, I have a number of concerns with some of the spatial localization analyses related to this.

First, to investigate whether the radial positioning differs between T1 and T2 LADs, the authors use IF-FISH to visualize the spatial positioning of a small number of loci in ES cells and cardiac myocytes. The validity and interpretability of such an experiment depends heavily on how these loci were selected. Can the authors elaborate on this procedure? Do these loci perhaps represent e.g. the most confidently identified T1/T2 LADs?

Related, what do these loci look like in Lamin DamID data, e.g. the selected T2 locus in human ESCs (chr12:65.7-67.9Mb, Fig. 4A)?

If T1 and T2 LADs are truely different types of LADs, and not merely less/more dynamic ones, such loci might show a different picture in DamID, since DamID does not result in a static snapshot but rather an accumulation of Lamin association over time, which might saturate in T1 LADs and not (yet) in T2 LADs. If so, does DamID data indeed show a clearer binary LAD/non-LAD pattern? Could this explain any observed differences between DamID and ChIP-seq data? If so, this would be an attractive feature of the current study and its data and is worth pointing out.

Regarding Figure 4C, I'm confused by the selection of the additional two loci that seemingly span T1 and T2 LADs in either ESC or cardiac myocytes. If the objective is to probe for the spatial localization of T2 LADs, why further complicate things by introducing more uncertainty in terms of LAD 'types'?

The statement that T2 LADs are due to different radial positioning rather than the result of dynamic positioning (page 12) is an intriguing one and warrants some more exposure. If indeed the case, how do T2 LADs obtain their Lamin B1 association? Does this imply that Lamin B1 occurs in large enough quantities outside of the nuclear rim, as opposed to H3K9me2 (Supp. Fig. 6A)?

5. Potential role of T2-LADs The authors suggest that T2-LADs may be a transition step (between non-LADs and T1-LADs) for gene activation and expression (pages 13, 14 and elsewhere). This is an interesting possibility, and although the authors have attempted to visually represent this in Figure 6C, I feel like this would be conveyed better by focusing on loci that actually change LAD-status, since the majority of LAD calls remains stable in Fig. 6C.

Related, to what extent is this also reflected in transitions between genomic loci? Concretely, do the HMM state transition parameters perhaps conceptually (also) look like e.g. "non-LAD <--> T2-LAD <--> T1-LAD", and can this be shown in a separate figure?

6. Variability across cell types Interestingly, as opposed to previous studies based on smaller numbers of cell types, the authors find that most LADs vary across the 12 cell types assayed here. To investigate whether this is simply a matter of more exhaustively sampling cell type space, or a more inherent to the T1/T2 LAD subtype designation, it would be good to plot the percent invariance as a function of number of randomly sampled cell types. So for a random sample of, say, 4 cell types a level of invariance of XX% percent is observed, while for a random sample of, say, 8 cell types that invariance reduces to XX%.

Related to this, are T1-LADs more likely to remain stable across (any two given) cell types than T2-LADs? If so, how do T2-LADs relate to the 'facultative LADs' described in Meuleman et al. 2013? If there is not a strong relation there, the possibility of T2-LADs being more dynamic within a cell population should more clearly be considered, as opposed to suggesting otherwise (page 12). 7. Although the authors present an impressive array of cell types, in neatly defined differentiation trajectories, most analyses rely on ESCs and caridac myocytes, which to this reviewer seems to be a bit of a missed opportunity. What is the reason for this?

8. Gene Ontology (GO) enrichment analyses The authors describe the use of GO enrichment analyses to assess the type of genes located in invariant T1 and T2 LADs (page 13), against a background of all human (protein-coding) genes. However, this will bias results towards genes that are generally located inside LADs. Rather, using a background consisting of only genes inside (T1 or T2) LADs would be more appropriate.

**Authors Response**

**Point-by-point responses to the reviewers’ comments:**

***Reviewer #1:*** *In this manuscript Keough and colleagues present a catalogue of LAD profiles in 12 human cell types of diverse origin. The authors characterize three states of genome-lamina associations based on 3-state HMM domain algorithm. The subcategories differ in their level of lamina enrichment from high to low. The lamina high regions resemble cLADs or HiLand-P regions, while the intermediate lamina regions represent fLADs or HiLand-B regions. The authors continue to show that the regions with intermediate lamina enrichments represent cell type-specific interactions and that these regions generally localize to a nuclear subdomain just beneath the H3K9me2-enriched chromatin at the periphery. Finally, the authors show that only 7% and 1% of the regions with high and intermediate lamin enrichments respectively are cell type-invariable. This work represents a valuable resourse for the community as no such extensive dataset has been published yet. However, most of the finding presented in this manuscript are not novel and have been observed in previous publications (see below).*

*Major comments*

*1. My main concern with this manuscript is its novelty. All the concepts that are described in this work have been reported before. The observation of two different LAD states has been made several times by different groups (e.g. Meuleman et al., 2013, Kind et al., 2015 and Zheng et al., 2015). These regions have been shown to be associated with different genomic features very similar to what is reported in the current manuscript. The previously annotated fLADs or HiLand-B regions are regions of reduced lamin-contacts and display cell-type specificity (as also reported here).*

*We thank the reviewer for appreciating that this atlas represents a valuable resource for the community, but we respectfully disagree regarding the lack of novelty or advance in this study. We absolutely acknowledge that this study follows a body of work established by many seminal discoveries in LAD biology, including the observation of LAD variability between cell types and the existence of stronger and weaker lamin-enriched domains. We discuss and cite these studies throughout our manuscript, and in this revision we have expanded the background and citations to clarify the work upon which our study builds.*

*Our goal for this study is to provide a large, published dataset for public use - created from non-immortalized human cells, using cell types derived from common progenitors and representing multiple developmental trajectories - that supports and extends existing LAD models. We have had several requests to share our datasets for other studies. A key novelty of our study is our generation of parallel H3K9me2 and LB1 occupancy atlases, which expand upon existing resources that lack histone profiles. Importantly, we generated this data for twelve cell types from all three germ layers.*

*Another advance is our identification of two types of LADs within a single cell type with different epigenetic and molecular characteristics. As we present in greater detail below (see response to Point 5 and to Rev. 2, Point 6b), T2-LADs partially overlap and share similarity with previously-described facultative/variable LADs. However, we do not equate T2-LADs to fLADs/vLADs because T2-LADs are not defined by LAD changes between cell types. Some T2-LAD regions remain as T2-LADs across the twelve cell types and three germ layers represented in our atlas datasets, while other T2-LAD regions change between cell types. Furthermore, T1-LADs are defined based on high LB1 signal within a cell type, which does not equate with being invariable across cell types; some T1-LADs are invariable but most are not.*

*Another strength of our work is examining how population-based LAD states impact single-cell LAD radial positioning. Because T1- and T2-LADs are defined within a single cell type, we are able to assess how LB1 enrichment differences at specific LAD regions are related to the relative positioning of those LADs to each other and the nuclear periphery. Our data support a model in which T2-LAD regions are “intermediately-positioned” between T1-LADs and nonLADs. This model is further supported by the observation that T2-LADs are later replicating than nonLAD regions, but not as late as T1-LADs (Fig. 3A). As shown in the Response Appendix, T1- and T2-LADs are further distinguished by enrichment for different SPIN (Spatial Position Inference of the Nuclear genome* [*[1]*](https://paperpile.com/c/xf8ftO/hVU1) *compartments (see Appendix, p. 26).*

*Therefore, we believe that the data presented in this study both support and advance existing models of LAD biology, providing additional insight into the complexity of organization and compartmentalization that likely exists at the nuclear periphery. As a resource, we intend this dataset to be a foundation upon which other data can be overlaid and integrated, to gain greater clarity and knowledge of peripheral chromatin organization. Given the growing interest in understanding the role of 3D genome organization across different organ systems and tissue types, we predict our dataset will be used by a variety of scientists. Many groups have reached out to us to access and study our datasets since we posted the preprint. In accord, Reviewer 2 writes that “This is a substantial increase over the previously available number of human cell types profiled…This represents a valuable data resource for the chromatin biology and genome organization communities.”*

*2. Although not published, a LAD-atlas of 9 different human cell lines does excist and is available. See Kind et al., 2015 "We used a collection of conventional microarray-based DamID maps of NL interactions in 9 human cell lines of diverse origin (CAdG and BvS, manuscript in preparation; see Extended Experimental Procedures)".*

*We acknowledge that there have been many important LAD studies that have come prior to this atlas. However, in contrast to the referenced data, we note that our dataset is derived from established human ESC lines that are untransformed and competent for differentiation along multiple developmental trajectories, resulting in the many cell types and datasets we created for the atlas. One benefit therein is the ability to assay LAD organization and changes at intermediate developmental time points (e.g., ESC to mesoderm progenitor to cardiac myocyte), an important advance over existing datasets mapping LADs across multiple distinct cell lines. Given the increasing prevalence of ESC-based differentiation strategies, we feel that this atlas is an important resource to complement the existing 9-cell-type DamID dataset. Per a suggestion from Reviewer 2, in the revised manuscript we have compared the atlas data to this referenced 9-cell-type DamID dataset, observing high concordance, but some important differences, between T2-LADs defined within ESCs (the common cell types) and constitutive and facultative DamID LADs (see Rev. 2, Point 6b; Response Fig. 19).*

*3. Domain calling is very tricky and to get it right is a tedious process of trial and error. However, the domain calling presented in this manuscript, and visually exemplified in Fig.1b, does not appear to capture the "true" LAD-structure very well. The 3-state HMM appears to create a LAD-state that is 1) often not enriched over baseline, 2) in signal amplitude varies extensively between domains and 3) has different characteristics related to the genomic embedding of the domain (within a big LADs or in isolation (see also comment below for H3K9me2)). Also, the algorithm appears to behave differently for different cell types. E.g. for this genomic view, in the midbrain and border ectoderm samples this entire region is annotated as lamina associated (T1 or T2), while there are many regions that are at or below baseline. Annotating such regions as LADs is not correct and may compromise accurate data interpretation in my view. Possibly, the relatively limited dynamic range (-1 to 1 Log2?) of the data has impacted on the performance of the HMM?*

*We fully agree that domain calling is tricky, and we apologize that the tracks did not accurately depict the data, as they were likely too “zoomed-out.” In the revised manuscript, we have included new, better representative views in Figs. 1B and 2A. Below, we address the reviewer’s important points on 1) variation in signal amplitude, 2) signal enrichment, and 3) cell type-specific differences.*

*It is important to note that the LB1 ChIP-seq occupancy threshold varies along the genome; if it did not vary, then a simple threshold model would work to identify domains. This is the motivation for utilizing the HMM, which can annotate genomic regions where LB1 occupancy is shifted to a sufficient degree from surrounding regions to warrant differential domain categorization. Thus, the ability of the HMM to distinguish different types of LADs based on varying levels of LB1 (Fig. 1C) across the genome in individual cell types is an intended capability. Our analysis of LB1 occupancy at boundaries of T1-/T2-/nonLADs (updated Fig. 1D) supports the discrimination of HMM states with distinct levels of LB1. Consistent with this, upon comparison of DamID LB1 enrichment in DamID-defined LADs from Tier1 human ESCs (4DN Data Portal Identifier: 4DNESNFNTUAO) to our ESC LB1 ChIP-seq, we readily identified DamID regions with modest LB1 occupancy that are annotated as DamID LADs (Response Fig. 1A). In accord, quantification of DamID enrichment in the DamID LADs indicated LADs with a range of signal strength (Response Fig. 1B). Finally, assessment of DamID enrichment in the HMM-identified ChIP-seq LADs also distinguished the LB1 enrichment across the three states, similar to ChIP-seq (Response Fig. 1B, compare to manuscript Fig. 1C). The distribution of DamID signal in T2-LADs is lower than that in T1-LADs and higher than that in nonLADs. Thus, LB1 enrichment from an independent, DamID dataset is consistent with the different levels of LB1 ChIPseq in our HMM-identified ChIPseq LADs. These data have been added to the revised manuscript as Supp. Fig. 3C, E.*

***Response Fig. 1:*** ***Tier 1 ESC DamID and ESC ChIP-seq LADs are highly overlapping.*** *(A) Top panel: DamID (top track in each panel) and ChIP-seq (bottom track) LB1 and LADs shown across Chr. 3. Shaded boxes highlight regions of low DamID signal that are identified as LAD regions in the DamID dataset. Bottom panel: Zoomed-in view of the highlighted area shows low DamID signal and DamID LADs over regions of low ChIP-seq LB1 signal. (B) Quantification of input-normalized DamID LB1 enrichment in ChIP-seq LADs (left) and DamID-defined LADs (right) and show a range of DamID LB1 enrichment above nonLAD signal.*

*Per the reviewer’s concern about signal enrichment and dynamic range, the relative atlas LB1 enrichment range (log2 ChIP/Input, data processing details in Methods) is comparable to other published ChIP-seq LAD maps* [*[2,3]*](https://paperpile.com/c/xf8ftO/AwRc+Vg4m)*. To address the concern of limited LB1 enrichment in the HMM-identified LADs, we predicted LADs using the trained HMMs on individual biological replicates for all cell types (a suggestion also made below in Point 14 and Rev. 2, Point 1a). We expected that LAD regions lowly enriched for LB1, close to the baseline or at the limit of signal:noise, would be categorized as nonLADs in individual replicates as a result of the reduced sequencing depth. Instead, we found a high degree of concordance between the HMM-identified LADs from individual biological replicates across atlas datasets (Response Fig. 2), with the exception of the midbrain sample (discussed further below). This analysis provides confidence in the T1- and T2-LAD designations.*

***Response Fig. 2: HMM-designated LADs from individual replicates show a high degree of replicability across the atlas.*** *Pearson correlation assessments between HMM-identified LADs across biological replicate samples from the LAD atlas show a high degree of correlation between the majority of the cell types and for both LAD subtypes.*

*To provide additional support for our LAD calls, we directly compared the atlas ESC ChIPseq LAD domains to human ESC DamID LAD calls in the dataset referenced above (4DN Identifier: 4DNESNFNTUAO, Response Fig. 3). We observed a high degree of concordance between LADs identified between these two methods; 99.6% of DamID LAD regions (bp to bp overlap) were also identified as a T1- or T2-LAD in the atlas ChIP-seq data (see response for Point 5 for additional discussion and comparison of ChIP-seq atlas LADs to DamID LADs). This data is included in the revised manuscript as Supp. Fig. 3D.*

***Response Fig. 3: Tier 1 ESC DamID and ESC ChIP-seq LADs are highly overlapping.*** *Overlap (bp to bp) of DamID LADs to ChIPseq LADs shows high concordance; nearly all DamID LADs are represented in T1- and T2-LADs.*

*Regarding the reviewer’s point of the HMM behavior across cell types, we now clarify in the manuscript that we intentionally trained models for each cell type independently due to the expectation of different LB1 occupancies in different cell types and experimental batches (see above for Response Fig. 1). In this way, HMM states are defined relative to other genomic regions in the same cell type. Regarding the LADs in the former Fig. 1B track view, the midbrain and definitive ectoderm cell types have LB1 occupancy across the indicated regions, albeit at a relatively lower level than other cell types. The regions not identified as LADs clearly have a depletion of LB1 occupancy, as identified by the signal below the x-axis. The midbrain samples showed the least replicability in HMM classes from individual replicates. We suspect that this difference may arise from the fact that midbrain cells are at intermediate states of lineage restriction. While we do not think this impacts the overall conclusions presented in the study, we are happy to consider removing this cell type if the reviewer feels that would be best. The HMM replicability data are now included in the manuscript as Supp. Fig. 3B.*

*4. In line with the above comment, again in Fig. 2a the 3-state HMM does not perform very well on H3K9me2 data. E.g. the big domain at ~65Mb (no tick marks so this is a guess) in border ectoderm is annotated to constitute three T1 and T2 domains of all high H3K9me2 levels. The reason the 3-state model annotates this as 6 separate domains is because of its sensitivity for detecting transitions in the data. Yet, the annotated T2 domains in this region are much more enriched for H3K9me2 than e.g. any of the T1 regions in the midbrain sample. And the annotated T2 regions for this big domain have much higher H3K9me2 levels compared to the T2 domains further downstream of the chromosome. In my opinion, this is a solid single T1-type domain.*

*We thank the reviewer for pointing this out. We apologize that the coordinates were not easily discerned, and we have modified this and all track figures appropriately with tick marks/chromosome coordinates. As discussed above, the HMM models are trained independently per cell type, and therefore direct comparisons of H3K9me2 occupancy depth between cell types is not factored when assigning H3K9me2 state in a given cell type. One strength of an HMM is its ability to identify changes in signal enrichment relative to the surrounding region, thus accounting for variability in signal depth along the genome within each cell type. The specified region in the former Fig. 2a is an example of the model accurately distinguishing between regions of greater and lower H3K9me2 occupancy to define two separate H3K9me2 states. We have revised the figure with a more representative track view to provide a more accurate depiction of the calls (Fig. 2A). In the manuscript, we also present that T1- H3K9me2 domains (KDDs) are most enriched for LB1 occupancy followed by T2-KDDs (Fig. 2B). In further support of our KDD calls, we also calculated Pearson correlations of KDD replicates (Response Fig. 4; parallel to efforts in Response Fig. 2 above), which show a high degree of replicability in KDD designations across most cell types. Therefore, the main metrics by which we can quantitatively validate the HMM designations provide confidence in the methodology for identification of KDDs across the atlas. The Pearson correlations are now shown in the manuscript as Supp. Fig. 6C.*

***Response Fig. 4: KDDs are highly replicable between biological replicate samples.*** *Pearson correlations of KDDs by replicate show a high degree of replicability for most cell types between HMM-designations from individual biological replicate samples.*

*5. In line with the above, and to obtain better insight into data quality, it would really help to have more quantitative and qualitative comparison between this data and previously generated profiles with DamID. Most resources on LADs are obtained with DamID. Therefore, for accurate interpretation of this atlas in light of previous findings, it is very important to have a direct comparison between the two methods. What is the percentage overlap between the combined ChIP (T1 and T2) LADs and DamID LADs in hESC? Judging from the domain calling in Fig. 1b this percentage is lower than 90% (Fig. S3D).*

*We thank the reviewer for this important suggestion. As requested, we directly compared the combined T1- and T2-LADs from the atlas ESC ChIP-seq LAD calls to human ESC DamID LADs obtained from the 4DN Data Portal (Identifier: 4DNESNFNTUAO; see above for Response Fig. 1A and our related response to Point 3). We observed a high degree of concordance between LADs identified between these two methods; 99.6% of DamID LAD regions (bp to bp overlap; Response Fig. 3, new Supp. Fig. 3D) were also identified in the combined T1- and T2-LADs from the atlas ChIP-seq data. In addition, as demonstrated above in Response Fig. 1B, the distribution of DamID signal in T2-LADs is lower than that T1-LADs and higher than that in nonLADs. We have also included LB1 DamID tracks from ESCs corresponding to the genomic areas we assessed with OligoFISH in ESCs (Supp. Fig. 9B). Finally, though not directly asked by this reviewer, we also compared our LAD calls to DamID variable/facultative LADs; that comparison is presented in response to Rev. 2, Point 6b and Response Fig. 19. Taken together, we hope the reviewer appreciates the more in-depth analysis regarding the relationship of DamID to ChIP-seq in the matched cell type.*

*Minor comments*

*6. Fig.1b—it is not clear what is plotted on the y-axis. Log2 ChIP/input?*

*We apologize that this was not clear. Yes - all the ChIP-seq tracks are shown as log2 LB1 (or H3K9me2) ChIP/input. Accordingly, all track figures have been amended to specifically identify what is being plotted.*

*7. Fig. 1D-E—In Fig. 1D the baseline enrichment of T2 LADs is just above 0. Perhaps it is a matter of semantics, but I would not call genomic regions that just barely are enriched above baseline to interact with the lamina. Also, can the authors explain why the baseline for T2 LADs is different between Fig. 1D (~0.10) and Fig. 1E (~0.25)? This should be equal, especially considering the chosen intervals (500kb) and the average domain length of T2 LADs (280kb).*

*As the reviewer mentioned above, domain calling is very tricky. One reason for this is that occupancy of epigenomic signals such as LB1 and H3K9me2 vary in depth along the genome; therefore, the depth for a T1-LAD at one locus may be different from another locus. This makes identification of LADs and KDDs more difficult because we cannot assign a simple occupancy threshold to identify each type of domain. The strength of our HMM-based approach is that the model distinguishes differences in signal corresponding to changes in state, such as a transition from a nonLAD to a T2-LAD or a T1-LAD to a T2-LAD.*

*We have amended this analysis to focus on 100kb intervals around boundaries to ensure that we are only assessing LB1 signal from T2-LADs and their boundaries. The difference in T2-LAD signal noted by the reviewer was partially due to the large scale we previously used, which led to the inclusion of T1-LAD or nonLAD signal from the other boundary of the T2-LAD. That difference is diminished with the smaller scale. Moreover, we had not stratified T2-LADs that border nonLADs versus T1-LADs at each side or a combination of both features. We have therefore revised this figure to now show all of these possible border combinations, and have contained the analysis to 100kb surrounding the border (Response Fig. 5). Our overall conclusion remains the same: there is a sharp decrease in LB1 at the boundaries of T1-LADs to T2-LADs and from T2-LADs to nonLADs. In this stratified analysis, the difference noted by the reviewer was also driven by the differences in LB1 at T2-LADs flanked by non-LADs as opposed to T1-LADs. We thank the reviewer for helping us improve this analysis and have updated this data in Fig. 1D.*

***Response Fig. 5****.* ***LB1 is sharply reduced between T1- and T2-LADs and between T2- and nonLADs.*** *LB1 occupancy measures at 0.1Mb surrounding T1-LAD and nonLAD borders flanking T2-LADs show sharp changes in LB1 as the HMM states change. The border indicated by the dashed box is displayed on the plot.*

*8. Fig. 2C—Please show the actual expression values here instead of the discretized (yes or no expression) data.*

*We thank the reviewer for the comment. The goal of Fig. 2C is to demonstrate that genes in T1-LADs are more likely to be silenced compared to genes in T2-LADs or nonLADs. As requested, we now provide actual expression values for expressed genes (tpm > 0.5) within these categories below (Response Fig. 6). An ANOVA test followed by Tukey’s HSD test shows a significant difference in expression across states, increasing from T1-LADs to T2-LADs to nonLADs as shown by the box plots (median 0.34, 0.51, and 0.65, respectively). This is now included in the revised manuscript as Supp. Fig. 8C. Per the original point of Fig. 2C, most genes in T1-LADs are not expressed compared to T2-LADs and nonLADs, and therefore cannot be represented in an assessment of actual expression, hence our choice to show discretized data in the main figure.*

***Response Fig. 6: Genes are most highly expressed in nonLADs compared to T1- and T2-LADs.*** *Actual expression values shown for the expressed genes (>0.5 tpm), corresponding to the discretized data shown in manuscript Fig. 2C.*

*9. Fig. 2D—this figure is not very intuitive to interpret. Please reconsider data visualization. Perhaps, split the data by type and present them separately for all cell lines.*

*We thank the reviewer for this comment. To clarify, the goal of the referenced figure was to demonstrate consistent features of LADs across all cell types, though we note that capturing the scope of the full dataset makes it challenging to visualize. We have separated the original figure into two parts, adjusted the size of the panel, and amended the key and figure legend to improve interpretability, to keep the panel with all the cell types together (updated Fig. 2D-E). If the reviewers or editors have additional suggestions to improve the figure, we are happy to implement those suggestions to maximize interpretability.*

*Related to the data in Fig. 2D, for the response, we performed a complementary analysis of SPIN states, compartmentalization, and replication timing (Repli-seq) in ESC T1- and T2-LADs (see Response Appendix, page 26), which corroborate the results shown in Fig. 2E and 3A. B compartment T2-LADs are earlier replicating than T1-LADs, and T2-LADs are enriched for interior repressive SPIN states compared to T1-LADs, consistent with our OligoFISH data.*

*10. Fig. S3C—relative enrichment. What is the metric used here?*

*We apologize for the lack of clarity here. The data shown in Fig. S3C (now Supp. Fig. 3A) are generated by LB1 ChIP-qPCR at 20 different T1-, T2-, and nonLAD regions in ESCs for validation of the HMM. As noted on the axis the relative enrichment refers to LB1 ChIP/input qPCR “signal” over the qPCR amplicon. To avoid any confusion about what is “relative” here, we have adjusted the title to now reflect that these data are “ChIP-qPCR (LB1 ChIP/Input).”*

*11. Fig. 3B-C—In these panels, the CTCF binding peak is located at least 10kb away from the boundary. This increase of CTCF binding therefore is not found at the boundary. Perhaps, reconsider rephrasing the text corresponding to this observation (lines 31-42). This result is also at odds with previous findings (Guellen et al., 2008). Do the authors think this is related to differences between ChIP and DamID, or perhaps to the type of domain-calling implemented in this manuscript (see my previous comments). Also, the difference in rpkm values between the highest and lowest peak is relatively small (~0.2-0.3 rpkm).*

*This is an important point. We have modified our discussion of this data in the revised manuscript (page 10) to reflect that we are assessing CTCF binding in LAD boundary-proximal regions and not directly at the ends of the HMM-designated LAD regions. To motivate looking at proximal regions, we note that LADs are large regions defined in a bulk genomic approach. Thus, we feel that it is reasonable to expect that genomic regions extending 10kb outside of a LAD contribute to LAD boundary regions, especially in population-based assays.*

*Upon careful review of the data in Fig. 3B-C, and comparing them again to Guelen et al. 2008, we continue to observe consistency between our findings. Perhaps the differences suggested by the reviewer are due to differences in scale between the figures. Specifically, Fig. 4h of Guelen et al. 2008 views CTCF enrichment at LAD boundaries within a 400kb up- and downstream window. In our Fig. 3B-C, we sought to look closer at the LAD boundaries, and thus focused on regions 50kb up- and downstream of LAD boundaries. Upon close inspection of Fig. 4h from Guelen et al. 2008, the CTCF peak is indeed just upstream of the boundary, which we similarly show in our manuscript, just at a different resolution. We respectfully note that there may be another discrepancy here to which the reviewer is referring and would ask for further clarification if we have not yet fully addressed the concern on this point.*

*12. Line 45: the citation to Kind et al., 2013 should be Kind et al., 2015.*

*We apologize for any referencing errors. We checked each Kind et al., 2013 reference from the previous version of the manuscript and changed the erroneous Kind et al, 2013 to Kind et al, 2015 - referring to the data generated by single cell DamID that we used for analysis.*

*13. Fig. 4C—from the genomic profiles it is expected that the probe in the ESCs localizes closer to the periphery compared to the same probe in the cardiac myocytes. Yet, the quantification shows the opposite pattern. Can the authors comment on this?*

*We acknowledge that this data was overly confusing and apologize for the lack of clarity here. Different regions were probed in ESCs versus cardiac myocytes in the original Fig. 4C. Given that confusion and the further complication that the T2-LAD probes in Fig. 4C shared some overlap with nearby T1-LADs, which might impact the interpretability of the FISH data (as also pointed out by Rev. 2), we have replaced the data shown in the former Fig. 4C. In the updated manuscript, we present OligoFISH for one additional T1- and T2-LAD region for both ESCs (Response Fig. 7A) and CMs (Response Fig. 7B), and show comparison of radial position for all probes in both cell types together (Response Fig. 7C). These data are included in the revised manuscript as Supp. Fig. 9A-C (ESC), Supp. Fig. 9F-H (CM), and Fig. 4D, respectively. In response to Rev. 2, we also provide the relative “strength” of LAD regions that each of our FISH probes overlap (see below for response to Rev. 2, Point 4a and Response Fig. 13). Briefly, we designed probes in the 25-75th percentile range in LB1 strength for T1-LADs and T2-LADs, in an effort to provide representative regions for FISH. Interestingly, consistent with the reviewer’s comment, the stronger T2-LAD probe in cardiac myocytes (CM4) is closer to the lamina than the weaker T2-LAD probe (CM3; Fig. 4C; Supp. Fig. 9F, H). However, without further studies we are hesitant to make a broader claim. Overall, we observe a clear distinction of T2-LAD probes compared to T1-LAD probes (note the mean distances for T1-LADs versus T2-LADs for all probes in both cell types in Response Fig. 7C).*

***Response Fig. 7: T2-LADs are intermediately positioned between T1-LADs and nonLADs.*** *New T1- and T2-LAD probes (replacing the probes used in the previous Fig. 4C) in ESCs (A) and cardiac myocytes (B) show a similar pattern of intermediate localization between T1-LADs and nonLADs, relative to the nuclear lamina. Distance of all T1-, T2-, and nonLAD probes in this study for ESC and cardiac myocytes shown together in (C) and as a cumulative distribution frequency.*

*14. Fig. 5—the 7% invariable lamina contacts is a surprisingly low number. This is arguable the most interesting finding of the manuscript. To exclude a data-technical explanation, could the authors determine the percentage of overlap between two or three closely related cell lines? E.g. belonging to the same tissue type? If this observation is truly biological, one would expect that the overlap in T1 LADs is much higher than 7%. If this observation has a strong technical component, then even between similar cell types technical variations will display low concordance. Similarly, it would be reassuring to see the concordance in domain calling between technical replicates of the same cell type.*

*We thank the reviewer for raising this interesting point. To clarify, the T1-LAD invariance is 7.2% with respect to the whole human genome and 7.9% with respect to the mappable genome (portion of the genome that has been assigned to a state by the HMM model, ~90% of the whole human genome). We have clarified this in the appropriate results section of the manuscript. Moreover, based on existing LAD data showing LAD changes in regions that include cell type-specific genes* [*[4,5]*](https://paperpile.com/c/xf8ftO/Xrca+pGcU)*, we agree that it is possible that cells that are more closely related (along a generally similar lineage trajectory) would have more LAD invariance – that is, more LADs that are consistent – than those with greater developmental differences. Alternatively, LAD organization may be linked to identity in differentiated cell types, but not follow a path correlating with identity during lineage restriction. To the best of our knowledge, these possibilities have not been addressed in other datasets.*

*To address the reviewer’s point, we first calculated the invariance of all LADs, T1-LADs only and T2-LADs only by sampling all possible combinations of 2-12 cell types (Response Fig. 8). This analysis confirmed that the subset of genomic space occupied in invariant LADs by all cell types in a comparison decreases as the number of cell types increases, with T1-LADs increasingly driving the trend. This is included as Fig. 5B in the revised manuscript.*

***Response Fig. 8: LAD invariance increases as the number of cell types increases.*** *Fraction of invariant LADs across comparisons from 2-12 cell types shows decreased invariance as number of cell types compared increases, with T1-LADs being more invariant than T2-LADs. Lines represent means and colored ribbons represent standard deviation.*

*We then focused on 4 differentiated mesoderm cell types– cardiac myocytes, endothelial progenitors, epicardium, and early somites– and found 52% genome-wide invariance as compared to a median of 40% from a random background sampling of combinations of 4 cell types (Response Fig. 9A). Again, all of these percentages are with respect to the portion of the genome that was assigned a state. In order to extend our analysis, we performed cell type comparisons between LADs of 3 cell types in either the same germ layer or different germ layers, looking specifically at the behavior of T1-LADs (Response Fig. 9B). We include ESCs in the within-lineage comparisons (“same” on x-axis) and exclude them in the across-lineage comparisons (“different” on x-axis). We observe a trend that follows the reviewers’ prediction in both analyses; comparisons within developmental lineages show a greater mean LAD invariance than comparisons across developmental lineages (Response Fig 10B).*

***Response Fig. 9: LAD invariance is lower in more developmentally-related versus less developmentally-related cell types.*** *(A) Genome-wide invariance across all combinations of 4 cell types with the four related mesoderm non-progenitor cell types marked by a red line at the 96th percentile. (B) Comparisons of 3-state T1-LADs show higher invariance between cell types within the same lineage versus cell types in different lineages.*

*Together these results show that LAD invariance is impacted by both the total number of cell types being compared and the developmental lineage relationships between them. We now present these results in the manuscript as Fig. 5B-C.*

*To the reviewer’s final point about concordance between replicate HMM results, as shown above in the response to Point 3 and in Response Fig. 2 (shown again below), we have calculated the correlation between domain calls for biological replicates of the same cell type, which show a high degree of concordance, and have incorporated this into new Supplementary Figure 3B.*

***Response Fig. 2: HMM-designated LADs from individual replicates show a high degree of replicability across the atlas.*** *Pearson correlation assessments between HMM-identified LADs across biological replicate samples from the LAD atlas show a high degree of correlation between the majority of the cell types and for both LAD subtypes.*

*15. Fig. 6A—this should be log2 fold chance I presume? Also, it took me a long time to interpret this figure and I am still not sure I fully grasp it. Please consider presenting this data in a more intuitive manner.*

*We apologize for the lack of clarity in this figure and have amended it to make it clearer that we are showing the log2 fold change in expression of each cell type compared to ESCs. We have adjusted the axis titles to clarify both the data used and the log2 fold change (Response Fig. 10; updated Fig. 6A in the manuscript). We have also categorized the data into genes that move towards T1-LADs (T2-LAD to T1-LAD or nonLAD to T1-LAD or nonLAD to T2-LAD) and vice versa and further edited the figure legend for additional clarity.*

***Response Fig. 10: RNAseq expression changes between cell types correspond to the expected change in LAD state.*** *Fold change in gene expression of each cell type over ESCs for genes that switch LAD states. The LAD state change is indicated by arrows in the panel on the right. Dots indicate means and gray ribbons indicate standard deviation. ANOVA test for difference in means is significant for all four cell type comparisons.*

**Reviewer #2:** In this work, the authors have constructed a large resource of Lamina Associated Domain (LAD) data, collected using ChIP-seq across 12 cell types. This is a substantial increase over the previously available number of human cell types profiled. In addition to Lamin B1 ChIP-seq data, the authors have generated H3K9me2 ChIP-seq data for the same cell types, to aid in the characterization of subtypes of Lamina Associated Domains (LADs).

This represents a valuable data resource for the chromatin biology and genome organization communities. I do however have several concerns with the clarity with which experiments and analyses are laid out, and a few more technical comments.

*We thank the reviewer for their kind comments and grateful for their appreciation of this work as a valuable data resource for the community. We have indeed had several requests to share our datasets for other studies.*

1a. Quality of generated data and concordance with previously published data. On page 6, the authors report generally high Pearson correlations between same-biosample replicates. However, given the continuous nature of the data and the subsequent segmentation into three HMM states, correlation coefficients are not a sufficient metric of replication. Rather, have the authors attempted to call states using their HMM approach on individual replicates, and subsequently assess concordance between state calls?

*We thank the reviewer for this suggestion and have carried out the analysis, calculating the Pearson correlation between state calls for biological replicates of each cell type (Response Fig. 2, shown again below). The resulting data demonstrate high concordance of LAD state calls between replicates, with T1-LADs being higher than T2-LADs. We have also performed this same analysis for KDDs (Response Fig. 4, shown again below). The lowest correlation for both LADs and KDDs was in the midbrain sample, and this difference may arise from the fact that these cell types are intermediate states of lineage restriction, and hence more heterogeneous. While we have noted the reduced correlation between replicates for these cell types, we are happy to remove this cell type if the reviewer feels that would be best. The Pearson correlation results are now included in new Supplementary Figs. 3B and 6C.*

**Response Fig. 2: HMM-designated LADs from individual replicates show a high degree of replicability across the atlas.** Pearson correlation assessments between HMM-identified LADs across biological replicate samples from the LAD atlas show a high degree of correlation between the majority of the cell types and for both LAD subtypes.

**Response Fig. 4: KDDs are highly replicable between biological replicate samples.** Pearson correlations of KDDs by replicate show a high degree of replicability for most cell types between HMM-designations from individual biological replicate samples.

1b. Related, how exactly do the currently presented data compare to previously generated ChIP-seq and DamID maps of Lamin B1 in comparable human cells? Do LAD/non-LAD calls from previously published efforts align with those reported here? It is important to carefully assess and describe this, especially given how the current study uses a different assay type (ChIP-seq) than most previously published Lamin B1 data (DamID).

*As requested by this reviewer and Rev. 1, we directly compared our ESC ChIPseq LAD domains to human ESC DamID LADs obtained from the 4DN Data Portal (Identifier: 4DNESNFNTUAO) (Response Fig. 3, shown again below). We observed a high degree of concordance between LADs identified between these two methods; 99.6% of DamID LAD regions (bp to bp overlap) were also identified as a T1- or T2-LAD in the atlas ChIP-seq data (please see the response for Point 5 for additional discussion and comparison of ChIP-seq atlas LADs to DamID LADs). While other DamID datasets are available, we are unaware of any matched cell type DamID data. If the reviewer has additional recommendations for matched datasets for comparison, we are happy to execute those comparisons.*

**Response Fig. 3: Tier 1 ESC DamID and ESC ChIP-seq LADs are highly overlapping.** Overlap (bp to bp) of DamID LADs to ChIPseq LADs shows high concordance; nearly all DamID LADs are represented in T1- and T2-LADs.

*Consistent with the above, we readily identified regions from the above referenced DamID dataset that are annotated as DamID LADs and have reduced LB1 occupancy (Response Fig. 1A, shown again below). Quantification of DamID enrichment in the DamID LADs indicated LADs with a range of signal strength (Response Fig. 1B). Finally, assessment of DamID enrichment in the HMM-identified ChIP-seq LADs also distinguished the signal enrichment across the three states, with T2-LADs having intermediate signal in DamID, similar to ChIP-seq (Response Fig. 1B; compare to manuscript Fig. 1C). Therefore, LB1 enrichment from an independent, DamID dataset is consistent with the differential ChIPseq enrichment in the HMM-identified ChIPseq LADs.*

**Response Fig. 1:** **Tier 1 ESC DamID and ESC ChIP-seq LADs are highly overlapping.** (A) Top panel: DamID (top track in each panel) and ChIP-seq (bottom track) LB1 and LADs shown across Chr. 3. Shaded boxes highlight regions of low DamID signal that are identified as LAD regions in the DamID dataset. Bottom panel: Zoomed-in view of the highlighted area shows low DamID signal and DamID LADs over regions of low ChIP-seq LB1 signal. (B) Quantification of input-normalized DamID LB1 enrichment in ChIP-seq LADs (left) and DamID-defined LADs (right) and show a range of DamID LB1 enrichment above nonLAD signal.

2. Number of distinct lamina-related states. The authors report a choice of three HMM model states to capture qualitative differences in lamina-association. This choice is based on the AIC and BIC differences between models with increasing state numbers. The trends shown in Supp. Figure 3A&B are predictable/expected and do not by themselves justify the choice of 3 states: increasing the number of states will generally result in diminishing AIC/BIC differences. Rather than relying on these metrics, one would want to see a level of robustness of state calling, for instance by further investigating state call concordance between same-biosample replicates.

*We thank the reviewer for this important comment. As requested, to demonstrate the robustness of state calling, we calculated the Pearson correlation between state calls for biological replicates of each cell type (see above for response to Point 1a). This demonstrates high concordance of state calls between replicates generally, albeit with lower correlation for midbrain, as discussed above.*

*We agree with the reviewers that choosing a number of states is not straightforward, even when considering statistics such as AIC/BIC. As described in the text, our goal was to fit the simplest model that accurately describes the data. As shown in Supp. Figs. 2C-D, we saw diminishing returns in AIC/BIC beyond 3 states. To address this comment, we compared LAD calls from 2, 3, and 4-state HMMs to LADs identified based on DamID (4DN portal, Identifier: 4DNESNFNTUAO). The Jaccard similarities (see table below) for all LADs and T1-LADs from 2- and 3-state designations compared with DamID-based LADs were similar (0.81 and 0.74, respectively), suggesting that T1-LADs generally correlated with DamID LADs while T2-LADs from the 3-state model identify a distinct type of LAD with lower LB1 occupancy that is often but not always is identified as a LAD in DamID data. T1-LADs from the 4-state model, however, had a notably lower Jaccard similarity to DamID-based LADs (0.55). Furthermore, T2-LADs from the 4-state model had a much higher overlap with DamID-based LADs (0.33) compared with T2-LADs from the 3-state model (0.13). This suggests that the 4-state model artificially splits the strongest group of LADs into 2 categories. We do note, though, that with additional data types, biological meaning may be gleaned from additional states, perhaps taking into context other information about chromatin states (see Response Appendix, Page 26). We modified the text of the results and discussion to include these important points about our choice of the number of HMM states.*

Jaccard similarities with DamID LADs:

|  | T1-LADs | T2-LADs |
| --- | --- | --- |
| 2 state model | 0.81 | NA |
| 3-state model | 0.74 | 0.13 |
| 4-state model | 0.55 | 0.33 |

3a. General characteristics of T1, T2 and non-LADs. To what extent can the T1/T2/non-LAD designations be predicted by sequence composition e.g. %GC content, in particular for ESC vs. other cell types (cf. Meuleman et al. 2013, which showed that in particular ESC-LAD calls largely follow %GC content).

*We calculated the percent GC content for T1-, T2- and nonLADs for all cell types assessed in this study (Response Fig. 11). Similar to previous studies* [*[5]*](https://paperpile.com/c/xf8ftO/pGcU)*, we found that T1-LADs have the lowest GC content, followed by T2-LADs, and then nonLADs for each cell type; we now include this data as Supp. Fig. 3F in the manuscript. Per the reviewer’s suggestions, we specifically assessed GC content in ESCs and found that mean GC content per LAD category is similar in ESCs compared to other cell types. Notably, Meuleman et al. 2013 compared fLADs to cLADs, where fLADs are defined based on variability between cell types. Therefore, our results are concordant with the published work describing high AT content in LADs* [*[5,6]*](https://paperpile.com/c/xf8ftO/pGcU+eFOo)*. These results have been added to the manuscript in Supp. Fig. 3F.*

**Response Fig. 11. GC content measurement for LADs across the atlas.** GC content across HMM-LADs identified in Atlas cell types shows that T1-LADs have the lowest GC-content, followed by T2-LADs and nonLADs.

3b. The authors mention (Page 7, line 45) that T1 and T2 domain sizes vary greatly, yet this is not particularly reflected in the reported summary statistics. Perhaps **a** box or violin plot would better convey these perceived differences, or lead to the conclusion that this statement is too strong.

*We have revised this specific statement to be less descriptive and more specific to the identified LAD regions. We have also provided violin plots of the LAD sizes to better showcase the full range of T1- and T2-LAD sizes across the atlas (Response Fig. 12), as the reviewer suggested. These data are now shown in Supplementary Fig. 2E.*

**Response Fig. 12: LAD sizes across atlas cell types.** Range of LAD sizes (log2 bp shown on X-axis) per cell type and LAD type shown across the atlas.

4. Differences between T1 and T2 LADs. The authors convincingly show that T1 and T2 LADs differ in terms of various characteristics, ncluding level of Lamin B1 association, gene density and expression, etcetera. However, I have a number of concerns with some of the spatial localization analyses related to this.

4a. First, to investigate whether the radial positioning differs between T1 and T2 LADs, the authors use IF-FISH to visualize the spatial positioning of a small number of loci in ES cells and cardiac myocytes. The validity and interpretability of such an experiment depends heavily on how these loci were selected. Can the authors elaborate on this procedure? Do these loci perhaps represent e.g. the most confidently identified T1/T2 LADs?

*We apologize for our oversight in properly explaining the probe selection parameters and have revised the text to better reflect this information (page 11-12). For FISH probe design, we first categorized T1-LADs and T2-LADs by relative LB1 strength (i.e., LB1 enrichment normalized for length). We then selected regions for probe design in the 20-70th percentile range, in an effort to study representative LADs and eliminate the strongest and weakest features, which could bias the interpretability. We filtered these LADs for regions that were suitable for FISH probes (based on G/C content, melting and annealing temperatures, etc.) and tested multiple probes within this range for ESCs and cardiac myocytes. To better present this information, we now include the relative percentile strength of all the probes used for FISH analysis in Supp. Fig. 9A, F; also shown below in Response Fig. 13.*

**Response Fig. 13: FISH probes are designed in the 20th-70th percentile range of LAD strength.** The relative LB1 strength of each FISH probe shown within each category of LADs per cell type (ESCs on the left, CMs on the right).

4b. Related, what do these loci look like in Lamin DamID data, e.g. the selected T2 locus in human ESCs (chr12:65.7-67.9Mb, Fig. 4A)?

*Below, we show track views for all of the ESC FISH probes from Fig. 4 with DamID tracks included from ESCs (Response Fig. 14). As expected based on the comparisons shown in the response and in updated supplemental figures, DamID signal is prominent in T1-LADs and at a reduced level in T2-LADs. This data is now included in the manuscript as Supp. Fig. 9B.*

**Response Fig. 14: DamID signal shown for ESC FISH probes in the LAD atlas.** Probes in FISH experiments for ESCs shown with ESC-matched DamID tracks and LADs (top) overlaying ChIP-seq tracks and T1-/T2-LAD calls (bottom). Probe locations indicated in blue.

4c. If T1 and T2 LADs are truely different types of LADs, and not merely less/more dynamic ones, such loci might show a different picture in DamID, since DamID does not result in a static snapshot but rather an accumulation of Lamin association over time, which might saturate in T1 LADs and not (yet) in T2 LADs. **I**f so, does DamID data indeed show a clearer binary LAD/non-LAD pattern? Could this explain any observed differences between DamID and ChIP-seq data? If so, this would be an attractive feature of the current study and its data and is worth pointing out.

*We thank the reviewer for this interesting comment. Our analysis in response to the reviewer’s comments indicates that there is a higher DamID signal in T1-LADs than T2-LADs (see response to Point 1b and Response Fig. 1B, which is shown again below). This is consistent with the reviewer’s comment that T1-LADs may saturate DamID signal, but not T2-LADs. By track view, DamID appears to create a more binary LAD pattern (see response for Point 1b and Response Fig. 1A)., Our genome-wide quantitative analysis does show weaker DamID signal in regions of T2-LADs compared to T1-LADs.*

**Response Fig. 1:** **Tier 1 ESC DamID and ESC ChIP-seq LADs are highly overlapping.** (A) Top panel: DamID (top track in each panel) and ChIP-seq (bottom track) LB1 and LADs shown across Chr. 3. Shaded boxes highlight regions of low DamID signal that are identified as LAD regions in the DamID dataset. Bottom panel: Zoomed-in view of the highlighted area shows low DamID signal and DamID LADs over regions of low ChIP-seq LB1 signal. (B) Quantification of input-normalized DamID LB1 enrichment in ChIP-seq LADs (left) and DamID-defined LADs (right) and show a range of DamID LB1 enrichment above nonLAD signal.

4d. Regarding Figure 4C, I'm confused by the selection of the additional two loci that seemingly span T1 and T2 LADs in either ESC or cardiac myocytes. If the objective is to probe for the spatial localization of T2 LADs, why further complicate things by introducing more uncertainty in terms of LAD 'types'?

*We thank the reviewer for pointing this out and we fully agree that the loci imaged in Fig. 4C were unnecessarily complicated. Therefore, we replaced the data in the old Fig. 4C with a new T1-LAD and T2-LAD probe for ESCs (Response Fig. 7A) and CMs (Response Fig. 7B), and show comparison of radial position for all probes in both cell types together (Response Fig. 7C). These data are included in the revised manuscript as Supp. Fig. 9A-C (ESC), Supp. Fig. 9F-H(CM), and Fig. 4D, respectively. We observe a clear distinction of T2-LAD probes compared to T1-LAD probes (note the mean distances for T1-LADs versus T2-LADs for all probes in both cell types in Response Fig. 7C).*

**Response Fig. 7: T2-LADs are intermediately positioned between T1-LADs and nonLADs.** New T1- and T2-LAD probes (replacing the probes used in the previous Fig. 4C) in ESCs (A) and cardiac myocytes (B) show a similar pattern of intermediate localization between T1-LADs and nonLADs, relative to the nuclear lamina. Distance of all T1-, T2-, and nonLAD probes in this study for ESC and cardiac myocytes shown together in (C) and as a cumulative distribution frequency.

4e. The statement that T2 LADs are due to different radial positioning rather than the result of dynamic positioning (page 12) is an intriguing one and warrants some more exposure. If indeed the case, how do T2 LADs obtain their Lamin B1 association? Does this imply that Lamin B1 occurs in large enough quantities outside of the nuclear rim, as opposed to H3K9me2 (Supp. Fig. 6A)?

*We thank the reviewer for this comment. We do not have any evidence that LAMIN B1 localizes in measurable quantities outside of the nuclear rim. We have measured the thickness of the lamina by confocal and super resolution microscopy to be approximately 300nm. In all cell types we have studied* [*[4,7]*](https://paperpile.com/c/xf8ftO/TKgF+Xrca)*, including those in the current manuscript, the LB1 and H3K9me2 signal on the inner side of the nucleoplasm is of varying thickness and therefore the depth of those signals is not uniform around the nuclear circumference (see the undulations on the inner surface of the lamina marked by LB1 in Response Fig. 15A). Moreover, we assay radial positioning by measuring the distance between the center of the FISH focus and center of the nearest lamina signal to reduce bias introduced from over/undersaturation of LAMIN B1 signal intensity during imaging and deconvolution and resulting from the lamins “undulations.” If we measured the minimal distance between the edge of the FISH focus and the edge of the lamina signal (Response Fig. 15B), we would decrease most of our measurements by at least 220nm (150nm, which is half the lamina thickness + 70nm, which is half of the FISH focus diameter).*

**Response Fig. 15: Center to center (focus to LB1 signal) quantification avoids bias due to LAMIN B1 signal undulation.** (A) 3D rendering of LB1 signal shows varying thicknesses of lamina, marked by LB1 signal. (B) Comparison of edge-to-edge versus center**-**to-center distance measurements underscores the differences in edge-to-edge versus center-to-center signal quantification.

*Noting this, we would expect that T2-LADs, even in their intermediate positioning, are sufficiently proximal to the nuclear periphery to be crosslinked with a short crosslinker for ChIP, resulting in quantifiable LB1 enrichment across millions of cells, in which cells are LB1-proximal, but not often “in or at” the ring of LB1 signal. The consistently closer distance of T1-LAD foci to the LB1 signal ring similarly likely explains why T1-LAD regions have a higher amount of LB1 enrichment by ChIP across a population of millions of cells. Unfortunately, technical challenges have not allowed us to perform simultaneous H3K9me2 immunofluorescence and DNA FISH; however, the fraction of alleles within the average “cloud” of H3K9me2 signal is clearly increased when assaying T2-LADs compared to nonLADs (Fig. 4D). Thus, we do not interpret our data to suggest a large amount of LB1 exists outside of the H3K9me2 “layer.” We have edited our methods in the text to provide more clarity here.*

5a. Potential role of T2-LADs. The authors suggest that T2-LADs may be a transition step (between non-LADs and T1-LADs) for gene activation and expression (pages 13, 14 and elsewhere). This is an interesting possibility, and although the authors have attempted to visually represent this in Figure 6C, I feel like this would be conveyed better by focusing on loci that actually change LAD-status, since the majority of LAD calls remains stable in Fig. 6C.

*We have revised the existing Fig. 6C to show only the loci that actually change LAD status (Response Fig. 16) and now include in the manuscript as Supplemental Fig. 10A.*

**Response Fig. 16: Many genes that change LAD designation “move through” T2-LADs.** Amended Fig. 6C panel showing the change of locus assignment between nonLADs, T1-LADs, and T2-LADs across mesoderm lineage cells.

5b. Related, to what extent is this also reflected in transitions between genomic loci? Concretely, do the HMM state transition parameters perhaps conceptually (also) look like e.g. "non-LAD <--> T2-LAD <--> T1-LAD", and can this be shown in a separate figure?

*We thank the reviewer for this comment. Indeed, the HMM transition parameters show the "non-LAD <--> T2-LAD <--> T1-LAD" pattern described by the reviewer, and we have incorporated this into a new Supplementary Fig. 4 in the revised manuscript, also shown below (Response Fig. 17, top). For completeness, we also calculated a parallel assessment for KDDs (Response Fig. 17, bottom; new Supp. Fig. 7).*

**Response Fig. 17: HMM-designated LAD states are stable.** Top (purple): HMM transition parameters from each cell type-specific model (log2-transformed for visualization purposes) for LADs show stability of domains in the designated state. Bottom (green): HMM transition parameters from each cell type-specific model (log2-transformed for visualization purposes) for KDDs show stability of domains in the designated state.

6a. Variability across cell types. Interestingly, as opposed to previous studies based on smaller numbers of cell types, the authors find that most LADs vary across the 12 cell types assayed here. To investigate whether this is simply a matter of more exhaustively sampling cell type space, or a more inherent to the T1/T2 LAD subtype designation, it would be good to plot the percent invariance as a function of number of randomly sampled cell types. So for a random sample of, say, 4 cell types a level of invariance of XX% percent is observed, while for a random sample of, say, 8 cell types that invariance reduces to XX%.

*We thank the reviewer for raising this interesting point. We performed LAD invariance comparisons across all possible combinations of 2-12 cell types (Response Fig. 8). As the reviewer predicted, the percent of invariance is reduced as the number of cell types compared increases. For example, when comparing any 2 cell types, 26% of the genome that was assigned a state is assigned T1-LAD in both cell types, on average, whereas when comparing all 12 cell types, only 7.9% of the assigned genome is T1-LAD in all 12. Moreover, as the number of comparisons increases, T1-LADs remain relatively invariant as compared to T2-LADs. We have now expanded the discussion of these results in the manuscript (new Fig. 5B).*

**Response Fig. 8: LAD invariance increases as the number of cell types increases.** Fraction of invariant LADs across all possible comparisons from 2-12 cell types shows decreased invariance as cell type numbers increase, with T1-LADs being more invariant than T2-LADs.

6b. Related to this, are T1-LADs more likely to remain stable across (any two given) cell types than T2-LADs? If so, how do T2-LADs relate to the 'facultative LADs' described in Meuleman et al. 2013? If there is not a strong relation there, the possibility of T2-LADs being more dynamic within a cell population should more clearly be considered, as opposed to suggesting otherwise (page 12).

*Based on our multiple assessments of variance, we expected T1-LADs to remain more stable in any cell type comparison compared to T2-LADs. To confirm, we performed 2-cell type comparisons across the atlas dataset and plotted the odds ratio of pairwise comparisons (Response Fig. 18). When comparing every possible pair of cell types from our atlas, we found significantly higher invariance for T1-LADs than T2-LADs. Thus, we conclude that T1-LADs are more likely to remain stable across any two given cell types than T2-LADs. This data is included in Fig. 5C.*

**Response Fig. 18: T1-LADs are more invariant than T2-LADs across the atlas.** Cell type pair comparisons of T1-LAD and T2-LAD variation show significantly higher odds of remaining in a T1-LAD state across all possible combinations of cell type pairs compared to T2-LADs.

*We next assessed the relationship of T2-LADs to variable LADs (vLADs) defined in Meulman et al, 2013 and facultative LADs (fLADs) defined in Kind et al, 2015 (Response Fig. 19) as the reviewer suggested. We performed a “liftover” of the syntenic DamID LAD region calls onto the human hg38 genome for bp to bp overlap comparisons with our ESC HMM-identified ChIP-seq T2-LADs. We found that 37.7% of T2-LAD basepairs in ESCs overlapped with vLADs (defined from a comparison of four cell types). Given the inherent limitations of liftover analyses across species and the difference in number of cell types assayed, we also compared T2-LADs to facultative LADs (fLADs) defined in the 9-cell-type human DamID LAD comparison referenced by Reviewer 1* [*[8]*](https://paperpile.com/c/xf8ftO/ycbY)*. Here, we found that 71.0% of T2-LAD basepairs in ESCs overlapped with fLADs, likely because of the greater variety of cell types profiled compared to the mouse datasets. In support, it has previously been shown that fLADs have less LB1 contact frequency than cLADs but more than constitutive nonLADs* [*[8]*](https://paperpile.com/c/xf8ftO/ycbY)*. fLADs and vLADs are defined by differential LAD categorization between cell types, whereas T2-LADs are defined within each atlas cell type; some T2-LAD regions remain stably defined as a T2-LAD across atlas datasets. Given the differences in the methods by which T2-LADs and v/fLADs are defined, without additional studies outside the scope of this manuscript, we associate - but do not equate - T2-LADs to fLADs and vLADs. The ChIP-seq to human DamID overlap is shown in the updated manuscript as Supp. Fig. 8D.*

**Response Fig. 19: T2-LADs are related to vLADs/fLADs.** Overlap analysis of HMM-identified LADs with mouse vLADs (left) and human fLADs (right) shows a higher proportion of T2-LAD overlap with v/fLADs compared to cLADs in both datasets.

*To clarify, our discussion of the “dynamic” nature of T2-LADs is in reference to the radial positioning of T2-LADs relative to T1-LADs in the same cell type. We surmised that a T2-LAD region could show reduced LB1 enrichment in a population-based genomic assessment because that LAD was “dynamically” vacillating between a LAD and nonLAD state. A second possibility is that the T2-LAD is consistently positioned farther away from the nuclear periphery. We observed the latter possibility - that multiple T2-LAD regions in different cell types were consistently positioned in an intermediate radial positioning between the most peripheral LAD chromatin and nonLAD regions. This supports the idea of T2-LADs 1) being distinctly positioned from T1-LADs within an individual cell type and 2) not vacillating between a LAD/nonLAD binary state in a single cell type. This intermediate positioning of tested T2-LAD regions is independent of the “dynamics” of T2-LAD variation/invariance across cell types. Indeed, we observe that T2-LADs are less invariant than T1-LADs across cell type comparisons (Response Figs. 9, 10, and 18). We thank the reviewer for helping us improve the description of these efforts, to clearly present two distinct sets of comparisons: 1) the relative stability of different LAD states between cell types and 2) the relative radial positioning of LAD subtypes to each other within a single cell type.*

7. Although the authors present an impressive array of cell types, in neatly defined differentiation trajectories, most analyses rely on ESCs and caridac myocytes, which to this reviewer seems to be a bit of a missed opportunity. What is the reason for this?

*Given the comments provided in this review, we have updated the manuscript in multiple places to include several new analyses that include comparisons of LADs between cells in related developmental trajectories and across developmental trajectories, notably in the updated Figs. 5 and 6. We also underscore atlas-wide analysis efforts in Figures 2D, 3A, 3E, and 4. Moreover, the various cell types from the atlas represent both terminal and intermediate developmental stages. For some analyses, we were limited by the availability of additional data from matched cell types to make meaningful biological comparisons, noting that genome-wide datasets are limited for cells in various developmental trajectories at intermediate developmental stages. Our familiarity with cardiomyocytes and excellent differentiation marker availability made it a prudent second cell type to assay for FISH-related phenotypes.*

8. Gene Ontology (GO) enrichment analyses. The authors describe the use of GO enrichment analyses to assess the type of genes located in invariant T1 and T2 LADs (page 13), against a background of all human (protein-coding) genes. However, this will bias results towards genes that are generally located inside LADs. Rather, using a background consisting of only genes inside (T1 or T2) LADs would be more appropriate.

*We have performed this analysis again, with the revision requested, noting the concern for potential bias. We have adjusted the text and the presentation of the data on page 15 accordingly. The updated manuscript includes the revised GO enrichment analysis (updated Table 4).*

[1. Wang Y, Zhang Y, Zhang R, van Schaik T, Zhang L, Sasaki T, et al. SPIN reveals genome-wide landscape of nuclear compartmentalization. Genome Biol. 2021;22:36.](http://paperpile.com/b/xf8ftO/hVU1)

[2. Poleshko A, Shah PP, Gupta M, Babu A, Morley MP, Manderfield LJ, et al. Genome-Nuclear Lamina Interactions Regulate Cardiac Stem Cell Lineage Restriction. Cell. 2017;171:573–87.e14.](http://paperpile.com/b/xf8ftO/AwRc)

[3. Shah PP, Lv W, Rhoades JH, Poleshko A, Abbey D, Caporizzo MA, et al. Pathogenic LMNA variants disrupt cardiac lamina-chromatin interactions and de-repress alternative fate genes. Cell Stem Cell. 2021;28:938–54.e9.](http://paperpile.com/b/xf8ftO/Vg4m)

[4. Shah PP, Lv W, Rhoades JH, Poleshko A, Abbey D, Caporizzo MA, et al. Pathogenic LMNA variants disrupt cardiac lamina-chromatin interactions and de-repress alternative fate genes. Cell Stem Cell. 2021;28:938–54.e9.](http://paperpile.com/b/xf8ftO/Xrca)

[5. Meuleman W, Peric-Hupkes D, Kind J, Beaudry J-B, Pagie L, Kellis M, et al. Constitutive nuclear lamina-genome interactions are highly conserved and associated with A/T-rich sequence. Genome Res. 2013;23:270–80.](http://paperpile.com/b/xf8ftO/pGcU)

[6. Zheng X, Kim Y, Zheng Y. Identification of lamin B-regulated chromatin regions based on chromatin landscapes. Mol Biol Cell. 2015;26:2685–97.](http://paperpile.com/b/xf8ftO/eFOo)

[7. Poleshko A, Shah PP, Gupta M, Babu A, Morley MP, Manderfield LJ, et al. Genome-Nuclear Lamina Interactions Regulate Cardiac Stem Cell Lineage Restriction. Cell. 2017;171:573–87.e14.](http://paperpile.com/b/xf8ftO/TKgF)

[8. Kind J, Pagie L, de Vries SS, Nahidiazar L, Dey SS, Bienko M, et al. Genome-wide maps of nuclear lamina interactions in single human cells. Cell. 2015;163:134–47.](http://paperpile.com/b/xf8ftO/ycbY)

**Second round of review**

**Reviewer 1**

I acknowledge, and very much appreciate, the efforts the authors made to address my concerns. However, I still have significant concerns regarding 1) the novelty of the findings (see below) and more importantly 2) the domain calling and especially the annotation of the T2-LAD class (see also below).

Novelty

“Another advance is our identification of two types of LADs within a single cell type with different epigenetic and molecular characteristics. As we present in greater detail below (see response to Point 5 and to Rev. 2, Point 6b), T2-LADs partially overlap and share similarity with previously-described facultative/variable LADs. However, we do not equate T2-LADs to fLADs/vLADs because T2-LADs are not defined by LAD changes between cell types.”

Zheng et al (2015) classified LADs in two types (within the same cell type) based on differences in LB1-values and assigned differences in chromatin signatures to both types.

“Another strength of our work is examining how population-based LAD states impact single-cell LAD radial positioning.”

Kind et al (2015) showed that the single-cell frequency with which a genomic region is in contact with the lamina is directly proportional to the log2 Dam-LB1/Dam values observed in population data. I am sure this also holds true for ChIP-seq as it holds true for e.g. HiC.

Domain calling

The 3-state HMM model may be fitting the variability in the data best, yet should a genomic region that is not enriched over the control (untethered Dam or input) be called an interaction, or in this case a T2-LAD? This region is not enriched over background and should therefore not be considered to have no interaction with the lamina. Many of the regions that are considered T2-LADs are on or below baseline in ChIP. The majority of the regions are below baseline in DamID. In my strong opinion it is incorrect to assign these regions as LADs.

“It is important to note that the LB1 ChIP-seq occupancy threshold varies along the genome; if it did not vary, then a simple threshold model would work to identify domains. This is the motivation for utilizing the HMM, which can annotate genomic regions where LB1 occupancy is shifted to a sufficient degree from surrounding regions to warrant differential domain categorization.”

This comment from the rebuttal letter I can’t agree with and is further cause of concern of the validity of the 3-state HMM model. ChIP-seq occupancy values should not vary along the genome. A log2 enrichment over control should be interpreted the same whether it concerns a region on chromosome 3 over chromosome 6. Sure, the overall dynamic range of a profile may be different between experiments in different cell types, yet within the same experiment a baseline should be considered a baseline along the entire genome. This is exactly my concern with the 3-state HMM. It is calling domains that involve transitions between a log2-FC domain of negative 0.5 and 0. Yes, 0 is higher than negative 0.5 but it does not mean this region is associated with the lamina. This is also observed from fig. 3c in which a large proportion of the T2-LADs have contact frequencies between single cells that are close to 0. Similar to the distribution of the non-LADs.

Sup. 3c. The comparison of DamID and ChIP is not exactly reassuring. E.g. the regions on the left of the zoom-in tracks do not correspond at all. The almost entire ~2.5Mb regions is clearly below baseline for DamID, whereas for ChIP most of the regions are above baseline (assuming the y-axis labeling is faulty). These regions are all annotated as T2-LADs which is solely based on ChIP-seq and not substantiated by DamID. Of course, the question what may be the gold standard here, but since the distinction between T1- ad T2-LADs is such a focus of this manuscript, caution about the difference in annotation is justified.

This cautionary note is further substantiated by Fig. S3e. Note that in the boxplot with DamID-intensity values (log2 LB1/input(?)) for the T2-LADs 75% of the datapoints is below the baseline of 0. Thus, in any DamID dataset, 75% of the here annotated T2-LADs would be considered non-LADs because there is no enrichment of the Dam-LB1 construct over the Dam control. This complicates any conclusions drawn based on the T2-LADs identified by ChIP-seq.

Fig. S3c. I am convinced that the ChIP-seq data by and large correspond to the DamID in mESCs. Yet, the percentage overlap of >99% in domains (based on the 3-state HMM) between DamID and ChIP-seq really worries me especially since Figs. S3C and S3E (see previous comment) show that a high percentage of T2-LAD annotation correspond to log2 DamID-values that are on- or below baseline.

I would very much like to advise the authors to revisit the domain calling. Currently, it is near impossible to value/interpret the findings attributed to the T2-LADs because of the uncertainty whether these are true lamina-interacting regions or not.

Minor points

Fig. S2e The non-LADs (grey)are missing in the graph.

Fig. S3c The axis for the ChIP ranges from -1.2 (top) to -0.9 (bottom). I assume this is a mistake.

Line 58 page 7. The control for DamID normalization is a dataset in which an untethered Dam enzyme is expressed. This controls for differential genomic chromatin accessibility. The text reads:”input-normalized”. Is the DamID data normalized to ChIP input control? I assume not, but this needs clarification. Same for the labeling in Fig. S3.

**Reviewer 2**

The authors have performed a substantial amount of work to include additional analysis results and figures, which have greatly improved the quality and clarity of the manuscript.

This work continues to represent a valuable data resource for the chromatin biology and genome organization communities, and the authors have addressed most of my concerns.

There are a few outstanding issues I highlight below.

1.Quality of generated data and concordance with previously published data Although I appreciate the extra effort the authors have put in addressing this point, I distinctly referred to Pearson correlation coefficient is NOT being an appropriate metric for data quality and concordance. Rather, I was suggesting the use of an analysis more in line with what the authors describe in their response to point 1b ("bp to bp overlap").

Regarding Response Figure 1B: what is the reason for showing ciLADs and not "ESC iLADs"?

2. Number of distinct lamina-related states As for Point 1, I'd like to explicitly see HMM call concordance, and not Pearson correlation coefficients.

3. General characteristics of T1, T2 and non-LADs

Regarding Response Figure 12: I was very confused by the range of y-axis shown here. Can the authors clarify why this range is larger than the boxplots shown?

4. Differences between T1 and T2 LADs Same clarifying question regarding Response Figure 1B (see Point 1). I furthermore appreciate the additional analyses and clarifications here, which make the distinction between T1 and T2 LADs, and importantly between T2 LADs and fLADs, more clear.

I think it is important to even more strongly emphasize the difference between T2-LADs and fLADs, in the sense that the former are defined within a single experiment / cell type, while the latter reflects differences between experiments / cell types.

5. Potential role of T2-LADs

Thanks for the clarification and additional figures and analyses.

6. Variability across cell types

In my opinion there is no need to differentiate between "vLADs" and "fLADs". fLADs were originally defined in Meuleman et al. 2013, initially using mouse data. The concept was re-applied in Kind et al. on human datasets. The term vLADs is not used in either study and is likely to confuse readers, in particular in addition to already partially competing concepts T2-LADs and fLADs.

Regaring Point 6a, the newly added analysis and corresponding figure raise a number of interesting questions. For one, should we expect ciLADs to plateau at ~10% invariance with increasing numbers of cell types? If so, is this 10% particularly high in terms of A/T sequence content? As a clarifying question: it was not immediately clear to me what the beige line ("LADs") represents, assuming it is not simply T1+T2-LADs, as such would not immediately result in a summation of percentage invariance?

7. Breadth of analyses

Thanks for expanding on this.

8. Gene Ontology (GO) enrichment analyses

Thanks for correcting this, it has improved the results and interpretation in my opinion, including a tantalizing potential "role" for T2 LADs in providing general background/housekeeping transcription/functionality. It would be interesting to see if this is reflected in their general transcription levels, too.

**Authors Response**

**Point-by-point responses to the reviewers’ comments:**

*We again thank both reviewers for the rigorous reviews, which have greatly improved this manuscript and our presentation of this data resource. We appreciate both reviewers’ acknowledgement of our efforts to address concerns and questions in the previous review. Below, we provide additional point-by-point responses to the remaining comments.*

Reviewer reports:

Reviewer #1: I acknowledge, and very much appreciate, the efforts the authors made to address my concerns. However, I still have significant concerns regarding 1) the novelty of the findings (see below) and more importantly 2) the domain calling and especially the annotation of the T2-LAD class (see also below).

Novelty

“Another advance is our identification of two types of LADs within a single cell type with different epigenetic and molecular characteristics. As we present in greater detail below (see response to Point 5 and to Rev. 2, Point 6b), T2-LADs partially overlap and share similarity with previously-described facultative/variable LADs. However, we do not equate T2-LADs to fLADs/vLADs because T2-LADs are not defined by LAD changes between cell types.”

Zheng et al (2015) classified LADs in two types (within the same cell type) based on differences in LB1-values and assigned differences in chromatin signatures to both types.

“Another strength of our work is examining how population-based LAD states impact single-cell LAD radial positioning.”

Kind et al (2015) showed that the single-cell frequency with which a genomic region is in contact with the lamina is directly proportional to the log2 Dam-LB1/Dam values observed in population data. I am sure this also holds true for ChIP-seq as it holds true for e.g. HiC.

*We respectfully acknowledge the reviewer’s concern about the novelty. We previously cited and discussed the findings of Zheng et al, Kind et al, and other seminal studies within the context of our own results. On page 4, we stated, “Comparison of datasets from various mouse and human cell types indicate that LADs can be segregated into cell type-variable (facultative) and stable (constitutive)*. *Integration of lamin association with core and linker histone occupancy and modifications also distinguished subtypes of LADs within a single murine cell type.” In the updated manuscript, we have redoubled efforts to ensure that we are not assigning inappropriate novelty to the dataset, further underscoring where our results support existing models of LAD biology and where advances are possible within this multi-cell type human dataset (see pages 5, 7, and 14).*

*Regarding this concern, Rev. 2 stated: “The novelty aspect does not weigh that strongly for me: although more DamID LaminB1 data have been generated, much of it was never published, as pointed out by the manuscript authors. I think as a data resource there is novelty and utility here. In addition, the authors make it clear that the proposed T2 LADs are defined within a cell type, as opposed to previously described "facultative LADs", which show variation across cell types.” Also, “This work continues to represent a valuable data resource for the chromatin biology and genome organization communities...”*

*Therefore, we respectfully disagree that this manuscript lacks sufficient novelty for publication in* Genome Biology*.*

Domain calling

The 3-state HMM model may be fitting the variability in the data best, yet should a genomic region that is not enriched over the control (untethered Dam or input) be called an interaction, or in this case a T2-LAD? This region is not enriched over background and should therefore not be considered to have no interaction with the lamina. Many of the regions that are considered T2-LADs are on or below baseline in ChIP. The majority of the regions are below baseline in DamID. In my strong opinion it is incorrect to assign these regions as LADs.

*We thank the reviewer for providing a detailed explanation for this concern. As alluded to by the reviewer, any region of the genome has a probability of being associated with the lamina. This probability likely differs across cells and is also influenced by the fraction of the ‘domain’ which is in contact with the periphery. As presented in the manuscript, and further assessed below, T2-LADs are defined by LB1 enrichment and are not equivalent to nonLADs. Multiple lines of evidence indicate that T2-LADs represent an intermediate, peripherally-associated chromatin state identified by weaker lamina-associations compared to canonical T1-LADs. We agree that T2-LADs are not equivalent to T1-LADs and do not associate with the nuclear lamina in the same way as T1-LADs; however, these features are defined by LB1 enrichment, and our data robustly demonstrate that T2-LADs cannot be considered nonLADs****.***

*In light of the reviewer’s comments, in order to reflect these differences and ensure that the nomenclature is not misrepresenting the results, we propose using the name “intermediate peripheral domain (iPD)” in lieu of “T2-LAD” for regions enriched with LB1 that are distinct from both nonLADs and highly LB1-enriched T1-LADs. This would make it clear that the regions that we are currently calling T2-LADs are distinct from those that we are currently calling T1-LADs and nonLADs. If the reviewers and editors are enthusiastic about this name change, we would be glad to implement it. We have already adjusted the text throughout the manuscript to indicate that the second subtype is an intermediate LB1-enriched domain.*

*In addition, we conducted new analyses to clarify that T2-LADs are distinct from nonLADs. Multiple lines of evidence demonstrate the robustness of T2-LADs and their peripheral association. Firstly, the majority of T2-LAD regions are enriched for LB1 (Fig. 1C). We now make this point more clearly by providing an assessment of the percentage of HMM-identified genome segments with a log2 LB1/input ratio > 0 from each biological ChIP-seq replicate. This data confirms that most T2-LAD regions are enriched for LB1 occupancy relative to input (Response Fig. 1). This LB1:input enrichment is clearly distinct from ChIP-seq signal in nonLADs.*

*Next, we tested if normalization was falsely impacting the calculation of LB1 enrichment in T2-LADs. Specifically, if T2-LADs show LB1 enrichment at the level of nonLADs, it is possible that input normalization might falsely result in regions appearing to be LB1 enriched. This is an important point, because it impacts the interpretation of our normalized data. Therefore, we assessed LB1 and input read counts in T2-LADs and nonLADs, using ESCs and cardiac myocytes (CM) as representative cell types. The median unnormalized level of LB1 ChIP-seq reads is significantly higher in T2-LADs compared to nonLADs. In addition, nonLADs are not enriched for LB1 compared to input (Response Fig. 2). Thus, artificial enrichment by input normalization cannot explain the LB1 occupancy observed in T2-LAD regions.*

Percent

***Response Fig. 1: T1- and T2-LADs, but not nonLADs are enriched for LB1.*** Percent of T1-LAD, T2-LAD, and nonLAD 20kb bins with log2 ChIP/input > 0 shown for each replicate across atlas cell types. The majority of T1- and T2-LADs, but not nonLADs, show LB1 enrichment by this measure.

***Response Fig. 2: T2-LADs have LB1 enrichment independent of input.*** LB1 reads assigned to T2-LADs and nonLADs show standalone significant enrichment in T2-LADs compared to nonLADs in ESCs (top, left) and CMs (bottom, left) (One-tailed Mann– Whitney U test: ESC U=6.337x109, p<2.2x10-16; CM U=6.337x10^9^, p < 2.2x10^16^). nonLADs are not LB1 enriched compared to input for either cell types (top and bottom, right).

*These new figure panels are included in the updated manuscript as Supp. Figs. 3A and 3B, respectively.*

*Next, we sought to address the reviewer’s concern that our HMM is not properly identifying domains and may be a biased contributor to our identification of T2-LADs. To do so, we reanalyzed our data using epic2, a ChIP-seq peak caller, to independently identify regions of LB1 enrichment in the ESC and CM LB1 ChIP-seq data, in accord with our previous efforts (Shah et. al., 2021)*. *If T2-LADs are a distinct domain feature, independent of the method used to call LADs, then 1) epic2 peaks should map to T1- and T2-LADs, but not nonLADs, and 2) epic2 peaks overlapping T2-LADs should be smaller peaks on account of T2-LADs containing more gaps in the LB1 profile compared to T1-LADs. We identified 24,737 and 18,083 epic2 LB1 peaks (q < 0.05) in ESC and CMs, respectively (Response Fig. 3). In ESCs, 72.9% of the peaks mapped to T1-LADs and 23.4% mapped to T2-LADs. Of the remaining peaks, 3.6% mapped to regions spanning both a T1- and T2-LAD and less than 0.1% (15 peaks) mapped to nonLADs. In CMs, 77.1% epic2 LB1 peaks mapped to T1-LADs and 18.5% mapped to T2-LADs. Of the remaining peaks, 4.4% mapped to regions spanning both T1- and T2-LADs and less than 0.01% (1 peak) mapped to nonLADs. Moreover, the median size of the T1-LAD epic2 LB1 peaks is larger than T2-LADs for both cell types (25.5kb and 29kb for T1-LADs compared to 13kb and 14.5kb for T2-LADs in ESCs and CMs, respectively). These analyses support the finding that T2-LADs are enriched for LB1 and are not equivalent to nonLADs.*

***Response Fig. 3: T1-LAD and T2-LADs are enriched in epic2 LB1 peaks.*** Nearly all LB1 peaks identified by epic2 map to T1-LADs and T2-LADs in ESC and CMs. A subset of peaks overlap both types of LADs in both cell types, with fewer than 0.1% mapping to nonLADs.

*The difference in peak length suggested that T2-LADs feature many smaller regions of LB1 enrichment compared to larger, more consistent regions of LB1 enrichment in T1-LADs.*

***Response Fig. 4: T2-LADs have more gaps in LB1 profile than T1-LADs.*** The proportion of T1-LAD,T2-LAD, and nonLAD bins enriched for LB1 (log2 ChIP/input > 0) for a range of bin sizes shows a decrease in proportion of enriched T2-LADs bins as size increases in ESCs (top) and CMs (bottom). Regardless of bin size, LB1 is enriched in T2-LADs independent of nonLADs in both cell types.

*Therefore, we directly assessed for gaps in LB1 profile in ESCs and CMs (Response Fig. 4). We calculated the proportion of each HMM-identified feature that has log2 LB1/input > 0 across a range of bin sizes for ESCs and CMs. If T2-LADs are distinct from nonLADs and have more gaps in LB1 signal than T1-LADs, we would expect that 1) T2-LADs would have LB1 enrichment distinct from nonLADs in all window sizes and 2) that T2-LADs would show decreased proportion of features with LB1 signal greater than input as window size increases (and gaps become incorporated into the signal calculation). Both of these expectations were met in this analysis. The mean proportion of bins reduced from 38% and 44% in 100bp windows to 32% and 31% in 10kb windows for ESC and CM, respectively. Gaps are an expected feature of LADs as previously published (Lund et. al., 2014), and likely, in part, reconcile why a subset of LADs can be identified by LB1 enrichment but have an overall per feature log2 LB1/input ratio less than 0. This important analysis clarifies that a major difference between LAD subtypes is that T2-LADs tend to contain a mixture of regions with high levels of LB1 and gaps with lower levels, making their overall LB1 enrichment levels lower than T1-LADs. This new analysis is provided in the updated manuscript as Supp. Fig. 8E.*

*Corroborating these bulk genomics data, our FISH assays in single cells clearly indicate that T2-LAD regions are localized proximal to the nuclear periphery and again distinct from nonLADs. In aggregation across all cell types and probes, 27% T2-LAD compared to <2% of nonLAD FISH foci were within the layer of peripheral heterochromatin, as measured by the depth of H3K9me2. As described above, this “intermediate” positioning likely reflects cell-to-cell heterogeneity and a smaller fraction of the domain being directly lamina associated.*

*As a final measure of the confidence in the HMM calls, we performed bp-by-bp overlap and concordance analyses, as requested by Reviewer 2. If T2-LADs are at the level of noise, we would expect concordance in only T1-LADs. However, our results show a high degree of replicability across HMM-identified features and cell types (Response Fig. 5), which is included in the updated manuscript as Supp. Fig. 3C (LADs) and Supp. Fig. 6C (KDDs).*

***Response Fig. 5: HMM-identified features are highly replicable across atlas cell types.*** The percent of overlapping bp from replicate HMM results for LADs (A) and KDDs (B) across the atlas show a high degree of replicability in all features.

*Taken together, these assessments demonstrate the following:*

*1)* *LB1 enrichment over input in the majority of T2-LAD regions,*

*2)* *LB1 enrichment in T2-LADs compared to nonLADs with and without input normalization,*

*3)* *the identification of LB1 enrichment in T2-LADs using epic2 defined ChIP-seq peaks,*

*4)* *the presence of gaps in T2-LAD LB1 profiles, and*

*5)* *the high concordance of replicate HMM-identified features, including T2-LADs.*

***These results all strongly argue in favor of T2-LADs being a distinct LB1-defined feature compared to nonLADs.***

“It is important to note that the LB1 ChIP-seq occupancy threshold varies along the genome; if it did not vary, then a simple threshold model would work to identify domains. This is the motivation for utilizing the HMM, which can annotate genomic regions where LB1 occupancy is shifted to a sufficient degree from surrounding regions to warrant differential domain categorization.”

This comment from the rebuttal letter I can’t agree with and is further cause of concern of the validity of the 3-state HMM model. ChIP-seq occupancy values should not vary along the genome. A log2 enrichment over control should be interpreted the same whether it concerns a region on chromosome 3 over chromosome 6. Sure, the overall dynamic range of a profile may be different between experiments in different cell types, yet within the same experiment a baseline should be considered a baseline along the entire genome. This is exactly my concern with the 3-state HMM. It is calling domains that involve transitions between a log2-FC domain of negative 0.5 and 0. Yes, 0 is higher than negative 0.5 but it does not mean this region is associated with the lamina. This is also observed from fig. 3c in which a large proportion of the T2-LADs have contact frequencies between single cells that are close to 0. Similar to the distribution of the non-LADs.

*We thank the reviewer for highlighting the lack of clarity in our explanation. We apologize for this confusion and would like to clearly state that we chose an HMM approach with the expectation that LB1 occupancy differs across the genome within a single cell type, in accord with previous studies (Zheng et. al., 2015). Thus, if it is possible for LADs to be defined in any state beyond a “binary” definition (LAD/nonLAD), this justifies using an HMM without a priori knowledge of how many states there will be and what levels of LB1 will segregate those states.*

*Regarding the transitions, we agree that a simple transition in signal may not reliably identify a LAD subtype. We also agree with the reviewer that multiple analyses and validations are required to properly interpret the differences highlighted by the genomics. As described in detail above, our analyses indicate that T2-LADs are enriched for LB1, cannot be considered equivalent to nonLADs, and represent an intermediate peripheral domain. Moreover, our FISH analyses clearly indicate that T2-LAD regions associate with the lamina. The difference between T1-LADs and T2-LADs is reflected by variation across single cells and the fraction of the domain which is associated with the periphery.*

Sup. 3c. The comparison of DamID and ChIP is not exactly reassuring. E.g. the regions on the left of the zoom-in tracks do not correspond at all. The almost entire ~2.5Mb regions is clearly below baseline for DamID, whereas for ChIP most of the regions are above baseline (assuming the y-axis labeling is faulty). These regions are all annotated as T2-LADs which is solely based on ChIP-seq and not substantiated by DamID. Of course, the question what may be the gold standard here, but since the distinction between T1- ad T2-LADs is such a focus of this manuscript, caution about the difference in annotation is justified.

*The grey boxes shown in the inset in the former Supp. Fig. 3C (now Supp. Fig. 3E) highlight regions where there are DamID-identified LADs that overlap regions defined as T2-LADs. Therefore, it appears that “weaker” LADs are identifiable, though to a lesser degree, in the DamID dataset. More importantly, the T2-LADs demonstrate more DamID/Dam control signal than nonLADs.*

*There are myriad reasons why regions with fewer or weaker LB1 contacts or more gaps (defined above) may not be as easily identified in DamID-mapped LADs versus ChIP-seq-mapped LADs. We have outlined a number of these methodological differences in the manuscript (see pages 6 and 23), including the use of an enzymatic reaction over a period of time versus crosslinking at a specific time, differences in the number of cells used and line-to-line, and differences in analysis pipelines. A particularly notable difference is that ChIP-seq requires crosslinking, which may stabilize weaker LB1-DNA interactions making them more easily mapped by this method. Therefore, lamina-associated regions that have gaps in the LB1 profile may be captured more consistently in one method versus the other. Therefore, it is necessary to validate such LAD features by FISH and other methodologies to ensure that LB1 signal is not noise, which we do and provide in the manuscript and above in this response.*

This cautionary note is further substantiated by Fig. S3e. Note that in the boxplot with DamID-intensity values (log2 LB1/input(?)) for the T2-LADs 75% of the datapoints is below the baseline of 0. Thus, in any DamID dataset, 75% of the here annotated T2-LADs would be considered non-LADs because there is no enrichment of the Dam-LB1 construct over the Dam control. This complicates any conclusions drawn based on the T2-LADs identified by ChIP-seq.

Fig. S3c. I am convinced that the ChIP-seq data by and large correspond to the DamID in mESCs. Yet, the percentage overlap of >99% in domains (based on the 3-state HMM) between DamID and ChIP-seq really worries me especially since Figs. S3C and S3E (see previous comment) show that a high percentage of T2-LAD annotation correspond to log2 DamID-values that are on- or below baseline.

*We respectfully disagree that limited DamID enrichment in T2-LADs prevents drawing conclusions from T2-LADs for the reasons discussed above, notably that ChIP-seq and DamID each have limitations, that T2-LADs can be validated as being peripherally proximal and that T2-LADs have LB1 enrichment independent of T1-LADs. Most importantly, T2-LADs demonstrate more DamID signal than nonLADs, and thus cannot easily be equated to noise. Since we did not produce the DamID data, we cannot speak to the details of those data; perhaps if the Dam reaction was lengthened and/or other optimizations made, weaker LB1 contacts could be more readily identified. We have described above the various reasons why the overall signal of any domain may appear depleted for log2 ChIP/input or DamID/Dam control, including cell-to-cell heterogeneity and the fraction of the domain which is directly contacting the lamina. Having said this, we appreciate the reviewer’s concern and propose changing the name of T2-LADs to “intermediate peripheral domain (iPD)” to reflect the intermediate LB1 occupancy in these regions, if the reviewers and editor are enthusiastic about such a change.*

*Regarding the second, related point*, *the domain overlap is a bp-by-bp overlap assessment between the HMM-identified ChIP-seq LADs and the DamID-identified LADs, as defined within that dataset. Considering the concern raised by the reviewer, we have revisited this analysis, performed the overlap again to ensure that we did not err in our initial efforts. Our updated analyses, shown below (Response Fig. 6), confirm that nearly all DamID bp overlap T1- or T2-LADs (99.6%). Moreover, this stratified analysis shows that 47.4% of T2-LAD bp are shared with a DamID-identified LADs while 0.1% of nonLAD bp identified by ChIP-seq are shared with DamID-identified LADs. These data argue against the idea that T2-LADs are not represented in LADs identified by another method. The stark-difference in the T2- and nonLAD overlap with DamID suggests that HMM-defined T2-LADs are not equivalent to nonLADs*

I would very much like to advise the authors to revisit the domain calling. Currently, it is near impossible to value/interpret the findings attributed to the T2-LADs because of the uncertainty whether these are true lamina-interacting regions or not.

*We deeply appreciate the reviewer’s insightful comments. We hope that they appreciate the additional validations for the LB1 enrichment in T2-LADs, as described above, most of which is incorporated into the updated manuscript. We revisited the domain calling with epic2 and performed new nucleotide-level and feature-level comparisons with DamID. The data from these analyses strongly support T2-LADs being a feature identified by LB1 enrichment, distinct from nonLADs, and separate from T1-LADs. Reviewer 2 states “I...appreciate the additional analyses and clarifications here, which make the distinction between T1 and T2 LADs, and importantly between T2 LADs and fLADs, more clear.” We therefore respectfully disagree that there is no value/interpretable findings in T2-LADs, when these features clearly represent a lamina-proximal subset of chromatin identifiable by LB1 contacts by multiple methods across a broad range of cells. However, as discussed above, we propose changing the term T2-LAD to “intermediate peripheral domain (iPD)” to better present the analyses in this manuscript and to emphasize that T2-LADs should not be confused with T1-LADs. We hope our efforts and response addresses the reviewer’s concerns, but if we are not fully understanding their concerns – we would appreciate the opportunity to better address them.*

Minor points

Fig. S2e The non-LADs (grey)are missing in the graph.

*We thank the reviewer for the comment and have modified the graph.*

Fig. S3c The axis for the ChIP ranges from -1.2 (top) to -0.9 (bottom). I assume this is a mistake.

*Thank you for pointing this out; the error has been corrected.*

Line 58 page 7. The control for DamID normalization is a dataset in which an untethered Dam enzyme is expressed. This controls for differential genomic chromatin accessibility. The text reads:”input-normalized”. Is the DamID data normalized to ChIP input control? I assume not, but this needs clarification. Same for the labeling in Fig. S3.

*We apologize for the lack of clarity and this has been edited on the relevant graphs.*

Reviewer #2

The authors have performed a substantial amount of work to include additional analysis results and figures, which have greatly improved the quality and clarity of the manuscript.

This work continues to represent a valuable data resource for the chromatin biology and genome organization communities, and the authors have addressed most of my concerns.

*We thank the reviewer for these kind words and the comments below. The concerns and suggestions raised by the reviewer have consistently improved the manuscript.*

There are a few outstanding issues I highlight below.

1.Quality of generated data and concordance with previously published data

Although I appreciate the extra effort the authors have put in addressing this point, I distinctly referred to Pearson correlation coefficient is NOT being an appropriate metric for data quality and concordance. Rather, I was suggesting the use of an analysis more in line with what the authors describe in their response to point 1b “"bp to bp overla”").

*We apologize for our misunderstanding here. We have performed the analyses as suggested (bp to bp comparison). Our results show a high degree of replicability across HMM-identified features and cell types by both measures, noting again that the midbrain samples show the least replicability of all the samples, as discussed in the previous review (Response Figs. 5 and 7). The overlap analysis in Response Fig. 5 are included in the updated manuscript as Supp. Fig. 3C (LADs) and Supp. Fig. 6C (KDDs) in lieu of the Pearson correlation plots.*

***Response Fig. 5: HMM-identified features are highly replicable across atlas cell types.*** The percent of overlapping bp from replicate HMM results for LADs (A) and KDDs (B) across the atlas show a high degree of replicability in all features.

Regarding Response Figure 1B: what is the reason for showing ciLADs and not “ESC iLADs”?

*This is a nomenclature error on our part. We identified all nonLAD regions in this dataset by omitting the LADs from the mappable genome, and the correct term for these would indeed be iLADs. We have corrected this error. We thank the reviewer for helping us to correct this mistake.*

2. Number of distinct lamina-related states

As for Point 1, I’d like to explicitly see HMM call concordance, and not Pearson correlation coefficients.

*We have performed this analysis, as presented above.*

3. General characteristics of T1, T2 and non-LADs

Regarding Response Figure 12: I was very confused by the range of y-axis shown here. Can the authors clarify why this range is larger than the boxplots shown?

*This is an error. When generating this figure, we presented the data on a log2 scale, but failed to correct the scale size, which led to the extended Y-axis. This has been corrected (Response Fig. 8). We thank the reviewer for identifying this issue.*

4. Differences between T1 and T2 LADs

Same clarifying question regarding Response Figure 1B (see Point 1).

*This is a nomenclature error on our part, which we have corrected.*

I furthermore appreciate the additional analyses and clarifications here, which make the distinction between T1 and T2 LADs, and importantly between T2 LADs and fLADs, more clear.

I think it is important to even more strongly emphasize the difference between T2-LADs and fLADs, in the sense that the former are defined within a single experiment / cell type, while the latter reflects differences between experiments / cell types.

*We thank the reviewer for this suggestion and have emphasized these points on pages 11 and 18.*

5. Potential role of T2-LADs

Thanks for the clarification and additional figures and analyses.

*We are glad these were clarifying.*

6. Variability across cell types

In my opinion there is no need to differentiate between“"vLAD”" and“"fLAD”". fLADs were originally defined in Meuleman et al. 2013, initially using mouse data.The concept was re-applied in Kind et al. on human datasets. The term vLADs is not used in either study and is likely to confuse readers, in particular in addition to already partially competing concepts T2-LADs and fLADs.

*We agree; this is unnecessarily confusing, and we have changed the labels accordingly.*

Regaring Point 6a, the newly added analysis and corresponding figure raise a number of interesting questions. For one, should we expect ciLADs to plateau at ~10% invariance with increasing numbers of cell types? If so, is this 10% particularly high in terms of A/T sequence content? As a clarifying question: it was not immediately clear to me what the beige line “"LAD”") represents, assuming it is not simply T1+T2-LADs, as such would not immediately result in a summation of percentage invariance?

*We thank the reviewer for raising this interesting point. We also noted that the percent of invariant T1-LADs plateaus at around 10% of the mappable genome (i.e., blacklisted regions*

*removed), and would expect that this percentage would remain similar with more cell types, given the plateau. We assessed of the AT composition in invariant T1-LADs and found that these regions have a median of 60% AT content, which is a slight increase (but not significant) compared to 58% for the genome (Response Fig. 9). This is now included in the manuscript at Supp. Fig. 10A.*

*To further clarify, “LADs” represents T1-LADs and T2-LADs grouped together in Fig. 5 (reproduced below, Response Fig. 10). The beige line represents the percent of all 20kb HMM calls that are LADs (T1-LADs and T2-LADs) in N cell types compared to all of the regions considered a LAD (T1-LAD and T2-LAD) across all cell types. The plot in the manuscript (Response Fig. 10A) shows the percent invariance of T1-LAD, T2-LAD, or both LAD subtypes with respect to the mappable genome. Therefore, the denominator represents the universe of T1-LADs and/or T2-LADs and remains the same for all the comparisons in the analysis. Thus, the beige line indeed results in a summation of the two purple lines. We clarified this point in the figure legend.*

*For completeness, we also replotted the invariance with respect the number of cell types compared (Response Fig. 10B). For example, for T1-LADs, we plotted the percent of invariant T1-LADs out of all the T1-LADs in at least one of the compared cell types. In this plot, the denominator changes with the number of cell types being compared. That is, there are more regions that are T1-LAD in at least one of 10 cell types versus regions that are T1-LAD in at least one of 2 cell types. In this case, the beige line (still representing T1- and T2-LADs grouped together) does not result in a summation of the purple lines. Because the denominator changes with each comparison, we feel the interpretation is much less intuitive (and does not change the conclusion).*

*Therefore, we have provided this version only in the response, and we did not include it in the updated manuscript.*

7. Breadth of analyses
Thanks for expanding on this.

*We are glad this was helpful.*

8. Gene Ontology (GO) enrichment analyses
Thanks for correcting this, it has improved the results and interpretation in my opinion, including a tantalizing potential "role" for T2 LADs in providing general background/housekeeping transcription/functionality. It would be interesting to see if this is reflected in their general transcription levels, too.

*Thank you for your input and expertise on these points. We agree with your suggestion to see if transcript levels reflect a more general role of invariant T2-LADs across cell types. We looked at the expression of genes that overlap invariant T2-LADs compared to a background measure of T2-LADs that are shared between at least 2 cell types (Response Fig. 11). As expected, we observe a similar proportion of expressed genes (tpm > 0.5) in invariant T2-LADs across cell types for which we have expression data (left). Interestingly, there is more variability in gene expression in varying T2-LADs across cell types (right). Overall, we find that this transcriptional trend reflects the less likely cell type-specific role of invariant T2-LADs compared to invariant T1-LADs (see page 16), but agree that this is an exciting area for future exploration of invariant T2-LADs.*

**Third round of review**

**Reviewer 1**

The authors have clarified some important points and adressed many other concerns. I have no further comments

**Reviewer 2**

The authors have satisfactorily addressed my remaining concerns.
